# Network analysis reveals miRNA crosstalk between periodontitis and oral squamous cell carcinoma

**DOI:** 10.1186/s12903-022-02704-2

**Published:** 2023-01-13

**Authors:** Zhengrui Li, Rao Fu, Xutao Wen, Ling Zhang

**Affiliations:** 1 Department of Oral and Maxillofacial-Head and Neck Oncology, Shanghai Ninth People’s Hospital, Shanghai Jiao Tong University School of Medicine, College of Stomatology, Shanghai Jiao Tong University, Shanghai, China; 2 National Center for Stomatology and National Clinical Research Center for Oral Diseases, Shanghai, China; 3 Shanghai Key Laboratory of Stomatology, Shanghai, China

**Keywords:** Network analysis, MicroRNA, Genetic crosstalk, Periodontitis, Oral squamous cell carcinoma

## Abstract

**Background:**

Oral squamous cell carcinoma (OSCC) is one of the malignant tumors with a poor prognosis. Periodontitis (PD is considered a high-risk factor for OSCC, but the genetic mechanism is rarely studied. This study aims to link OSCC and PD by identifying common differentially expressed miRNAs (Co-DEmiRNAs), their related genes (Hub genes), transcription factors (TFs), signaling pathways, enrichment functions, and compounds, and searching for genetic commonalities.

**Methods:**

The miRNAs expression datasets of OSCC and PD were searched from the GEO database. The miRNA and related crosstalk mechanism between OSCC and PD was obtained through a series of analyses.

**Results:**

hsa-mir-497, hsa-mir-224, hsa-mir-210, hsa-mir-29c, hsa-mir-486-5p, and hsa-mir-31are the top miRNA nodes in Co-DEmiRNA-Target networks. The most significant candidate miRNA dysregulation genes are *ZNF460*, *FBN1*, *CDK6*, *BTG2*, and *CBX6*, while the most important dysregulation TF includes HIF1A, TP53, E2F1, MYCN, and JUN. 5-fluorouracil, Ginsenoside, Rh2, and Formaldehyde are the most correlated compounds. Enrichment analysis revealed cancer-related pathways and so on.

**Conclusions:**

The comprehensive analysis reveals the interacting genetic and molecular mechanism between OSCC and PD, linking both and providing a foundation for future basic and clinical research.

**Supplementary Information:**

The online version contains supplementary material available at 10.1186/s12903-022-02704-2.

## Introduction

Oral squamous cell carcinoma (OSCC) is one of the most common head and neck malignant tumors, with a high incidence of 350,000 new cases and 170,000 deaths [1]. The Asian region has the highest OSCC morbidity and mortality among all other countries, which various etiological factors may cause. The risk factors of OSCC recognized by researchers include smoking [2], drinking [3], chewing betel nut [4], periodontal disease [5], gene mutation [6], and so on. However, periodontal diseases represented by periodontitis (PD) have played an increasingly significant role in the occurrence and development of OSCC [7, 8].

There is a convincing correlation between inflammation and the occurrence and development of many cancers [9–11]. The role of inflammation in tumors can also be observed in the oral environment [12]. PD also is strongly associated with a variety of tumors, including breast cancer [13], pancreatic cancer [14], gastric cancer [15], and colorectal cancer [16]. To date, PD is considered one of the most common inflammatory conditions affecting the oral cavity and one of the risk factors for OSCC [17]. Systematic reviews have confirmed the previous association between OSCC and PD [18], but further mechanism study of the shared connotation still needs to be completed. In particular, little is known about the possible epigenetic mechanisms of OSCC and PD.

MicroRNA (miRNAs) are small RNAs that play vital roles in regulating gene expression. miRNAs regulate cellular physiological processes by regulating expression and play a crucial role in mediating diseases [19]. The molecular mechanism of disease occurrence and development can be further grasped by studying disease-related miRNAs and their expression patterns. In addition, miRNAs can serve as biomarkers of two diseases (OSCC and PD) or key targets for therapeutic drugs [20].

This study aimed to comprehensively analyze differentially expressed miRNAs (DEmiRNAs) in OSCC and PD to identify candidate CO-DEmiRNAs, their associated hub genes, signaling pathways and related compounds. Thereby promoting the understanding of the shared molecular mechanisms between closely related tumors and non-neoplastic diseases. And providing a theoretical basis for driving future basic research and clinical practice.

In more than 20 years of clinical work by our group, we found that almost all patients with OSCC suffer from periodontitis while burdening the tumor. Based on our previous translational studies on inflammation-precancerous lesion cancer, we sought to explore whether the presence of crucial genetic molecules could serve as a connecting key for PD and OSCC. Recent studies have revealed numerous noncoding RNAs (ncRNAs) roles in cancer and various diseases, highlighting the biological significance of these previously “neglected” RNA species. In particular, microRNAs (miRNAs) are involved in many biological processes that affect cell homeostasis. MiRNAs are considered post-transcriptional gene regulators that can achieve translational repression, mRNA degradation, and gene silencing and play a significant role in gene expression. We sought to explore and determine whether there are co-expressed key miRNAs and transcription factors present in PD and OSCC by bioinformatics methods, thus providing a solid basis for our subsequent target findings. This helps us in a series of studies in stomatitis-cancer transformation. We promote an understanding of the shared molecular mechanisms between closely related tumors and non-neoplastic diseases. We provide a theoretical basis for future basic research and clinical practice.

## Materials and methods

### MicroRNA datasets selection and preparation

Download the miRNA expression datasets of OSCC and PD from the GEO database (Table 1) (https://www.ncbi.nlm.nih.gov/geo/). Only one OSCC miRNA dataset GSE45238 was identified for analysis (obtained from platform GPL8179, Illumina Human v2 MicroRNA expression bead chip). For the miRNA expression dataset for PD, we chose the most numerous GSE54710 (obtained from platform GPL15159, Agilent 031181 Unrestricted Human miRNA V16.0 Microarray 030840). All experiments were performed further with relevant guidelines and regulations.

**Table 1:** The OSCC and PD miRNA datasets were used for analysis

Disease	Accession	Platform	Case	Control	Total
OSCC	GSE45238	GPL8179	40	40	80
PD	GSE54710	GPL15159	158	41	200

### Data processing and differential expression miRNA analysis

The microarray and expression data were downloaded using the R package “GEOquery” (https://www.r-project.org/). The data were corrected using the “ComBat” method in the R package “SVA.” The R package “Limma” was then used to identify miRNAs significantly differentially expressed in OSCC and PD cases and controls. miRNA (*P* value < 0.05 and |LogFC|> 0.5) are regarded as “DEmiRNA” and used for analysis. Furthermore, LogFC > 0.5 is overexpressed and LogFC < 0.5 is low expressed.

### Shared DEmiRNA analysis and Co-DEmiRNA identification

The miRNA lists of the two diseases were processed using the R package “Venndiagram” to obtain Shared DEmiRNA. These were considered mutual DEmiRNAs and were further analyzed. We defined miRNAs with the common expression trend (both high/low expression) as Co-DEmiRNAs and excluded Shared DEmiRNAs with different expression trends (their opposite expression does not assist in disease-related studies, nor is it meaningful for scientific research).

### Co-DEmiRNA-gene network construction and functional enrichment analysis

The co-DEmiRNA target network was constructed using miRNet 2.0 (https://www.mirnet.ca/). For the Co-DEmiRNA-Gene network, target genes were selected from 3 packages (TarBase v8.0), miRTarBase v8.0 (http://mirtarbase.mbc.nctu.edu.tw/php/index.php), and miRecords (http://c1.accurascience.com/miRecords). Due to poor stability, “Steiner Forest Network” cannot achieve the most stable link on the premise of ensuring the correlation. Instead, “Minimum Network” was chosen, which reduces the network complexity and retains key features that demonstrate network connectivity. It is computed using the critical nodes of all elements. To build the “Minimum Network”, the shortest paths between the nodes are determined, and any nodes not on the shortest path are removed.

### Hub gene identification and functional enrichment analysis

From the constructed Co-DEmiRNA-Gene network, we selected key gene nodes with higher degrees and betweenness as “Hub Genes” that connect other parts of this complex network. In a sense, hub genes are the actual central complex members. In addition, we performed an enrichment analysis of hub genes for KEGG [21] and GO terms. Use the R package “ggplot2” for visualization and R package “clusterProfiler” to analyze selected data. The calculated *P* values were subjected to FDR correction for KEGG and GO enrichment, using FDR ≤ 0.05 as a threshold.

### Co-DEmiRNA-TF network construction and functional enrichment analysis

We used multiple databases such as miRbase (https://www.mirbase.org/), TransmiR v2.0 (http://www.cuilab.cn/transmir) to download the corresponding transcription factors (TF) of Co-DEmiRNAs, extracted the corresponding TFs, and constructed a Co-DEmiRNA-TF Network using a similar method. The Co-DEmiRNA-TF networks were further subjected to functional enrichment analysis using the KEGG pathway, Reactome pathway, and GO.

### Co-DEmiRNA-compound network construction

Search for small-molecule compounds with a high correlation with Co-DEmiRNAs. Build the Co-DEmiRNA-compound network based on data from CTD datasets (Comparative Toxicogenomic Database, http://ctdbase.org/), miRbase (https://www.mirbase.org/), SM2miR (http://bioinfo.hrbmu.edu.cn/SM2miR/) and PharmacomIR.

### Co-DEmiRNA diagnostic efficacy analysis

Other databases were used to verify the diagnostic efficacy of the best Co-DEmiRNA as a diagnostic marker. The miRNA-seq data of the level 3 BCGSC miRNA Profiling in the TCGA (https://portal.gdc.cancer.gov/) HNSC (Head and Neck Squamous Cell Carcinoma) project were selected for verification, and the corresponding data without clinical information were discarded. Samples belonging to oral cancer sites (Alveolar Ridge, Tongue, Buccal Mucosa, Floor of mouth, Hard Palate, Oral Cavity) were retained in clinical information, and samples from nonoral cancer sites (Hypopharynx, Larynx, Lip, Oropharynx, Tonsil) were excluded. The miRNA-seq data in RPM (Reads per Million mapped reads) format was converted to log2, and 373 samples were obtained (using the R package “pROC” for data analysis and the “ggplot2” package for visualization). To calculate the area under the curve, the area value under the ROC curve should be between 0.5 and 1. The closer the AUC is to 1, the better the diagnostic effect.

The corresponding expression patterns of the two disease miRNAs are shown (Fig. 3). There were 18 shared DEmiRNAs with a similar expression trend (Table 2). 6 DEmiRNAs were co-overexpressed, while co-low expression was observed in 5 shared DEmiRNAs. The remaining seven shared DEmiRNAs showed diametrically opposite expression trends in the two diseases.

**Table 2:** The Shared DEmiRNA of OSCC and PD

Expression	DEmiRNA
Co-up	hsa-miR-224, hsa-miR-33a, hsa-miR-210, hsa-miR-1246, hsa-miR-31*, hsa-miR-31
Co-down	hsa-miR-363, hsa-miR-497, hsa-miR-140-3p, hsa-miR-29c, hsa-miR-486-5p
Opposite	hsa-miR-142-3p, hsa-miR-650, hsa-miR-211, hsa-miR-155, hsa-miR-223 hsa-miR-142-5p, hsa-miR-99a

**Results**

### DEmiRNA and shared DEmiRNA identification

In the OSCC dataset (GSE45238), the comparison was between the cases of OSCC (tumor specimens of OSCC patients) and normal cases (adjacent nontumor epithelium). After correction, 858 miRNAs were retained, and 208 OSCC-related significant DEmiRNAs were identified. In periodontitis data (GSE54710), the comparison was the case of periodontitis (periodontal tissue with periodontitis) and standard samples (healthy periodontal tissues). In contrast, PD dataset analysis retained 1368 miRNAs and identified 54 significant PD-related DEmiRNAs (Additional file 1: Table S1. a, b).

**Fig. 1:**
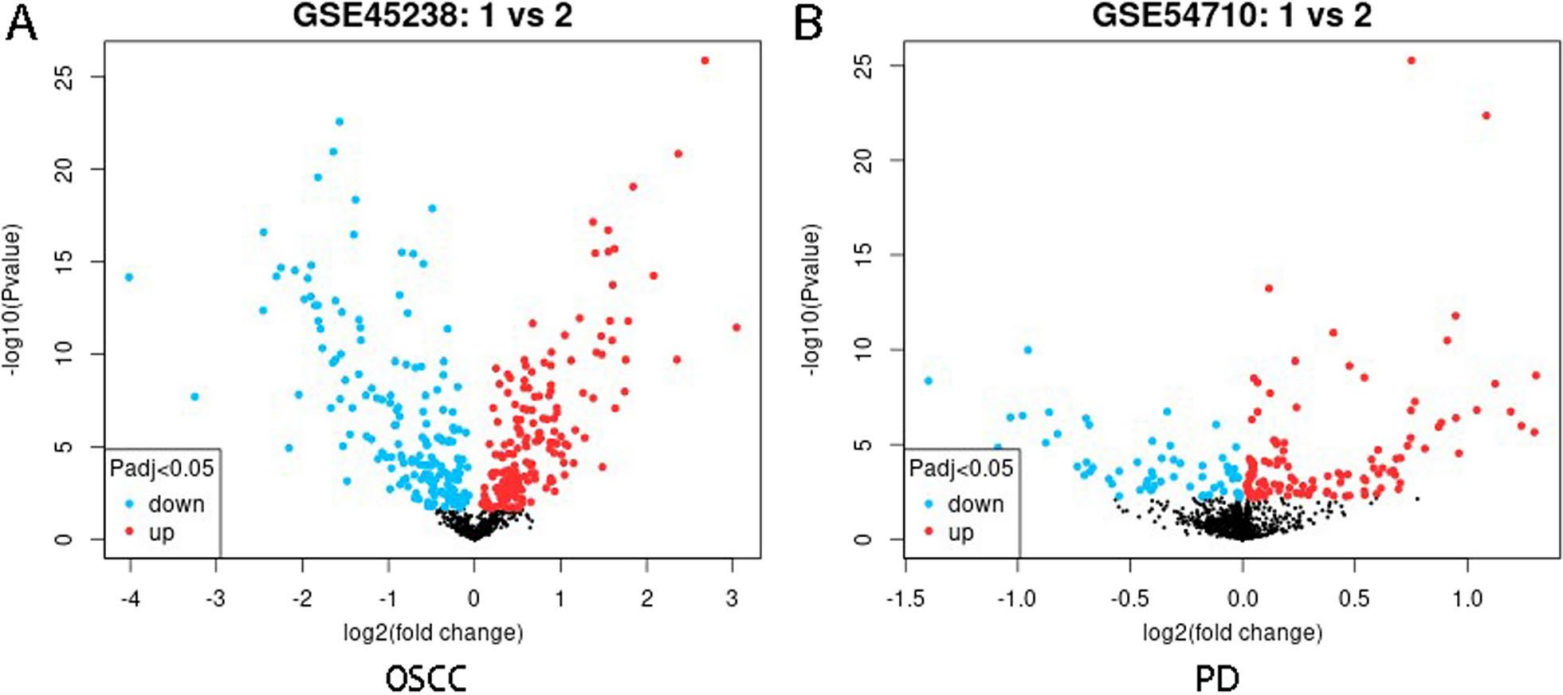
Data processing and differential expression analysis. **A**, **B** The Volcano map of OSCC and PD differentially expressed miRNA. The miRNA expression in OSCC is relatively broad and scattered, while PD is more concentrated (Blue indicates significant down-regulation, red indicates significant up-regulation, and black parts are non-statistically significant expressed genes.)

**Fig. 2:**
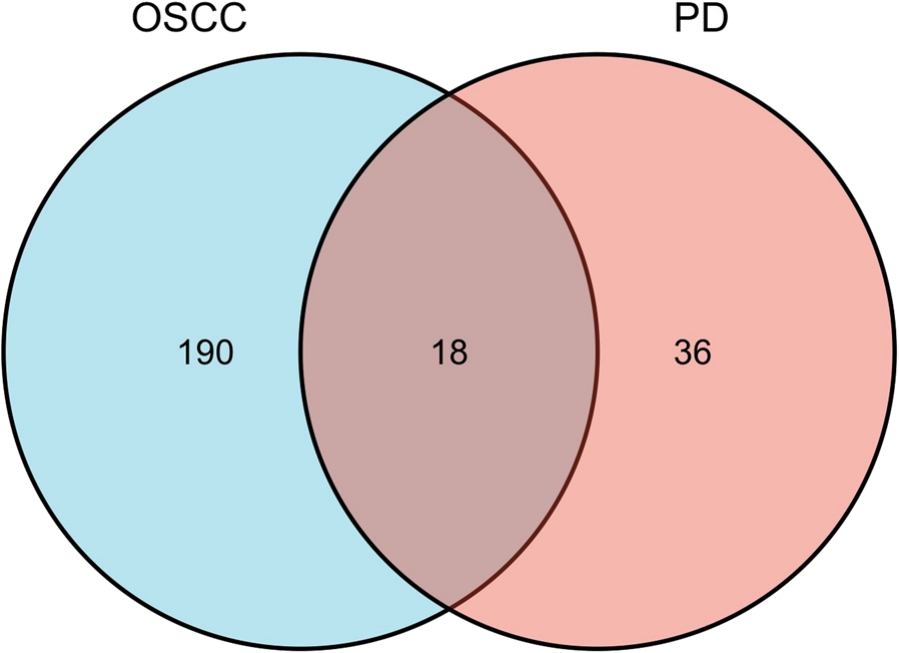
Shared DEmiRNA analysis. The intersection between the two graphs represents that there are 18 shared DEmiRNAs between OSCC (GSE45238) and PD (GSE54710)

Compared with controls in OSCC-affected tissues, 103 DEmiRNAs were overexpressed, and 105 DEmiRNAs were under-expressed (Fig. 1A). Similarly, 35 miRNAs were overexpressed in PD-affected tissues, while 19 were under-expressed (Fig. 1B). A Venn diagram searched for the common part between the two DEmiRNA lists, and 18 shared DEmiRNAs were screened (Fig. 2). We also think the direct comparison of OSCC and periodontitis is important, and this could broaden our search for common molecular genetic mechanisms for both diseases in further research.

**Fig. 3:**
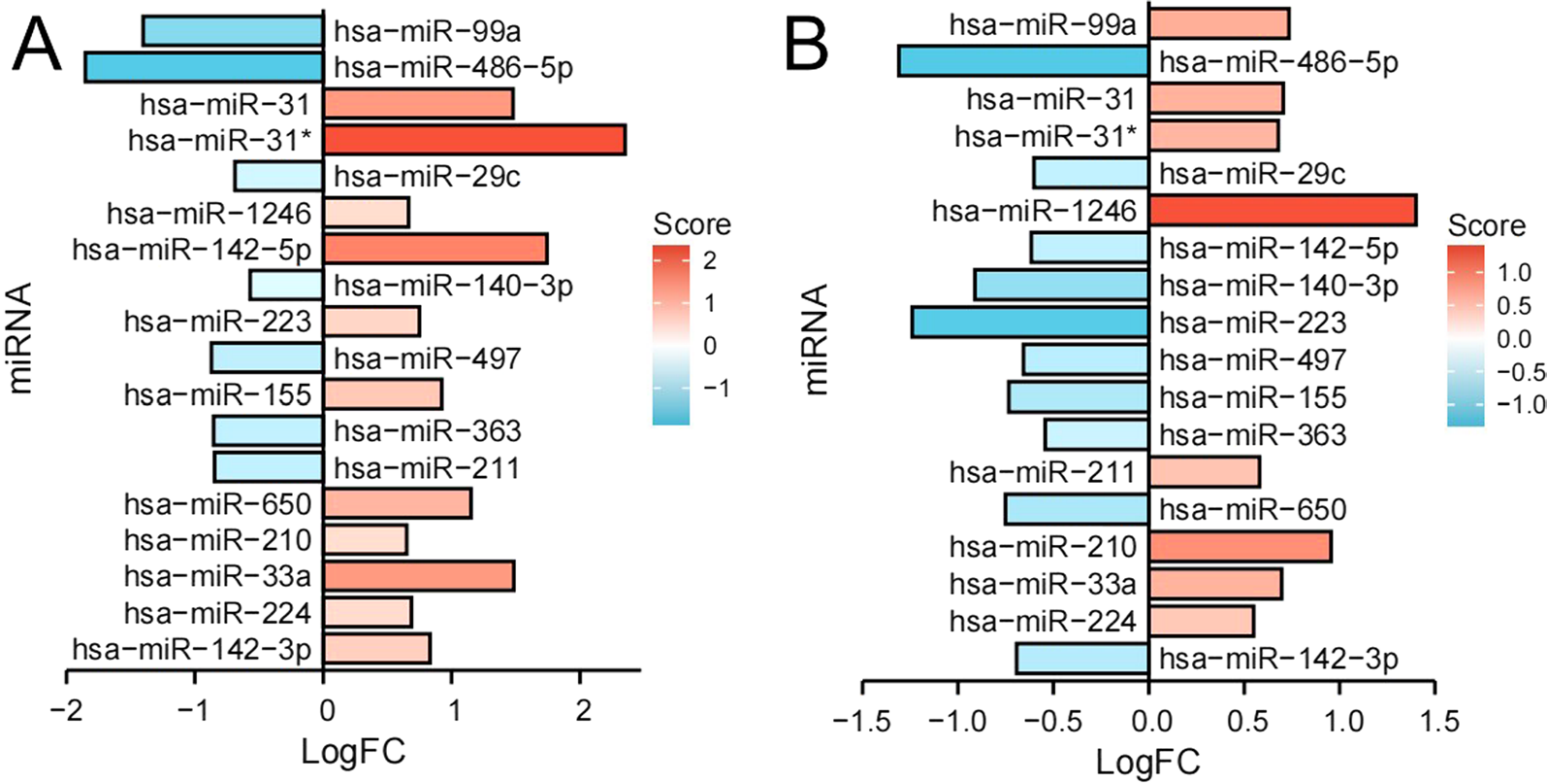
The different expression trends of DEmiRNAs in OSCC (**A**) and PD (**B**). Only six shared miRNAs showed overexpression in both diseases, and 5 showed joint underexpression. (Red represents overexpression, and blue represents low expression. When the shared miRNA has the same color, it represents a common expression trend that it is has a common expression trend in OSCC and PD)

The corresponding expression patterns of the two disease miRNAs are shown (Fig. 3). There were 18 shared DEmiRNAs with a similar expression trend (Table 2). 6 DEmiRNAs were co-overexpressed, while common low expression was observed in 5 shared DEmiRNAs. The remaining seven shared DEmiRNAs showed diametrically opposite expression trends in the two diseases.

### Co-DEmiRNA identification, Co-DEmiRNA-gene network and functional analysis

**Fig. 4:**
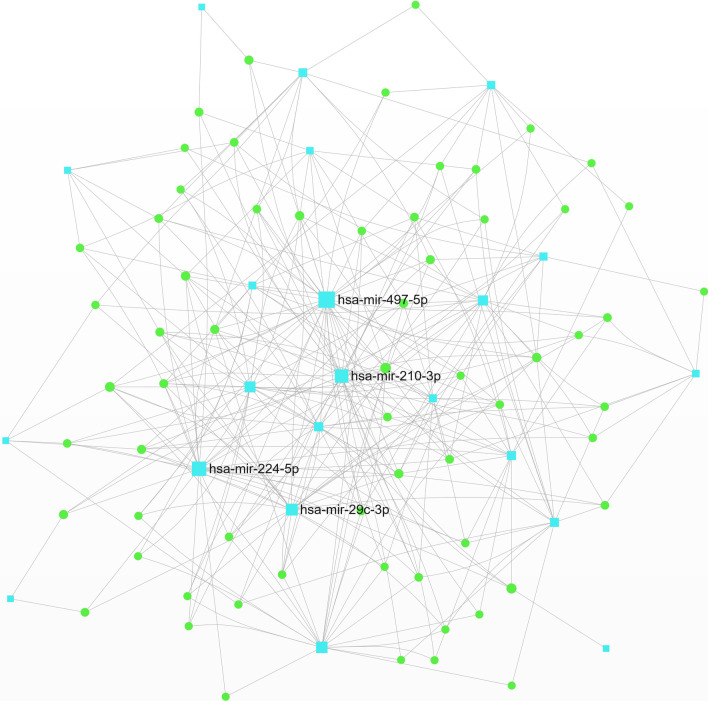
The minimum network of Co-DEmiRNA-Gene. The optimized and minimal network was used to obtain the five key miRNA nodes with the strongest centrality and the highest degree among the 11 DEmiRNAs, including hsa-mir-497-5p, hsa-mir-224-5p, hsa-mir-210-3p, hsa-mir-29c-3p, and hsa-mir-486-5p. The five most critical related gene nodes were also obtained, which were *ZNF460*, *FBN1*, *CDK6*, *BTG2*, *and CBX6* (blue node: DEmiRNA, green node: gene)

We selected 11 DEmiRNAs with the same expression trend as Co-DEmiRNAs for further analysis. They then constructed the Co-DEmiRNA-Gene Network. The minimum network consists of 63 genes and 22 miRNAs with 303 edges (Fig. 4, Additional file 1: Table S2a). The highest degree DEmiRNA nodes in the network are hsa-mir-497-5p, hsa-mir-224-5p, hsa-mir-210-3p, hsa-mir-29c-3p, hsa-mir-486-5p. The top 5 gene nodes with the highest degree in the network include *ZNF460*, *FBN1*, *CDK6*, *BTG2*, *and CBX6*. The most abundant signaling pathways are shown (Table 3. Additional file 1: Table S2b–f). KEGG pathway analysis showed Focal adhesion, ECM-receptor interaction Pathways in cancer, p53 signaling pathway, and other related pathways. Reactome analysis showed Signaling by SCF-KIT, Oncogene Induced Senescence, Pre-NOTCH Transcription Translation, PI3K/AKT pathway, and other related pathways. GO biology process (GO-BP) analysis showed negative regulation of the cellular process, Ras protein signal transduction, and so on. The most abundant GO molecular functions (GO-MF) include extracellular matrix structural constituents, transcription from RNA polymerase II promoters, and other binding-related functions, including growth factor, nucleotides, purine ribonucleotide, and purine nucleotides. The enriched top GO cellular components (GO-CC) include ruffle, nucleoplasm, cell leading edge, organelle, lumen, extracellular matrix, and other parts.

**Table 3:** Top 10 enriched pathways in the Co-DEmiRNA-gene network

KEGG pathway	Value
Focal adhesion	5.34e−05
ECM-receptor interaction	0.000264
Pathways in cancer	0.00109
Melanoma	0.00121
Small cell lung cancer	0.00222
Glioma	0.0104
p53 signaling pathway	0.0117
mRNA surveillance pathway	0.0194
TGF-beta signaling pathway	0.0207
Asthma	0.0484
Reactome pathway	
Signaling by SCF-KIT	1.43E−05
Oncogene Induced Senescence	1.70E−05
Pre-NOTCH Transcription and Translation	4.81E−05
PI3K events in ERBB4 signaling	7.45E−05
PIP3 activates AKT signaling	7.45E−05
PI3K events in ERBB2 signaling	7.45E−05
PI-3K cascade:FGFR1	7.45E−05
PI-3K cascade: FGFR2	7.45E−05
PI-3K cascade:FGFR3	7.45E−05
PI-3K cascade:FGFR4	7.45E−05
GO-BP	
Negative regulation of cellular process	1.76e−05
Ras protein signal transduction	1.78e−05
Neurogenesis	3.82e−05
Generation of neurons	6.59e−05
Response to external stimulus	0.000108
Skeletal system development	0.000116
Neuron projection development	0.000123
Nervous system development	0.000136
Regulation of cell proliferation	0.000193
Negative regulation of biological process	0.000207
GO-MF	
Growth factor binding	1.03e−05
Extracellular matrix structural constituent	1.65e−05
Transcription from RNA polymerase II promoter	0.000364
Nucleotide binding	0.00263
Protein phosphatase type 2A regulator activity	0.00346
Negative regulation of transcription DNA-dependent	0.0054
Purine ribonucleotide binding	0.00642
Purine nucleotide binding	0.00661
Protein serine/threonine kinase activity	0.00759
Protein serine/threonine/tyrosine kinase activity	0.0101
GO-CC	
Growth factor binding	1.03e−05
Extracellular matrix structural constituent	1.65e−05
Transcription from RNA polymerase II promoter	0.000364
Nucleotide binding	0.00263
Protein phosphatase type 2A regulator activity	0.00346
Negative regulation of transcription, DNA-dependent	0.0054
Purine ribonucleotide binding	0.00642
Purine nucleotide binding	0.00661
Protein serine/threonine kinase activity	0.00759
Protein serine/threonine/tyrosine kinase activity	0.0101

### Hub genes identification and enrichment function analysis

**Fig. 5:**
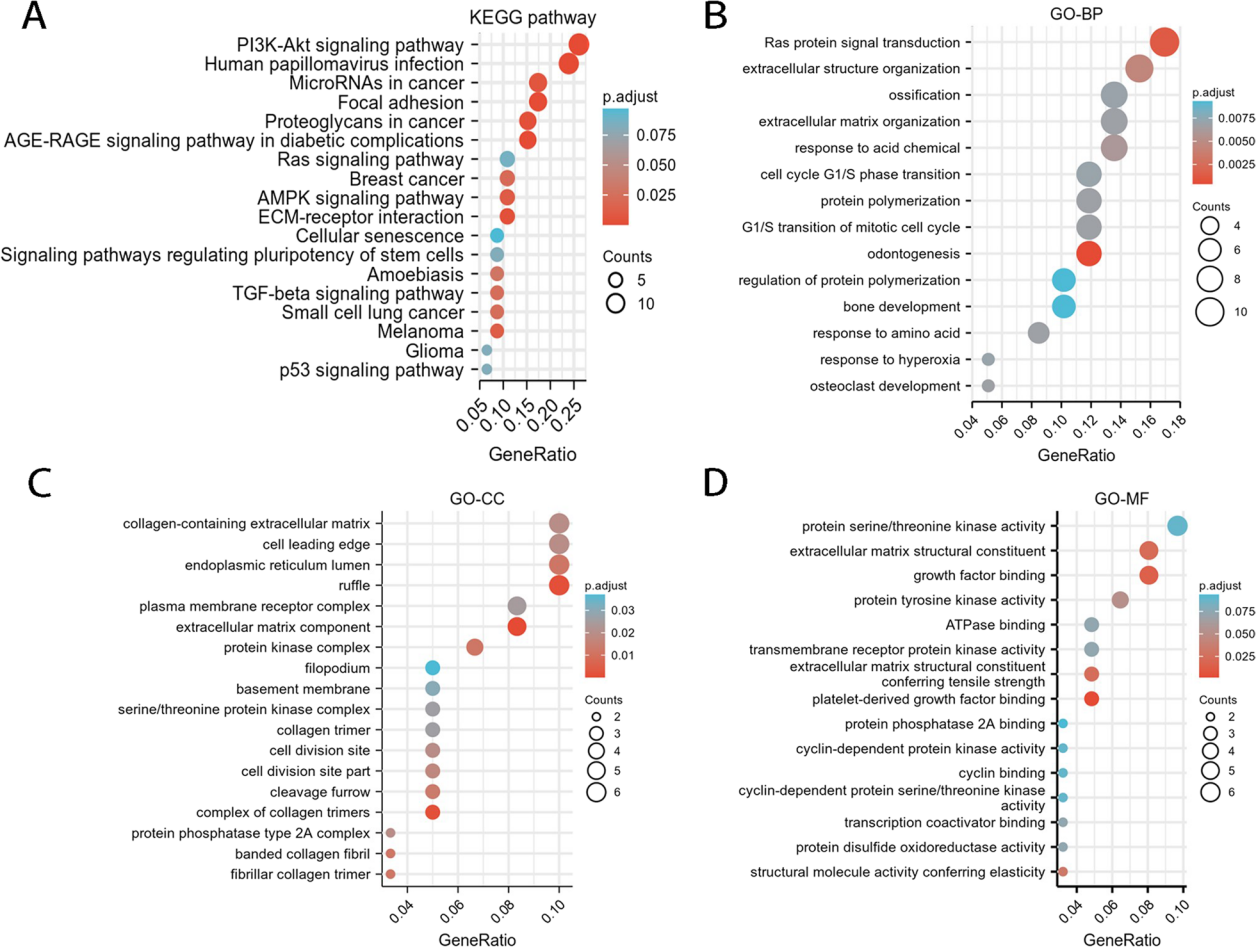
KEGG pathway and GO enrichment analysis of Hub genes related to Co-DEmiRNAs. The stronger the correlation between miRNAs and pathways, the larger the number of counts and the larger the bubbles (The *p* value is determined by color. The closer the color is to red, the smaller the *P* value. *P* < 0.05 considered statistically significant)

We obtained all the network's key node genes with statistical significance, including *ZNF460*, *FBN1*, *CDK6*, *BTG2*, *CBX6*, *DYRK1A,* and so on. We obtained some significant pathways corresponding to hub genes (Fig. 5). The figure shows that the PI3K/AKT signaling pathway was the most enriched KEGG pathway. In addition, GO-BP analysis reveals that Ras protein signal transduction and extracellular structure organization are the most enriched biological process. GO-CC analysis revealed that the enriched cellular components were collagen-containing extracellular matrix, cell leading edge, ruffle, and other parts. GO-MF analysis revealed that protein serine/threonine kinase activity, extracellular matrix structural constituents, and growth factor binding were the top enriched molecular functions.

### Co-DEmiRNA-TF network and functional analysis

**Fig. 6:**
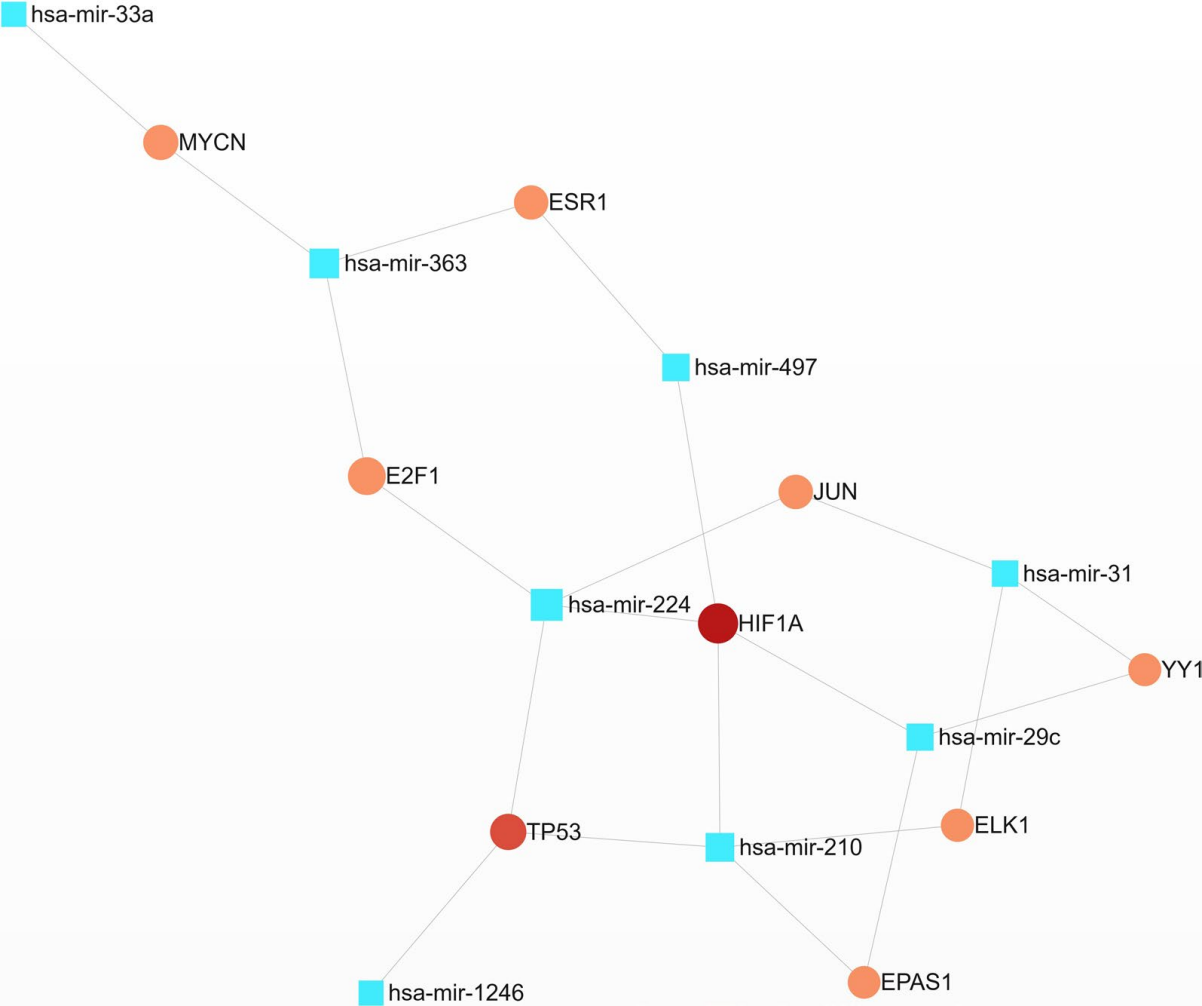
The minimum network of Co-DEmiRNA-TF. IF1A, TP53, E2F1, MYCN, and JUN are located close to the center, demonstrating that these 5 TFs are credible and central (blue node: Co-DEmiRNA, red node: TF)

The corresponding transcription factors were queried by miRbase, and the key transcription factors of Co-DEmiRNAs were obtained (Additional file 1: Table S3). The original network consists of 48 TFs, eight miRNAs, and 64 edges. The minimum network consists of 9 TFs, eight miRNAs, and 21 edges (Fig. 6, Additional file 1: Table S4a). The top 5 transcription factors include HIF1A, TP53, E2F1, MYCN, and JUN, while hsa-mir-224 and hsa-mir-210 are the topmost miRNA nodes. The most abundant KEGG, Reactome, and GO pathways are listed (Table 4, Additional file 1: Table S4b–f). These include acute myeloid leukemia, pathways in cancer, and other pathways. GO-BP analysis revealed positive regulation of transcription from RNA polymerase II promoter and DNA-dependent. GO-MF analysis in TFs includes multiple transcription-related and binding-related functions. GO-CC analysis reveals that the most enriched cellular components are transcription factors in the complex, nucleoplasm, and other parts.

**Table 4:** Top 10 enriched pathways in the Co-DEmiRNA-TF network

KEGG pathway	Value
Acute myeloid leukemia	2.72E−08
Pathways in cancer	5.71E−06
Chronic myeloid leukemia	0.000286
Epstein-Barr virus infection	0.000549
Transcriptional misregulation in cancer	0.000553
Adipocytokine signaling pathway	0.00604
Pancreatic cancer	0.00721
Small cell lung cancer	0.00961
Toxoplasmosis	0.0128
Jak-STAT signaling pathway	0.0145
Reactome pathway	
Pathways in cancer	0.000125
Legionellosis	0.00247
Renal cell carcinoma	0.00549
Adipocytokine signaling pathway	0.00604
Apoptosis	0.0103
TGF-beta signaling pathway	0.0106
MAPK signaling pathway	0.0117
Chagas disease (American trypanosomiasis)	0.0118
Toxoplasmosis	0.0128
Toll-like receptor signaling pathway	0.0139
GO-BP	
Positive regulation of transcription from RNA polymerase II promoter	1.71e−11
Positive regulation of transcription, DNA-dependent	1.52e−09
Positive regulation of transcription, DNA-dependent	1.52e−09
Positive regulation of RNA metabolic process	2.68e−09
Positive regulation of the nucleobase-containing compound metabolic process	8.02e−09
Regulation of transcription from RNA polymerase II promoter	1.69e−08
Positive regulation of cellular metabolic process	5.23e−08
Transcription from RNA polymerase II promoter	1e−07
Positive regulation of metabolic process	1.04e−07
Regulation of multicellular organismal process	1.14e−06
GO-MF	
Positive regulation of transcription, DNA-dependent	1.33e−09
Transcription from RNA polymerase II promoter	8.8e−08
Sequence-specific DNA binding	5.2e−07
Transcription factor binding	1.41e−06
DNA binding	2.8e−06
Negative regulation of transcription, DNA-dependent	6.39e−05
Chromatin binding	0.00237
Protein heterodimerization activity	0.00316
Double-stranded DNA binding	0.00649
Protein dimerization activity	0.00704
GO-CC	
Transcription factor complex	4.72e−05
Nucleoplasm	8.93e−05
Nucleoplasm part	0.000275
Nuclear lumen	0.00106
Chromatin	0.00143
Nuclear part	0.00388
Organelle lumen	0.00426
Membrane-enclosed lumen	0.00472
Chromosomal part	0.0108
Chromosome	0.0166

### Co-DEmiRNA-compound network and functional analysis

**Fig. 7:**
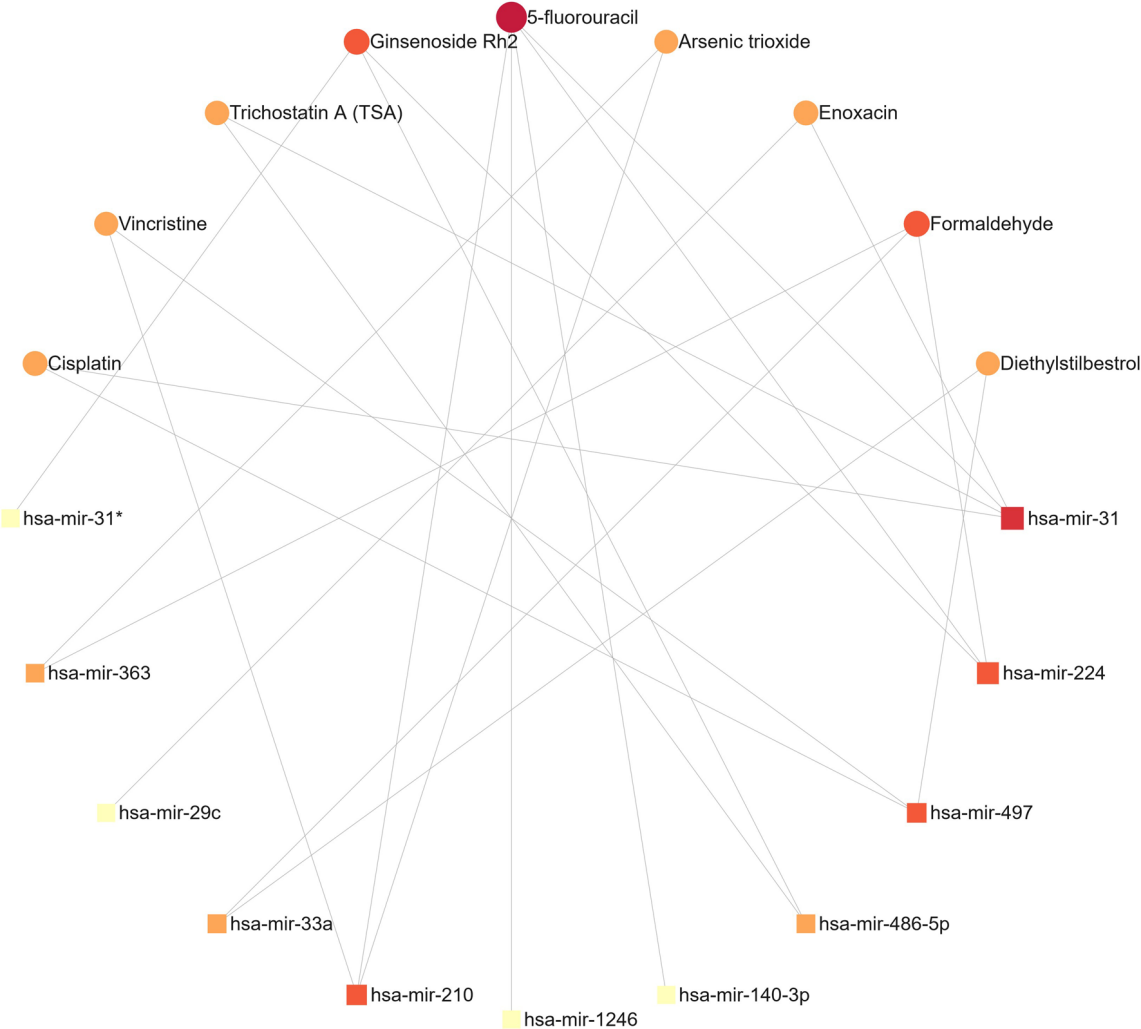
The minimum network of Co-DEmiRNA-Compound. In the optimal network, 5-fluorouracil, Arsenic trioxide, Cisplatin, Diethylstilbestrol, and Enoxacin were the compounds most strongly associated with known Co-DEmiRNAs (block: miRNA, circle: small molecule compound. The darker the color and larger the size of the key node in the figure, the higher its degree)

The query obtained several disease-related small molecule compounds (5-fluorouracil, Enoxacin 2, Cisplatin, etc.). A Co-DEmiRNA-Compound minimum network was constructed, consisting of 9 compounds and 11 miRNAs with 23 edges (Fig. 7, Additional file 1: Table S5). The most relevant miRNAs are hsa-mir-31, has-mir-224, has-mir-210, and has-mir-497. The nine compounds in this network include Formaldehyde, Ginsenoside Rh2, Trichostatin A (TSA), and Vincristine. The compound with the highest degree and betweenness is 5-fluorouracil, followed by Ginsenoside Rh2, Formaldehyde.

### Key Co-DEmiRNAs/targets identification and diagnostic efficacy

**Fig. 8:**
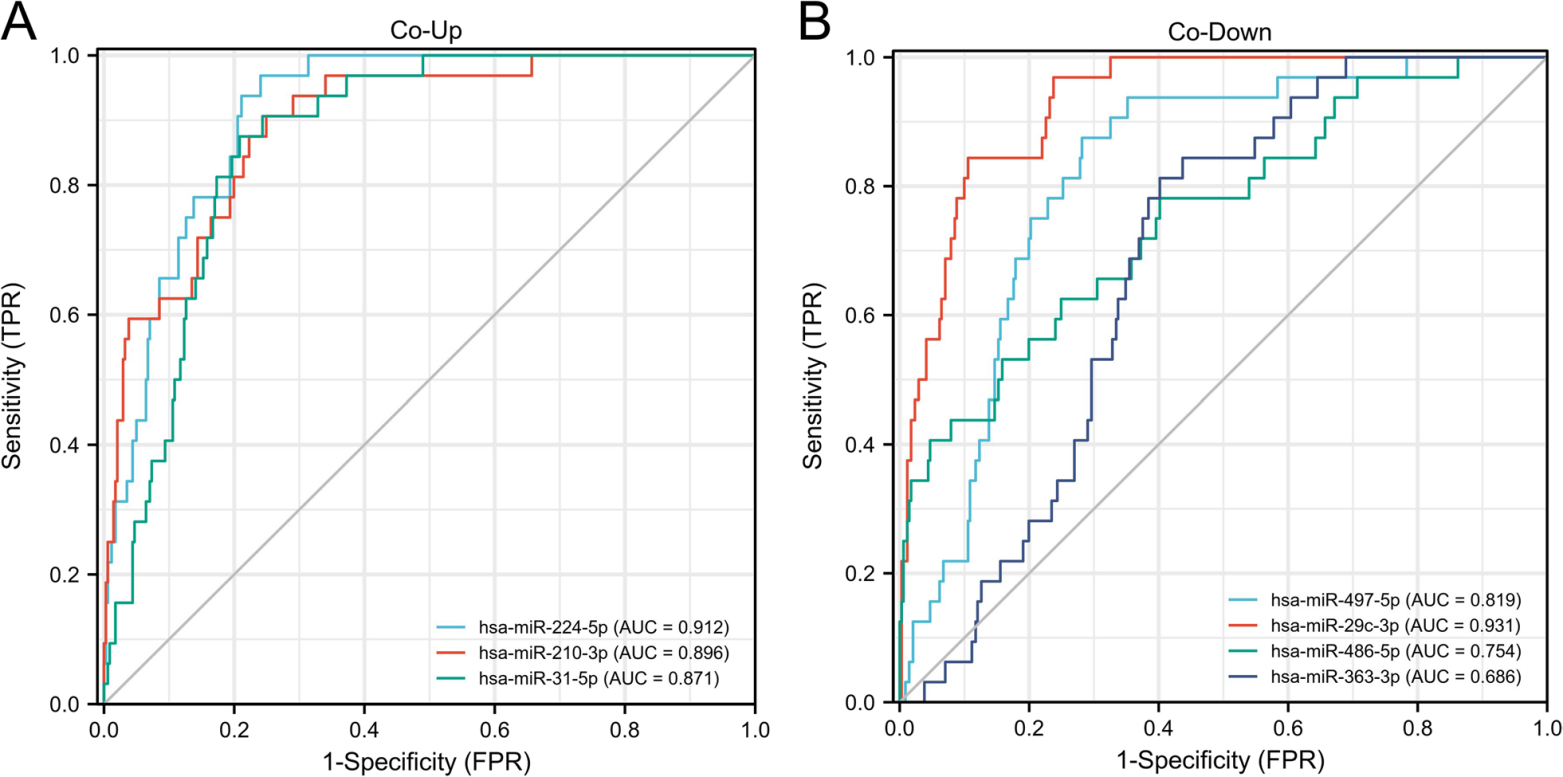
The ROC curve of top Co-DEmiRNAs. Co-DEmiRNAs, both co-up and co-down expressed, had a strong predictive ability for disease diagnosis in OSCC (AUC > 0.7, with larger values demonstrating that this miRNA has a strong predictive ability for the diagnosis of OSCC)

The key Co-DEmiRNAs and targets were sorted out in Table 5. We selected the data of OSCC in TGCA as a validation set. The diagnostic efficacy of the top Hub Co-DEmiRNAs in OSCC was examined. The receiver operating characteristic (ROC) curves of these Co-DEmiRNA are depicted (Fig. 8). The predictive ability of variable hsa-mir-29c (AUC = 0.931) showed high accuracy, and the predictive ability of variables hsa-mir-497 (AUC = 0.819) and hsa-mir-486-5p (AUC = 0.7548) showed certain accuracy. The predictive ability of variable hsa-mir-224-5p (AUC = 0.912) showed high accuracy, hsa-mir-31-5p (AUC = 0.871) and variable hsa-mir-210-3p (AUC = 0.896) showed certain accuracy.

**Table 5:** The Co-DEmiRNA-Associated Target Networks and Top Nodes in OSCC and PD

Networks	Top DEmiRNA		Top Gene/TF/Compound
	Co-Up ↑	Co-Down ↓	
Co-DEmiRNA-Gene	hsa-mir-224 hsa-mir-210	hsa-mir-497 hsa-mir-29c hsa-mir-486-5p	ZNF460, FBN1, CDK6, BTG2, CBX6
Co-DEmiRNA-TF	hsa-mir-224 hsa-mir-210 hsa-mir-31	hsa-mir-29c hsa-mir-363 hsa-mir-497	HIF1A, TP53, E2F1, MYCN, JUN
Co-DEmiRNA-Compound	hsa-mir-31 hsa-mir-224 hsa-mir-210	hsa-mir-497 hsa-mir-486-5p	5-fluorouracil, Ginsenoside Rh2, Formaldehyde, Cisplatin, Vincristine

## Discussion

The miRNA is an essential intermediate hub of host physiological and pathophysiological activities [33]. We know that the microbiota changes host miRNA using self-virulence factors, reducing the host immune response-ability, and achieving the final effect of pathogenicity [34–36]. Oral pathogens are important risk factors for periodontitis (PD) and oral squamous cell carcinoma (OSCC) [18, 37]. In recent years, it has been gradually discovered that PD-related pathogenic microorganisms, mainly *Porphyromonas gingivalis* (*P. gingivalis*) and *Fusobacterium nucleatum* (*F. nucleatum*), have played an essential role in oral cancer occurrence [38], which revealed to us that PD might also be the cause of OSCC or a key step in the malignant transformation process of oral disease. Overall, there may have a homologous genetic and molecular link between OSCC and PD.

Our current study explored the epigenetic mechanism of CO-DEmiRNA mediated the association between OSCC and PD by screening and identifying Co-DEmiRNA common in the two diseases. The network architecture was applied to determine DEmiRNA-related hub genes and TF, which could be used as the linkage mechanism of differential expression and further function of DEmiRNA. In addition, functional enrichment analysis was conducted on them to determine key pathways, molecular functions, and cell components. In addition, the small molecule compounds associated with Co-DEmiRNA were analyzed, and the key junction compounds between OSCC and PD were explored. The key Co-DEmiRNAs identified in this study may provide more effective guidance in the future study of inflammation-cancer transformation.

Most DEmiRNAs had the same expression trend in the two diseases, which further revealed the similar immune mechanism of the host oral microenvironment against inflammation or cancer, perhaps a common pattern of miRNA dysregulation in pro-inflammatory and pro-cancer responses. Co-DEmiRNAs with the highest degree included hsa-mir-224, hsa-mir-210, hsa-mir-31(overexpressed), and hsa-mir-497, hsa-mir-29c, hsa-mir-486(which were low expressed). They are all broadly involved in inflammation, cancer, and host immune responses.hsa-mir-224 is considered an early diagnostic marker of cancer [22], and both it and hsa-mir-210 are significantly involved in cancer progression and metastasis [23]. hsa-mir-31 is an important protective factor of the epithelial barrier [24] and has also been recognized as a cancer biomarker [25, 26]. hsa-mir-497 and has-mir-29c suppress various cancers, inhibiting the proliferation and growth of cancer [27–30]. hsa-mir-486 is a migration suppressor of various tumors and plays an important role in regulating epithelial-mesenchymal transition (EMT) [31, 32].

Our study revealed that dysregulation of associated gene expression mediated by noncoding RNA represented by miRNA might be the key mechanism linking PD to OSCC or other cancers. The genes with the highest degree in the Co-DEmiRNA-Gene network include *ZNF460*, *FBN1*, *CDK6*, *BTG2,* and *CBX6*, which may be the essential hub genes/mediators between OSCC and PD. *ZNF460* (zinc finger protein 460) is involved in the regulation of multiple cancer processes by JAK2/STAT3 pathway [39], and its high expression is associated with the proliferation, invasion, and metastasis of colorectal cancer and oral cancer [39, 40]. *FBN1* (fibrinin-1) is a common extracellular matrix encoding gene [41], and inactivation will affect the integrity of tissues (aortic wall, periodontal membrane, oral epithelial barrier, etc.). It encodes the formation of Oxytalan fibers [42], a unique component of the periodontal ligament (PDL). Low expression of *FBN1* inhibits TGF-β 1-mediated expression of Periosteum, thereby inhibiting collagen fiber production. In addition, *FBN1* also plays an important role in the Wnt/β-catenin signaling pathway that regulates cancer cell migration [43]. *CDK6* (cyclin-dependent kinase 6), as one of the proto-oncogenes driving tumors, has become a key target of various cancer therapies [44], and its inhibition can significantly affect tumor cell metabolism and antitumor immunity [45, 46]. *CDK6* also inhibits the proliferation of periodontal ligament cells (PDLCs) by regulating the cell cycle in periodontitis [47]. *BTG2* (B cell translocation gene 2) has long been recognized as a tumor suppressor gene in various cellular processes [48–50], including cell division, DNA repair, transcriptional regulation, and messenger RNA stability. Upregulation of *BTG2* inhibits cancer migration, invasion, EMT and, glycolysis [51]. *CBX6* (chromobox protein 6) accelerates EMT in head and neck squamous cell carcinoma [52], resulting in cancer progression.

In the Co-DEmiRNA-TF network, transcription factors HIF1A, TP53, E2F1, MYCN, and JUN have the highest degree. HIF1A (hypoxia-induced transcription factor 1α) can promote gingival tissue aging and hypoxia stress [53], regulate apoptosis of PDLCs [54] and increase the severity of periodontal inflammation [55]. Inhibits the expression of PPP1R1B and subsequent degradation of the p53 protein in pancreatic cancer cells [56]. Loss of HIF1A can also increase cancer cell proliferation, invasion, and metastasis activity [57]. Transcription factor P53 (tumor protein 53) controls the cell cycle, apoptosis, and cell senescence of periodontal ligament fibroblasts in periodontitis [58]. It plays an important role as a star transcription factor in oral squamous cell carcinoma [59]. Its protein level and phosphorylated protein levels are important factors in suppressing cancer. Low levels of p53 are directly related to the incidence and poor prognosis of oral squamous cell carcinoma [60]. E2F1 (recombinant E2F transcription factor 1) is related to changes in cell metabolism, cell–matrix interaction, and cell cycle [61], and it plays a crucial role in the NF-κB pathway in infection, inflammation and carcinogenesis [62], which can inhibit cell proliferation, migration, invasion and EMT processes. MYCN (N-Myc proto-oncogene protein) is a key marker for cell survival and a key transcription factor for maintaining the homeostasis of the periodontal epithelial barrier and inhibiting periodontal inflammation [63]. Its low expression can promote antiapoptotic resistance and EMT [64]. MYCN is associated with the Wnt/β-catenin pathway in OSCC tumorigenesis and inhibits epithelial-mesenchymal transformation, migration, and colony formation in OSCC. JUN (JUN proto-oncogene protein, AP-1 transcription factor) is related to immune infiltration [65], which causes inflammation and cell death through immunosuppression, leading to cancer.

Functional enrichment analysis of Co-DEmiRNA-Gene, Hub genes and TF networks showed that many cancer-related KEGG/Reactome pathways are enriched, supporting previous findings that PD is a significant risk factor for OSCC (Like PI3 K-related signaling pathway and MAPK pathway). Ras protein signal transduction and the functional enrichment of transcription factor binding in GO analysis are very obvious. These play a crucial role in inflammation, immunosuppression, and antitumor immunity [66–69].

In this study, the compounds most closely related to Co-DEmiRNA of the two diseases were also analyzed. 5-fluorouracil(5-FU), Ginsenoside, Rh2, and Formaldehyde are the small molecule compounds with the strongest correlation with Co-DEmiRNA. miRNAs reduce the resistance of oral squamous cell carcinoma cells to 5-fluorouracil [70]. At the same time, 5-FU also increases the severity and duration of periodontitis and damages tissue repair by reducing cell and blood vessel renewal, leading to more severe periodontal damage [71]. Ginsenoside Rh2 can control inflammation by regulating the STAT3 signaling pathway and NF-κB signaling pathway to reduce the production of inflammatory factors at mucosal sites [72, 73]. At the same time, it can also inhibit tumor invasion, migration, and angiogenesis by regulating miRNA or AMPK/mTOR and other signaling pathways [74, 75], and induce cancer cell apoptosis and protective autophagy[76]. Formaldehyde is a typical risk factor, which can cause oxidative damage, inflammation, and genotoxicity, and greatly increase the risk of cancer [77, 78]. Future studies will be necessary to investigate these rich compounds in the context of OSCC and PD association.

This study investigated the epigenetic mechanism linked between OSCC and PD, including multiple aspects, such as DEmiRNA, Co-DEmiRNA, Hub gene, TF, and even related compounds. The main limitation is the lack of further experimental data to validate these candidate key linking mechanisms. The datasets used in this study were from a single database, which may limit the accuracy of the results. Future-related research using diverse composite data is critical and necessary. Another point is that other noncoding RNAs, such as lncRNAs, circRNAs, and sncRNAs, may also play an important role in the pathogenic mechanism of OSCC and PD, which were not investigated in this study. Therefore, future studies may further investigate other noncoding RNAs as linkage mechanisms. Future studies should aim to validate the further link between Co-DEmiRNA Hub genes, TF pathway, and compound, these key parts between OSCC and PD, using clinical studies, in vitro and in vivo experiments, etc. In addition, since this association may be bidirectional, it is necessary to comprehensively study the biological mechanisms involved, which will also provide a basis for us to explain the inflammation-cancer transformation further.

## Conclusions

Comprehensive analysis of Co-DEmiRNAs in OSCC and PD revealed key genetic molecular mechanisms (Table 5), including miRNAs (including hsa-mir-224, hsa-mir-210, hsa-mir-497, hsa-mir-29c, hsa-mir-486-5p and hsa-mir-31), genes (including *ZNF460*, *FBN1*, *CDK6*, *BTG2*, *CBX6*) and TFs (including HIF1A, TP53, E2F1, MYCN, and JUN). It highlighted the most DEmiRNA-related small molecule compounds (including 5-fluorouracil, Ginsenoside Rh2, Formaldehyde and Cisplatin, and Vincristine). These findings provide a theoretical basis to guide future experimental research.

## Supplementary Information

**Additional file 1. Table S1a.** Differentially Expressed miRNA in Oral Squamous Cell Carcinoma (GSE45238). **Table S1b.** Differentially expressed miRNA in Periodontitis (GSE54710). **Table S2a.** Co-DEmiRNA-Gene Minimum Network. **Table S2b.** KEGG Pathway Enrichment Analysis of Co-DEmiRNA-Gene Network. **Table S2c.** Reactome Pathway Enrichment Analysis of Co-DEmiRNA-Gene Network. **Table S2d.** Gene Ontology-Biological Process Enrichment Analysis of Co-DEmiRNA-Gene Network. **Table S2e.** Gene Ontology-Molecular Functions Enrichment Analysis of Co-DEmiRNA-Gene Network. **Table S2f.** Gene Ontology-Cellular Component Enrichment Analysis of Co-DEmiRNA-Gene Network. **Table S3.** The Transcription Factors of 11 Co-DEmiRNA. **Table S4a.** Co-DEmiRNA-TF Minimum Network. **Table S4b.** KEGG Pathway Enrichment Analysis of Co-DEmiRNA-TF Network. **Table S4c.** Reactome Pathway Enrichment Analysis of Co-DEmiRNA-TF Network. **Table S4d.** Gene Ontology-Biological Process Enrichment Analysis of Co-DEmiRNA-TF Network. **Table S4e.** Gene Ontology-Molecular Functions Enrichment Analysis of Co-DEmiRNA-TF Network. **Table S4f.** Gene Ontology-Cellular Component Enrichment Analysis of Co-DEmiRNA-TF Network. **Table S5.** Co-DEmiRNA-Compound Minimum Network.

## Abbreviations

OSCC: Oral squamous cell carcinoma
PD: Periodontitis
DEmiRNA: Differentially expressed miRNA
Co-DEmiRNA: Common (co-over expression/co-low expression) DEmiRNA
TF: Transcription factor
GO: Gene ontology
CC: Cellular component
MF: Molecular function
BP: Biological process
EMT: Epithelial-mesenchymal transition

## Declarations

### Availability of data and materials

The datasets analyzed during the current study are available in the GEO repository. (https://www.ncbi.nlm.nih.gov/geo/).
