# Supplementary material for: Network analysis reveals miRNA crosstalk between periodontitis and oral squamous cell carcinoma
Source: BMC Oral Health. 2023 Jan 13;23:19. doi: 10.1186/s12903-022-02704-2 (PMC9840318; doi:10.1186/s12903-022-02704-2)
Supplement: Supplementary file 1 — Additional file 1. Table S1a. Differentially Expressed miRNA in Oral Squamous Cell Carcinoma (GSE45238). Table S1b. Differentially expressed miRNA in Periodontitis (GSE54710). Table S2a. Co-DEmiRNA-Gene Minimum Network. Table S2b. KEGG Pathway Enrichment Analysis of Co-DEmiRNA-Gene Network. Table S2c. Reactome Pathway Enrichment Analysis of Co-DEmiRNA-Gene Network. Table S2d. Gene Ontology-Biological Process Enrichment Analysis of Co-DEmiRNA-Gene Network. Table S2e. Gene Ontology-Molecular Functions Enrichment Analysis of Co-DEmiRNA-Gene Network. Table S2f. Gene Ontology-Cellular Component Enrichment Analysis of Co-DEmiRNA-Gene Network. Table S3. The Transcription Factors of 11 Co-DEmiRNA. Table S4a. Co-DEmiRNA-TF Minimum Network. Table S4b. KEGG Pathway Enrichment Analysis of Co-DEmiRNA-TF Network. Table S4c. Reactome Pathway Enrichment Analysis of Co-DEmiRNA-TF Network. Table S4d. Gene Ontology-Biological Process Enrichment Analysis of Co-DEmiRNA-TF Network. Table S4e. Gene Ontology-Molecular Functions Enrichment Analysis of Co-DEmiRNA-TF Network. Table S4f. Gene Ontology-Cellular Component Enrichment Analysis of Co-DEmiRNA-TF Network. Table S5. Co-DEmiRNA-Compound Minimum Network. [file 12903_2022_2704_MOESM1_ESM.docx]

Supplementary Material

**Annotation**

OSCC: Oral Squamous Cell Carcinoma.

LogFC: Log Fold Change, adj.P.Val:Adjusted P value.

GO:Gene Ontology, CC:Cellular Component, MF:Molecular Function, BP:Biological Process.

DEmiRNA: Differentially Expressed miRNA, TF:Transcription Factor

Co-DEmiRNA:Common (co-over expression/co-low expression)-DEmiRNA

**Table S1a: Differentially Expressed miRNA in Oral Squamous Cell Carcinoma (GSE45238).**

| ID | miRNA_ID | P.Value | logFC | adj.P.Val | t | B |
| --- | --- | --- | --- | --- | --- | --- |
| ILMN_3168646 | hsa-miR-21* | 1.26E-26 | 2.6762247 | 1.08E-23 | 15.840144 | 50.1057 |
| ILMN_3168513 | hsa-let-7c | 2.63E-23 | -1.5677145 | 1.13E-20 | -13.941487 | 42.5755 |
| ILMN_3167455 | hsa-miR-30a | 1.11E-21 | -1.640939 | 3.05E-19 | -13.051243 | 38.8445 |
| ILMN_3168273 | hsa-miR-503 | 1.42E-21 | 2.3655521 | 3.05E-19 | 12.99221 | 38.66 |
| ILMN_3166992 | hsa-miR-139-5p | 2.68E-20 | -1.8184499 | 4.60E-18 | -12.309486 | 35.7566 |
| ILMN_3168388 | hsa-miR-7 | 8.65E-20 | 1.8411588 | 1.24E-17 | 12.040244 | 34.5746 |
| ILMN_3168830 | hsa-miR-29c* | 4.39E-19 | -1.3825851 | 5.38E-17 | -11.669949 | 32.9829 |
| ILMN_3168749 | hsa-miR-455-3p | 7.06E-18 | 1.3757466 | 6.73E-16 | 11.043944 | 30.1059 |
| ILMN_3166938 | hsa-miR-424 | 1.97E-17 | 1.5520405 | 1.69E-15 | 10.814219 | 29.0435 |
| ILMN_3167988 | hsa-miR-411 | 2.53E-17 | -2.4518887 | 1.97E-15 | -10.758673 | 28.9933 |
| ILMN_3168213 | hsa-miR-99a | 3.46E-17 | -1.4022186 | 2.48E-15 | -10.688762 | 28.4438 |
| ILMN_3168638 | hsa-miR-424* | 2.00E-16 | 1.6258437 | 1.32E-14 | 10.299842 | 26.9218 |
| ILMN_3168692 | hsa-miR-1267 | 2.84E-16 | 1.5547842 | 1.74E-14 | 10.222047 | 26.6033 |
| ILMN_3168707 | hsa-miR-921 | 3.15E-16 | -0.8456498 | 1.80E-14 | -10.199327 | 26.4177 |
| ILMN_3167714 | hsa-miR-455-5p | 3.46E-16 | 1.4009426 | 1.86E-14 | 10.178436 | 26.4011 |
| ILMN_3168389 | hsa-miR-125b | 3.77E-16 | -0.7123212 | 1.90E-14 | -10.159575 | 25.7851 |
| ILMN_3167711 | hsa-miR-30e* | 1.30E-15 | -0.5948298 | 6.21E-14 | -9.886288 | 24.8771 |
| ILMN_3168813 | hsa-miR-885-5p | 1.57E-15 | -1.8980644 | 7.10E-14 | -9.845014 | 24.8479 |
| ILMN_3167823 | hsa-miR-135a | 2.07E-15 | -2.248729 | 8.89E-14 | -9.784484 | 24.6369 |
| ILMN_3167805 | hsa-miR-487b | 2.98E-15 | -2.0869986 | 1.22E-13 | -9.704472 | 24.2927 |
| ILMN_3167325 | hsa-miR-34b* | 5.75E-15 | 2.0798246 | 2.24E-13 | 9.560223 | 23.6372 |
| ILMN_3167158 | hsa-miR-30a* | 6.23E-15 | -2.3009585 | 2.33E-13 | -9.542548 | 23.5641 |
| ILMN_3167229 | hsa-miR-375 | 6.89E-15 | -4.0135763 | 2.46E-13 | -9.52071 | 23.414 |
| ILMN_3167913 | hsa-miR-299-5p | 8.02E-15 | -1.9381313 | 2.75E-13 | -9.487468 | 23.3215 |
| ILMN_3168765 | hsa-miR-663b | 1.82E-14 | 1.604606 | 6.00E-13 | 9.307936 | 22.5051 |
| ILMN_3167634 | hsa-miR-100 | 6.47E-14 | -0.8711389 | 2.06E-12 | -9.030181 | 20.8856 |
| ILMN_3167624 | hsa-miR-136 | 7.83E-14 | -1.9030819 | 2.40E-12 | -8.988622 | 21.0663 |
| ILMN_3167522 | hsa-miR-154 | 1.09E-13 | -1.9787871 | 3.22E-12 | -8.916336 | 20.7454 |
| ILMN_3167818 | hsa-miR-432 | 1.30E-13 | -1.6166699 | 3.72E-12 | -8.877504 | 20.5798 |
| ILMN_3168180 | hsa-miR-378* | 2.27E-13 | -1.8195046 | 6.18E-12 | -8.755469 | 20.0072 |
| ILMN_3167240 | hsa-miR-486-5p | 2.31E-13 | -1.8538086 | 6.18E-12 | -8.752283 | 19.8785 |
| ILMN_3168716 | hsa-miR-337-3p | 4.40E-13 | -2.4568625 | 1.14E-11 | -8.610947 | 19.3591 |
| ILMN_3166941 | hsa-miR-376c | 5.37E-13 | -1.5429432 | 1.36E-11 | -8.567277 | 19.0696 |
| ILMN_3167191 | hsa-miR-195 | 6.05E-13 | -0.7778017 | 1.48E-11 | -8.54134 | 18.664 |
| ILMN_3168187 | hsa-miR-450a | 1.16E-12 | 1.2202189 | 2.77E-11 | 8.398384 | 18.4136 |
| ILMN_3167344 | hsa-miR-338-3p | 1.41E-12 | -1.3423453 | 3.27E-11 | -8.356255 | 18.1421 |
| ILMN_3167642 | hsa-miR-499-5p | 1.59E-12 | -1.8156043 | 3.55E-11 | -8.329485 | 17.9683 |
| ILMN_3167743 | hsa-miR-34c-5p | 1.61E-12 | 1.5733594 | 3.55E-11 | 8.326708 | 18.0988 |
| ILMN_3167175 | hsa-miR-542-5p | 1.66E-12 | 1.7848092 | 3.57E-11 | 8.319783 | 17.9984 |
| ILMN_3166971 | hsa-miR-95 | 2.21E-12 | 0.6746457 | 4.63E-11 | 8.257328 | 17.2681 |
| ILMN_3167628 | hsa-miR-196a | 3.66E-12 | 3.0438055 | 7.48E-11 | 8.146984 | 17.2563 |
| ILMN_3167210 | hsa-miR-502-5p | 3.76E-12 | -1.3261735 | 7.49E-11 | -8.141354 | 17.1869 |
| ILMN_3167443 | hsa-miR-379 | 4.40E-12 | -1.7889422 | 8.39E-11 | -8.106635 | 17.0958 |
| ILMN_3168396 | hsa-miR-671:9.1 | 9.54E-12 | 1.048194 | 1.78E-10 | 7.936753 | 16.2836 |
| ILMN_3168697 | hsa-miR-1268 | 1.08E-11 | 1.4696972 | 1.96E-10 | 7.910307 | 16.2334 |
| ILMN_3168667 | hsa-miR-136* | 1.81E-11 | -1.3209363 | 3.23E-10 | -7.796307 | 15.6877 |
| ILMN_3168851 | hsa-miR-1290 | 1.85E-11 | 1.5987085 | 3.23E-10 | 7.791476 | 15.6051 |
| ILMN_3167244 | hsa-miR-410 | 4.89E-11 | -1.7677775 | 8.39E-10 | -7.576343 | 14.642 |
| ILMN_3168354 | hsa-miR-519d | 7.97E-11 | 0.8925192 | 1.34E-09 | 7.468422 | 13.7919 |
| ILMN_3168282 | hsa-miR-18a | 8.24E-11 | 1.4148546 | 1.36E-09 | 7.460946 | 14.1436 |
| ILMN_3168380 | hsa-miR-218 | 1.01E-10 | -1.5539512 | 1.63E-09 | -7.416776 | 14.0289 |
| ILMN_3167837 | hsa-miR-31 | 1.05E-10 | 1.4787121 | 1.66E-09 | 7.407618 | 13.4966 |
| ILMN_3167031 | hsa-miR-127-3p | 2.01E-10 | -1.614127 | 3.04E-09 | -7.262836 | 13.2757 |
| ILMN_3167977 | hsa-miR-498 | 2.02E-10 | 1.7554619 | 3.04E-09 | 7.261836 | 13.2374 |
| ILMN_3168847 | hsa-miR-31* | 1.99E-10 | 2.3512737 | 3.04E-09 | 7.264865 | 13.2338 |
| ILMN_3168772 | hsa-miR-1226* | 2.10E-10 | 0.5780261 | 3.10E-09 | 7.253489 | 12.863 |
| ILMN_3167894 | hsa-miR-146b-5p | 2.16E-10 | 1.1209902 | 3.14E-09 | 7.247139 | 13.0251 |
| ILMN_3167843 | hsa-miR-768-5p:11.0 | 2.42E-10 | -0.9229034 | 3.44E-09 | -7.221567 | 12.776 |
| ILMN_3168553 | hsa-miR-362-3p | 2.75E-10 | 0.8096282 | 3.80E-09 | 7.193197 | 13.0299 |
| ILMN_3167052 | hsa-miR-495 | 2.87E-10 | -1.6459022 | 3.91E-09 | -7.18314 | 12.9879 |
| ILMN_3167006 | hsa-miR-378 | 3.54E-10 | -0.7941068 | 4.75E-09 | -7.136412 | 12.3506 |
| ILMN_3168609 | hsa-miR-25* | 3.96E-10 | 0.886023 | 5.23E-09 | 7.1116 | 12.6391 |
| ILMN_3168544 | hsa-miR-106a | 4.15E-10 | 0.5936093 | 5.40E-09 | 7.101018 | 12.2401 |
| ILMN_3168746 | hsa-miR-1272 | 4.56E-10 | -0.6184166 | 5.84E-09 | -7.079967 | 12.4557 |
| ILMN_3167643 | hsa-miR-29c | 5.25E-10 | -0.6874929 | 6.62E-09 | -7.048638 | 11.8703 |
| ILMN_3168571 | hsa-miR-1246 | 8.88E-10 | 0.6647345 | 1.09E-08 | 6.930558 | 11.2261 |
| ILMN_3168804 | hsa-miR-125b-2* | 1.19E-09 | -1.3428551 | 1.42E-08 | -6.864368 | 11.5732 |
| ILMN_3168698 | hsa-miR-486-3p | 2.50E-09 | -1.5005591 | 2.86E-08 | -6.696874 | 10.8563 |
| ILMN_3167944 | hsa-miR-193b | 2.54E-09 | 0.5794349 | 2.87E-08 | 6.693621 | 10.3833 |
| ILMN_3168308 | hsa-miR-196b | 4.54E-09 | 0.8870584 | 4.99E-08 | 6.561626 | 10.3069 |
| ILMN_3168822 | hsa-miR-1204 | 6.50E-09 | 0.5718952 | 6.97E-08 | 6.479664 | 9.9359 |
| ILMN_3168361 | hsa-miR-181a* | 6.61E-09 | 0.6281615 | 7.00E-08 | 6.475969 | 9.9363 |
| ILMN_3168477 | hsa-miR-381 | 6.86E-09 | -1.1935333 | 7.17E-08 | -6.467488 | 9.8256 |
| ILMN_3167606 | hsa-miR-801:9.1 | 9.41E-09 | 0.8854451 | 9.61E-08 | 6.394851 | 9.5612 |
| ILMN_3168509 | hsa-miR-142-5p | 1.03E-08 | 1.7437136 | 1.04E-07 | 6.373461 | 9.4401 |
| ILMN_3167178 | hsa-miR-18a* | 1.21E-08 | 1.259784 | 1.20E-07 | 6.336547 | 9.3387 |
| ILMN_3168314 | hsa-miR-133a | 1.51E-08 | -2.0419656 | 1.47E-07 | -6.285853 | 8.6353 |
| ILMN_3168198 | hsa-miR-328 | 1.66E-08 | -1.2625972 | 1.59E-07 | -6.263641 | 9.029 |
| ILMN_3168630 | hsa-miR-628-5p | 1.68E-08 | -0.9754698 | 1.59E-07 | -6.261789 | 9.0273 |
| ILMN_3168786 | hsa-miR-140-3p | 1.68E-08 | -0.5701662 | 1.59E-07 | -6.261015 | 8.675 |
| ILMN_3168118 | hsa-miR-1228* | 1.71E-08 | 0.863752 | 1.60E-07 | 6.257001 | 8.8027 |
| ILMN_3166979 | hsa-miR-223 | 1.82E-08 | 0.7500933 | 1.68E-07 | 6.243398 | 8.1547 |
| ILMN_3168320 | hsa-miR-1 | 1.96E-08 | -3.2505816 | 1.77E-07 | -6.226458 | 8.7456 |
| ILMN_3168655 | hsa-miR-32* | 1.96E-08 | 0.6989797 | 1.77E-07 | 6.225663 | 8.4776 |
| ILMN_3167215 | hsa-miR-370 | 2.32E-08 | -1.1353385 | 2.07E-07 | -6.186667 | 8.7062 |
| ILMN_3168551 | hsa-miR-944 | 2.34E-08 | 1.3780181 | 2.07E-07 | 6.185171 | 8.6959 |
| ILMN_3167105 | hsa-miR-208b | 2.61E-08 | -1.5614127 | 2.28E-07 | -6.159953 | 8.4977 |
| ILMN_3167685 | hsa-miR-139-3p | 2.84E-08 | -1.0720948 | 2.46E-07 | -6.140023 | 8.3778 |
| ILMN_3168757 | hsa-miR-145* | 4.30E-08 | -0.9808908 | 3.69E-07 | -6.043466 | 8.0189 |
| ILMN_3167239 | hsa-miR-382 | 7.28E-08 | -0.8863725 | 6.12E-07 | -5.91998 | 7.5922 |
| ILMN_3168147 | hsa-miR-520h,hsa-miR-520g | 7.75E-08 | 0.9546014 | 6.45E-07 | 5.905413 | 7.0773 |
| ILMN_3167412 | hsa-miR-628-3p | 8.01E-08 | -1.6690684 | 6.49E-07 | -5.897436 | 7.4678 |
| ILMN_3168127 | hsa-miR-655 | 7.88E-08 | -1.4201832 | 6.49E-07 | -5.901225 | 7.3933 |
| ILMN_3167787 | hsa-miR-19a | 8.32E-08 | 1.6343793 | 6.67E-07 | 5.888608 | 7.355 |
| ILMN_3167963 | hsa-miR-222 | 8.78E-08 | 0.5650676 | 6.98E-07 | 5.875872 | 6.8562 |
| ILMN_3168301 | hsa-miR-339-5p | 9.91E-08 | 0.6058272 | 7.80E-07 | 5.847249 | 7.2055 |
| ILMN_3168738 | hsa-miR-1271 | 1.03E-07 | -0.9068454 | 7.94E-07 | -5.838738 | 7.2576 |
| ILMN_3167382 | hsa-miR-565:9.1 | 1.04E-07 | 0.6699473 | 7.99E-07 | 5.835291 | 6.724 |
| ILMN_3167353 | hsa-miR-107 | 1.26E-07 | -0.5967857 | 9.55E-07 | -5.790831 | 7.0178 |
| ILMN_3168568 | hsa-miR-34c-3p | 1.43E-07 | 0.9573931 | 1.07E-06 | 5.760047 | 6.6696 |
| ILMN_3167437 | hsa-miR-497 | 2.33E-07 | -0.8713673 | 1.72E-06 | -5.644073 | 6.235 |
| ILMN_3167074 | hsa-miR-542-3p | 2.93E-07 | 0.7968153 | 2.13E-06 | 5.589401 | 6.2377 |
| ILMN_3168170 | hsa-miR-155 | 2.92E-07 | 0.9231136 | 2.13E-06 | 5.589658 | 5.9919 |
| ILMN_3167529 | hsa-miR-183 | 3.22E-07 | 0.8417133 | 2.32E-06 | 5.566719 | 6.0824 |
| ILMN_3167151 | hsa-miR-553 | 3.49E-07 | 0.5337145 | 2.48E-06 | 5.546784 | 5.7487 |
| ILMN_3168563 | hsa-miR-708 | 5.28E-07 | 0.6808642 | 3.68E-06 | 5.44699 | 5.3793 |
| ILMN_3166955 | hsa-miR-369-5p | 5.83E-07 | -0.5592951 | 4.03E-06 | -5.423024 | 5.5437 |
| ILMN_3168668 | hsa-miR-200b* | 6.63E-07 | -0.9139655 | 4.55E-06 | -5.391445 | 5.3605 |
| ILMN_3168731 | hsa-miR-29b-2* | 6.84E-07 | -0.9288162 | 4.66E-06 | -5.384001 | 5.3429 |
| ILMN_3167260 | hsa-miR-19b | 1.27E-06 | 1.1691993 | 8.37E-06 | 5.232572 | 4.4328 |
| ILMN_3168750 | hsa-miR-1308 | 1.34E-06 | 0.5249405 | 8.68E-06 | 5.219678 | 3.9122 |
| ILMN_3167807 | hsa-miR-661 | 1.52E-06 | 0.9254568 | 9.80E-06 | 5.187784 | 4.5848 |
| ILMN_3168706 | hsa-miR-331-5p | 1.73E-06 | 0.7576236 | 1.10E-05 | 5.155348 | 4.3465 |
| ILMN_3168097 | hsa-miR-658 | 1.91E-06 | 0.7427899 | 1.20E-05 | 5.131473 | 3.9469 |
| ILMN_3167611 | hsa-miR-331-3p | 2.02E-06 | 0.5254866 | 1.27E-05 | 5.11634 | 4.0823 |
| ILMN_3168178 | hsa-miR-493 | 2.18E-06 | -1.4473764 | 1.35E-05 | -5.098132 | 4.2478 |
| ILMN_3168111 | hsa-miR-376a | 2.75E-06 | -1.2550712 | 1.70E-05 | -5.040037 | 4.0523 |
| ILMN_3167521 | hsa-miR-560:9.1 | 2.83E-06 | 1.0031827 | 1.74E-05 | 5.032148 | 3.9245 |
| ILMN_3167046 | hsa-miR-214 | 3.05E-06 | -0.6066948 | 1.85E-05 | -5.013931 | 3.3454 |
| ILMN_3167991 | hsa-miR-889 | 3.20E-06 | 0.6065146 | 1.93E-05 | 5.001981 | 3.9236 |
| ILMN_3167874 | hsa-miR-135b | 3.35E-06 | 1.2806606 | 1.98E-05 | 4.990408 | 3.8157 |
| ILMN_3167569 | hsa-miR-421 | 3.54E-06 | 0.7684699 | 2.08E-05 | 4.976412 | 3.8169 |
| ILMN_3168085 | hsa-miR-128a:9.1 | 3.98E-06 | -1.1981435 | 2.32E-05 | -4.94674 | 3.6894 |
| ILMN_3168742 | hsa-miR-337-5p | 4.73E-06 | -0.5680525 | 2.68E-05 | -4.902744 | 3.5112 |
| ILMN_3168884 | hsa-miR-450b-5p | 4.72E-06 | 0.7392177 | 2.68E-05 | 4.903386 | 3.5326 |
| ILMN_3168693 | hsa-miR-15b* | 5.14E-06 | 0.7090478 | 2.88E-05 | 4.881767 | 3.4567 |
| ILMN_3168212 | hsa-miR-132 | 5.67E-06 | 0.5911936 | 3.12E-05 | 4.856485 | 3.188 |
| ILMN_3168875 | hsa-miR-1300 | 5.88E-06 | 0.8768117 | 3.19E-05 | 4.84733 | 3.2787 |
| ILMN_3168644 | hsa-miR-7-1* | 6.03E-06 | 0.9220738 | 3.25E-05 | 4.840908 | 3.0777 |
| ILMN_3168724 | hsa-let-7i* | 6.96E-06 | 0.9565878 | 3.73E-05 | 4.804116 | 3.1644 |
| ILMN_3168800 | hsa-miR-379* | 7.27E-06 | -0.8846138 | 3.83E-05 | -4.792855 | 2.7936 |
| ILMN_3167472 | hsa-miR-32 | 7.23E-06 | 1.0529613 | 3.83E-05 | 4.794255 | 3.0627 |
| ILMN_3168634 | hsa-miR-218-1* | 7.91E-06 | -0.553758 | 4.14E-05 | -4.77104 | 3.0135 |
| ILMN_3167656 | hsa-miR-574-3p | 8.12E-06 | -0.5527037 | 4.20E-05 | -4.764347 | 2.6814 |
| ILMN_3168281 | hsa-miR-941 | 8.72E-06 | 1.0835841 | 4.48E-05 | 4.745764 | 2.8322 |
| ILMN_3168578 | hsa-miR-1247 | 9.44E-06 | -1.5299104 | 4.82E-05 | -4.725438 | 2.8566 |
| ILMN_3167099 | hsa-miR-130b | 9.85E-06 | 0.6008493 | 5.00E-05 | 4.714297 | 2.8085 |
| ILMN_3168348 | hsa-miR-133b | 1.18E-05 | -2.1554279 | 5.96E-05 | -4.667116 | 2.4906 |
| ILMN_3168257 | hsa-miR-181b | 1.23E-05 | 0.500274 | 6.19E-05 | 4.655936 | 2.4091 |
| ILMN_3168681 | hsa-miR-106b* | 1.31E-05 | 0.8728863 | 6.51E-05 | 4.641138 | 2.5654 |
| ILMN_3168025 | hsa-miR-134 | 2.07E-05 | -1.0757257 | 1.03E-04 | -4.519916 | 2.1173 |
| ILMN_3168324 | hsa-miR-550* | 2.18E-05 | 0.9238391 | 1.08E-04 | 4.505879 | 2.0269 |
| ILMN_3167948 | hsa-miR-18b | 2.74E-05 | 1.0094092 | 1.32E-04 | 4.44476 | 1.8493 |
| ILMN_3168344 | hsa-miR-363 | 2.92E-05 | -0.8538051 | 1.38E-04 | -4.428378 | 1.7892 |
| ILMN_3168196 | hsa-miR-101* | 2.90E-05 | -1.0534639 | 1.38E-04 | -4.429678 | 1.7498 |
| ILMN_3168727 | hsa-miR-30c-2* | 3.11E-05 | -0.6378002 | 1.47E-04 | -4.411415 | 1.51 |
| ILMN_3166935 | hsa-miR-329 | 3.63E-05 | -0.9655988 | 1.70E-04 | -4.369792 | 1.5633 |
| ILMN_3168072 | hsa-miR-548d-5p | 3.66E-05 | -0.5014699 | 1.71E-04 | -4.36738 | 1.5717 |
| ILMN_3167219 | hsa-miR-891a | 3.69E-05 | -1.0250444 | 1.71E-04 | -4.365107 | 1.0985 |
| ILMN_3167363 | hsa-miR-211 | 4.18E-05 | -0.8465511 | 1.92E-04 | -4.331485 | 1.2037 |
| ILMN_3167458 | hsa-miR-369-3p | 4.58E-05 | -1.1230752 | 2.08E-04 | -4.306471 | 1.3558 |
| ILMN_3167902 | hsa-miR-149 | 4.79E-05 | -0.7282912 | 2.16E-04 | -4.294508 | 1.1852 |
| ILMN_3168648 | hsa-miR-99a* | 6.33E-05 | -0.6885821 | 2.82E-04 | -4.218072 | 0.9818 |
| ILMN_3168425 | hsa-miR-598 | 6.94E-05 | -0.7296695 | 3.05E-04 | -4.192872 | 0.9631 |
| ILMN_3168587 | hsa-miR-1307 | 6.92E-05 | 0.6010246 | 3.05E-04 | 4.193743 | 0.9167 |
| ILMN_3168781 | hsa-miR-124 | 7.35E-05 | 1.0372703 | 3.20E-04 | 4.177093 | 0.477 |
| ILMN_3167491 | hsa-miR-128b:9.1 | 7.73E-05 | -0.8245311 | 3.35E-04 | -4.163212 | 0.7142 |
| ILMN_3167524 | hsa-miR-650 | 7.78E-05 | 1.1510578 | 3.35E-04 | 4.16134 | 0.5593 |
| ILMN_3168652 | hsa-miR-34b | 8.79E-05 | 0.6676618 | 3.77E-04 | 4.127469 | 0.2301 |
| ILMN_3168565 | hsa-miR-1244 | 8.98E-05 | 0.6587657 | 3.83E-04 | 4.121437 | 0.2731 |
| ILMN_3168434 | hsa-miR-1468 | 9.27E-05 | -0.6363482 | 3.94E-04 | -4.112592 | -0.1427 |
| ILMN_3167136 | hsa-miR-140-5p | 9.74E-05 | -0.5622819 | 4.12E-04 | -4.098923 | 0.497 |
| ILMN_3168806 | hsa-miR-128 | 1.02E-04 | -0.5906251 | 4.29E-04 | -4.085969 | 0.3792 |
| ILMN_3168589 | hsa-miR-29a* | 1.05E-04 | -0.5349655 | 4.37E-04 | -4.078146 | 0.5452 |
| ILMN_3167774 | hsa-miR-210 | 1.06E-04 | 0.6503972 | 4.38E-04 | 4.075763 | 0.2674 |
| ILMN_3167030 | hsa-miR-548b-3p | 1.08E-04 | -0.5036118 | 4.46E-04 | -4.069749 | 0.2892 |
| ILMN_3167691 | hsa-miR-33a | 1.25E-04 | 1.4854745 | 5.05E-04 | 4.02918 | 0.3789 |
| ILMN_3168767 | hsa-miR-376a* | 1.46E-04 | -0.9658102 | 5.85E-04 | -3.985219 | 0.0536 |
| ILMN_3168637 | hsa-miR-1826 | 2.74E-04 | 0.6571539 | 1.05E-03 | 3.804232 | -0.4315 |
| ILMN_3168745 | hsa-miR-1182 | 2.80E-04 | 0.6999458 | 1.06E-03 | 3.797638 | -0.6294 |
| ILMN_3168515 | hsa-miR-224 | 2.89E-04 | 0.6875783 | 1.09E-03 | 3.788048 | -0.827 |
| ILMN_3168363 | hsa-miR-509-3p | 2.99E-04 | -0.5692211 | 1.13E-03 | -3.778015 | -0.7115 |
| ILMN_3168774 | hsa-miR-1275 | 3.35E-04 | -0.6994138 | 1.24E-03 | -3.744565 | -0.5306 |
| ILMN_3167972 | hsa-miR-493* | 3.33E-04 | -0.5212558 | 1.24E-03 | -3.746974 | -0.5965 |
| ILMN_3168659 | hsa-miR-1183 | 3.42E-04 | 1.0456002 | 1.26E-03 | 3.738989 | -0.6825 |
| ILMN_3168844 | hsa-miR-24-1* | 3.60E-04 | -0.5801428 | 1.31E-03 | -3.723611 | -0.5973 |
| ILMN_3168857 | hsa-miR-1293 | 4.06E-04 | 0.578471 | 1.46E-03 | 3.688193 | -1.216 |
| ILMN_3167686 | hsa-miR-612 | 5.04E-04 | 0.8860108 | 1.77E-03 | 3.623388 | -0.9946 |
| ILMN_3167419 | hsa-miR-376a*:9.1 | 5.23E-04 | -0.7970604 | 1.82E-03 | -3.612245 | -1.1297 |
| ILMN_3168297 | hsa-miR-365 | 5.30E-04 | 0.5034566 | 1.83E-03 | 3.608233 | -1.1759 |
| ILMN_3166988 | hsa-miR-33b | 5.61E-04 | 0.7029895 | 1.93E-03 | 3.591006 | -1.1917 |
| ILMN_3168481 | hsa-miR-377 | 5.83E-04 | -0.6200653 | 1.98E-03 | -3.579622 | -1.0541 |
| ILMN_3167565 | hsa-miR-20b | 6.57E-04 | -0.5339077 | 2.19E-03 | -3.543207 | -1.1902 |
| ILMN_3168464 | hsa-miR-514 | 6.51E-04 | -0.8263912 | 2.19E-03 | -3.5458 | -1.6597 |
| ILMN_3167761 | hsa-miR-212 | 7.24E-04 | 0.6197414 | 2.41E-03 | 3.51335 | -1.4408 |
| ILMN_3168116 | hsa-miR-301a | 7.33E-04 | 0.5091253 | 2.42E-03 | 3.509655 | -1.4102 |
| ILMN_3168167 | hsa-miR-187 | 7.31E-04 | 0.9041354 | 2.42E-03 | 3.510619 | -1.3253 |
| ILMN_3168019 | hsa-miR-206 | 7.40E-04 | -1.4808257 | 2.43E-03 | -3.506947 | -1.8235 |
| ILMN_3168840 | hsa-miR-18b* | 7.70E-04 | 0.8765526 | 2.51E-03 | 3.494732 | -1.3151 |
| ILMN_3168022 | hsa-miR-769-3p | 9.20E-04 | -0.5002475 | 2.93E-03 | -3.439755 | -1.5941 |
| ILMN_3168837 | hsa-miR-190b | 1.13E-03 | -0.8669062 | 3.58E-03 | -3.37506 | -1.8784 |
| ILMN_3168728 | hsa-miR-30c-1* | 1.42E-03 | -0.7740038 | 4.46E-03 | -3.303082 | -1.8979 |
| ILMN_3167593 | hsa-miR-183* | 1.48E-03 | 0.5016474 | 4.61E-03 | 3.290293 | -2.3414 |
| ILMN_3168662 | hsa-miR-15a* | 1.69E-03 | 0.5309458 | 5.15E-03 | 3.248395 | -2.4217 |
| ILMN_3168012 | hsa-miR-142-3p | 1.75E-03 | 0.8318879 | 5.30E-03 | 3.235527 | -2.5239 |
| ILMN_3167447 | hsa-miR-9 | 1.96E-03 | -0.9813956 | 5.83E-03 | -3.199953 | -2.2146 |
| ILMN_3168611 | hsa-miR-149* | 2.09E-03 | 0.532796 | 6.20E-03 | 3.177998 | -2.5888 |
| ILMN_3167360 | hsa-miR-92b | 2.25E-03 | 0.5475227 | 6.64E-03 | 3.154369 | -2.3359 |
| ILMN_3167129 | hsa-miR-202*:9.1 | 2.61E-03 | 0.9311457 | 7.59E-03 | 3.105677 | -2.4764 |
| ILMN_3168756 | hsa-miR-144* | 3.31E-03 | -0.6793917 | 9.35E-03 | -3.026064 | -2.7995 |
| ILMN_3167194 | hsa-miR-9* | 6.03E-03 | -0.6961533 | 1.60E-02 | -2.819943 | -3.2789 |
| ILMN_3168880 | hsa-miR-1303 | 6.79E-03 | 0.5561437 | 1.78E-02 | 2.777438 | -3.7856 |
| ILMN_3168446 | hsa-miR-494 | 7.02E-03 | -0.6461492 | 1.83E-02 | -2.765666 | -3.3562 |
| ILMN_3168691 | hsa-miR-26b* | 9.78E-03 | -0.5401024 | 2.49E-02 | -2.645579 | -3.6554 |
| ILMN_3168842 | hsa-miR-938 | 9.89E-03 | 0.6539056 | 2.51E-02 | 2.641605 | -3.7271 |
| ILMN_3168039 | hsa-miR-124a:9.1 | 1.21E-02 | 0.5511065 | 3.01E-02 | 2.566302 | -4.4348 |
| ILMN_3167969 | hsa-miR-409-3p | 1.64E-02 | -0.5433784 | 3.99E-02 | -2.449415 | -4.1191 |
| ILMN_3167451 | hsa-miR-449a | 1.78E-02 | 0.5222739 | 4.25E-02 | 2.419286 | -4.4252 |

**Table S1b: Differentially expressed miRNA in Periodontitis (GSE54710).**

| **ID** | **miRNA_ID** | **P.Value** | **logFC** | **adj.P.Val** | **t** | **B** |
| --- | --- | --- | --- | --- | --- | --- |
| A_25_P00010919 | hsa-miR-650 | 5.17E-26 | -7.51E-01 | 7.07E-23 | -1.22E+01 | 48.2887 |
| A_25_P00016107 | hsa-miR-3917 | 4.22E-23 | -1.08 | 2.89E-20 | -1.13E+01 | 41.8286 |
| A_25_P00012192 | hsa-miR-145 | 1.66E-12 | -9.48E-01 | 5.67E-10 | -7.53 | 17.9453 |
| A_25_P00012177 | hsa-miR-140-3p | 3.37E-11 | -9.11E-01 | 7.68E-09 | -7.02 | 15.0718 |
| A_25_P00010996 | hsa-miR-210 | 1.06E-10 | 9.55E-01 | 2.08E-08 | 6.82 | 13.9527 |
| A_25_P00014064 | hsa-miR-486-5p | 2.23E-09 | -1.31 | 3.05E-07 | -6.26 | 11.0804 |
| A_25_P00014863 | hsa-miR-202 | 2.87E-09 | -5.42E-01 | 3.57E-07 | -6.22 | 10.4766 |
| A_25_P00015142 | hsa-miR-1246 | 4.33E-09 | 1.4 | 4.56E-07 | 6.14 | 10.4462 |
| A_25_P00012459 | hsa-miR-483-5p | 1.51E-07 | -1.04 | 1.07E-05 | -5.44 | 7.0643 |
| A_25_P00010799 | hsa-miR-663 | 1.57E-07 | -7.49E-01 | 1.07E-05 | -5.44 | 7.0258 |
| A_25_P00012860 | hsa-miR-671-5p | 1.83E-07 | -1.19 | 1.10E-05 | -5.4 | 6.9026 |
| A_25_P00015381 | hsa-miR-205* | 1.97E-07 | 8.61E-01 | 1.12E-05 | 5.39 | 6.7955 |
| A_25_P00015589 | hsa-miR-1260b | 3.01E-07 | 9.79E-01 | 1.64E-05 | 5.3 | 6.4536 |
| A_25_P00015173 | hsa-miR-1260 | 3.68E-07 | 1.03 | 1.93E-05 | 5.26 | 6.2648 |
| A_25_P00014896 | hsa-miR-575 | 3.94E-07 | -9.49E-01 | 2.00E-05 | -5.25 | 6.197 |
| A_25_P00012027 | hsa-miR-33a | 4.14E-07 | 6.96E-01 | 2.02E-05 | 5.24 | 5.9176 |
| A_25_P00016234 | hsa-miR-3679-5p | 7.00E-07 | -8.84E-01 | 3.19E-05 | -5.12 | 5.6468 |
| A_25_P00015265 | hsa-miR-720 | 9.20E-07 | 6.83E-01 | 3.93E-05 | 5.07 | 5.4141 |
| A_25_P00012130 | hsa-miR-223 | 1.04E-06 | -1.24 | 4.31E-05 | -5.04 | 5.29 |
| A_25_P00016236 | hsa-miR-3652 | 1.16E-06 | -8.72E-01 | 4.66E-05 | -5.02 | 5.1101 |
| A_25_P00012446 | hsa-miR-451 | 2.26E-06 | -1.3 | 8.83E-05 | -4.87 | 4.5787 |
| A_25_P00015875 | hsa-miR-3156-5p | 2.73E-06 | 8.23E-01 | 1.04E-04 | 4.83 | 4.3271 |
| A_25_P00015801 | hsa-miR-4306 | 4.47E-06 | -7.48E-01 | 1.65E-04 | -4.72 | 3.8783 |
| A_25_P00010547 | hsa-miR-141 | 8.18E-06 | 8.76E-01 | 2.66E-04 | 4.58 | 3.3804 |
| A_25_P00012270 | hsa-miR-155 | 1.17E-05 | -7.34E-01 | 3.57E-04 | -4.5 | 2.9289 |
| A_25_P00010628 | hsa-miR-203 | 1.45E-05 | 1.09 | 4.21E-04 | 4.45 | 2.8485 |
| A_25_P00015749 | hsa-miR-4299 | 1.65E-05 | -8.11E-01 | 4.70E-04 | -4.42 | 2.7028 |
| A_25_P00012274 | hsa-miR-29c | 1.97E-05 | -6.02E-01 | 5.51E-04 | -4.37 | 2.5629 |
| A_25_P00010132 | hsa-miR-148a | 2.91E-05 | -9.62E-01 | 7.81E-04 | -4.28 | 2.1805 |
| A_25_P00013941 | hsa-miR-125a-3p | 5.25E-05 | -7.03E-01 | 1.35E-03 | -4.13 | 1.537 |
| A_25_P00012013 | hsa-miR-29a | 6.14E-05 | -5.74E-01 | 1.47E-03 | -4.09 | 1.5194 |
| A_25_P00010400 | hsa-miR-204 | 8.84E-05 | 6.94E-01 | 1.86E-03 | 4 | 0.9198 |
| A_25_P00010472 | hsa-miR-99a | 1.49E-04 | 7.35E-01 | 2.72E-03 | 3.87 | 0.6877 |
| A_25_P00015204 | hsa-miR-1274a_v16.0 | 1.66E-04 | 6.67E-01 | 2.99E-03 | 3.84 | 0.5899 |
| A_25_P00013086 | hsa-miR-939 | 1.77E-04 | -5.84E-01 | 3.06E-03 | -3.82 | 0.5034 |
| A_25_P00015644 | hsa-miR-4271 | 1.77E-04 | -6.22E-01 | 3.06E-03 | -3.82 | 0.4136 |
| A_25_P00015818 | hsa-miR-3141 | 2.42E-04 | -6.71E-01 | 4.04E-03 | -3.74 | 0.1237 |
| A_25_P00015304 | hsa-miR-1914* | 2.59E-04 | 5.49E-01 | 4.27E-03 | 3.72 | 0.1003 |
| A_25_P00014822 | hsa-miR-31 | 2.74E-04 | 6.79E-01 | 4.41E-03 | 3.7 | 0.0943 |
| A_25_P00010130 | hsa-miR-497 | 2.81E-04 | -6.57E-01 | 4.46E-03 | -3.7 | 0.0846 |
| A_25_P00012145 | hsa-let-7i | 3.55E-04 | -6.05E-01 | 5.45E-03 | -3.63 | -0.0886 |
| A_25_P00015076 | hsa-miR-1202 | 4.11E-04 | -6.79E-01 | 6.11E-03 | -3.59 | -0.2154 |
| A_25_P00013215 | hsa-miR-31* | 4.18E-04 | 7.05E-01 | 6.15E-03 | 3.59 | -0.3793 |
| A_25_P00011341 | hsa-miR-765 | 6.33E-04 | -5.39E-01 | 8.10E-03 | -3.47 | -0.9321 |
| A_25_P00015774 | hsa-miR-4286 | 6.63E-04 | 5.95E-01 | 8.34E-03 | 3.46 | -0.6573 |
| A_25_P00015303 | hsa-miR-1915 | 8.13E-04 | -5.47E-01 | 9.86E-03 | -3.4 | -0.8467 |
| A_25_P00010249 | hsa-miR-630 | 1.08E-03 | -7.01E-01 | 1.25E-02 | -3.32 | -1.2248 |
| A_25_P00010405 | hsa-miR-211 | 1.22E-03 | 5.81E-01 | 1.38E-02 | 3.28 | -1.7311 |
| A_25_P00012181 | hsa-miR-142-5p | 2.07E-03 | -6.16E-01 | 2.07E-02 | -3.12 | -1.7348 |
| A_25_P00011016 | hsa-miR-142-3p | 2.31E-03 | -6.93E-01 | 2.27E-02 | -3.09 | -1.7839 |
| A_25_P00012239 | hsa-miR-185 | 2.92E-03 | -5.42E-01 | 2.75E-02 | -3.01 | -2.1541 |
| A_25_P00012215 | hsa-miR-126 | 4.01E-03 | -5.97E-01 | 3.47E-02 | -2.91 | -2.2779 |
| A_25_P00010954 | hsa-miR-363 | 4.72E-03 | -5.43E-01 | 3.96E-02 | -2.86 | -2.7872 |
| A_25_P00012133 | hsa-miR-224 | 5.41E-03 | 5.49E-01 | 4.33E-02 | 2.81 | -2.7086 |

**Table S2a. Co-DEmiRNA-Gene Minimum Network-Interactions.**

| **Id** | **Label** | **Degree** | **Betweenness** |
| --- | --- | --- | --- |
| hsa-mir-497-5p | hsa-mir-497-5p | 32 | 591.1171496 |
| hsa-mir-224-5p | hsa-mir-224-5p | 27 | 473.1679678 |
| hsa-mir-210-3p | hsa-mir-210-3p | 24 | 411.9557866 |
| hsa-mir-29c-3p | hsa-mir-29c-3p | 24 | 313.4385143 |
| hsa-mir-486-5p | hsa-mir-486-5p | 22 | 289.3262595 |
| hsa-mir-140-3p | hsa-mir-140-3p | 21 | 259.6328401 |
| hsa-mir-31-5p | hsa-mir-31-5p | 18 | 209.0801609 |
| hsa-mir-33a-5p | hsa-mir-33a-5p | 18 | 154.9131158 |
| hsa-mir-363-3p | hsa-mir-363-3p | 15 | 139.840271 |
| hsa-mir-33a-3p | hsa-mir-33a-3p | 14 | 147.7415243 |
| hsa-mir-1246 | hsa-mir-1246 | 13 | 80.29043969 |
| hsa-mir-363-5p | hsa-mir-363-5p | 12 | 127.5874955 |
| hsa-mir-31-3p | hsa-mir-31-3p | 11 | 73.51803277 |
| ZNF460 | ZNF460 | 11 | 122.2789166 |
| hsa-mir-29c-5p | hsa-mir-29c-5p | 10 | 98.75759364 |
| hsa-mir-497-3p | hsa-mir-497-3p | 9 | 45.3963845 |
| hsa-mir-224-3p | hsa-mir-224-3p | 9 | 90.76835299 |
| hsa-mir-31 | hsa-mir-31 | 8 | 54.03944317 |
| FBN1 | FBN1 | 8 | 83.64164826 |
| CDK6 | CDK6 | 8 | 57.30544937 |
| BTG2 | BTG2 | 8 | 65.7150186 |
| CBX6 | CBX6 | 8 | 60.70221044 |
| DYRK1A | DYRK1A | 8 | 55.28369085 |
| WEE1 | WEE1 | 7 | 42.80113871 |
| BRWD1 | BRWD1 | 7 | 50.54939909 |
| LAMC1 | LAMC1 | 7 | 50.59919956 |
| NUCKS1 | NUCKS1 | 7 | 64.89983592 |
| NOTCH2 | NOTCH2 | 7 | 46.03407976 |
| hsa-mir-210-5p | hsa-mir-210-5p | 6 | 20.66462091 |
| IGF1R | IGF1R | 6 | 50.38566391 |
| PIM1 | PIM1 | 6 | 27.04066625 |
| G3BP2 | G3BP2 | 6 | 27.24966056 |
| FOXP1 | FOXP1 | 6 | 28.47526164 |
| USP14 | USP14 | 6 | 32.91394768 |
| RORA | RORA | 6 | 26.03667316 |
| LMNB2 | LMNB2 | 6 | 31.30573235 |
| RDX | RDX | 6 | 37.09560326 |
| GIGYF1 | GIGYF1 | 6 | 38.51819776 |
| hsa-mir-29c | hsa-mir-29c | 5 | 9.591830961 |
| CDK4 | CDK4 | 5 | 25.10217438 |
| COL4A1 | COL4A1 | 5 | 27.94294411 |
| TMBIM6 | TMBIM6 | 5 | 29.19978489 |
| ZNF207 | ZNF207 | 5 | 21.19736244 |
| ITGA5 | ITGA5 | 5 | 31.37179403 |
| NFIC | NFIC | 5 | 30.43376459 |
| SATB2 | SATB2 | 5 | 26.43382637 |
| LATS2 | LATS2 | 5 | 36.20362215 |
| ABCA1 | ABCA1 | 5 | 95.39827587 |
| LONRF2 | LONRF2 | 5 | 21.79834639 |
| DNAJC10 | DNAJC10 | 5 | 29.29268342 |
| ARF6 | ARF6 | 4 | 9.266048777 |
| ARHGAP5 | ARHGAP5 | 4 | 15.33484319 |
| SERPINE1 | SERPINE1 | 4 | 12.09641587 |
| HAT1 | HAT1 | 4 | 12.65874248 |
| MBD4 | MBD4 | 4 | 13.34591195 |
| BTF3L4 | BTF3L4 | 4 | 21.8519251 |
| ACVR2B | ACVR2B | 4 | 12.08626764 |
| AP2M1 | AP2M1 | 4 | 54.24376192 |
| PABPN1 | PABPN1 | 4 | 13.06570669 |
| RHOA | RHOA | 4 | 14.42837072 |
| RHOBTB1 | RHOBTB1 | 4 | 24.59799914 |
| ZNF217 | ZNF217 | 4 | 13.67342902 |
| EFNA3 | EFNA3 | 4 | 43.66061666 |
| MNT | MNT | 4 | 53.87773186 |
| COL1A1 | COL1A1 | 4 | 17.05347676 |
| COL1A2 | COL1A2 | 4 | 15.23815754 |
| FGF2 | FGF2 | 4 | 18.3030405 |
| FOXO1 | FOXO1 | 3 | 8.545049642 |
| ID4 | ID4 | 3 | 7.676617676 |
| ABCF2 | ABCF2 | 3 | 5.710980837 |
| CLIP1 | CLIP1 | 3 | 6.246716601 |
| CHD7 | CHD7 | 3 | 9.875704574 |
| PPP2R2A | PPP2R2A | 3 | 9.560991137 |
| MEF2D | MEF2D | 3 | 10.64109659 |
| KLK10 | KLK10 | 3 | 35.00917272 |
| PPP2R5C | PPP2R5C | 3 | 12.0083707 |
| SETD1B | SETD1B | 3 | 12.04368006 |
| hsa-mir-210 | hsa-mir-210 | 2 | 1.217458059 |
| hsa-mir-224 | hsa-mir-224 | 2 | 0.954758041 |
| CD40 | CD40 | 2 | 2.150492461 |
| H3F3B | H3F3B | 2 | 1.653760604 |
| GRIN2D | GRIN2D | 2 | 4.305181106 |
| ELAVL1 | ELAVL1 | 2 | 3.612360936 |
| TXN | TXN | 2 | 3.970806112 |
| hsa-mir-33a | hsa-mir-33a | 1 | 0 |

**Table S2b: KEGG Pathway Enrichment Analysis of Co-DEmiRNA-Gene Network.**

| KEGG | Total | Expected | Hits | P.val |
| --- | --- | --- | --- | --- |
| Focal adhesion | 200 | 1.41 | 8 | 5.34e-05 |
| ECM-receptor interaction | 84 | 0.592 | 5 | 0.000264 |
| Pathways in cancer | 310 | 2.19 | 8 | 0.00109 |
| Melanoma | 68 | 0.479 | 4 | 0.00121 |
| Small cell lung cancer | 80 | 0.564 | 4 | 0.00222 |
| Glioma | 65 | 0.458 | 3 | 0.0104 |
| p53 signaling pathway | 68 | 0.479 | 3 | 0.0117 |
| mRNA surveillance pathway | 82 | 0.578 | 3 | 0.0194 |
| TGF-beta signaling pathway | 84 | 0.592 | 3 | 0.0207 |
| Asthma | 7 | 0.0494 | 1 | 0.0484 |
| Tight junction | 118 | 0.832 | 3 | 0.0493 |
| Non-small cell lung cancer | 52 | 0.367 | 2 | 0.0514 |
| Cell cycle | 124 | 0.874 | 3 | 0.0556 |
| Bacterial invasion of epithelial cells | 56 | 0.395 | 2 | 0.0586 |
| Autoimmune thyroid disease | 10 | 0.0705 | 1 | 0.0684 |
| Malaria | 11 | 0.0776 | 1 | 0.075 |
| Pancreatic cancer | 69 | 0.487 | 2 | 0.0844 |
| Adherens junction | 70 | 0.494 | 2 | 0.0865 |
| Chronic myeloid leukemia | 73 | 0.515 | 2 | 0.093 |
| Alcoholism | 166 | 1.17 | 3 | 0.11 |
| Synaptic vesicle cycle | 18 | 0.127 | 1 | 0.12 |
| Prostate cancer | 87 | 0.613 | 2 | 0.125 |
| Regulation of actin cytoskeleton | 182 | 1.28 | 3 | 0.135 |
| Prion diseases | 21 | 0.148 | 1 | 0.138 |
| Toxoplasmosis | 93 | 0.656 | 2 | 0.139 |
| Circadian rhythm - mammal | 22 | 0.155 | 1 | 0.144 |
| Systemic lupus erythematosus | 22 | 0.155 | 1 | 0.144 |
| Viral myocarditis | 26 | 0.183 | 1 | 0.168 |
| Oocyte meiosis | 108 | 0.762 | 2 | 0.176 |
| Leukocyte transendothelial migration | 108 | 0.762 | 2 | 0.176 |
| Huntington's disease | 28 | 0.197 | 1 | 0.18 |
| Intestinal immune Network for IgA production | 29 | 0.204 | 1 | 0.186 |
| Bladder cancer | 29 | 0.204 | 1 | 0.186 |
| Allograft rejection | 29 | 0.204 | 1 | 0.186 |
| Axon guidance | 118 | 0.832 | 2 | 0.202 |
| Dopaminergic synapse | 124 | 0.874 | 2 | 0.217 |
| Pathogenic Escherichia coli infection | 35 | 0.247 | 1 | 0.22 |
| Cocaine addiction | 43 | 0.303 | 1 | 0.263 |
| Wnt signaling pathway | 144 | 1.02 | 2 | 0.27 |
| Lysine degradation | 47 | 0.331 | 1 | 0.284 |
| Notch signaling pathway | 47 | 0.331 | 1 | 0.284 |
| Colorectal cancer | 49 | 0.346 | 1 | 0.294 |
| Pertussis | 52 | 0.367 | 1 | 0.309 |
| Acute myeloid leukemia | 57 | 0.402 | 1 | 0.334 |
| RNA degradation | 60 | 0.423 | 1 | 0.348 |
| Amphetamine addiction | 64 | 0.451 | 1 | 0.366 |
| Complement and coagulation cascades | 67 | 0.472 | 1 | 0.38 |
| Long-term potentiation | 70 | 0.494 | 1 | 0.393 |
| Long-term depression | 70 | 0.494 | 1 | 0.393 |
| HTLV-I infection | 199 | 1.4 | 2 | 0.413 |
| Dilated cardiomyopathy | 78 | 0.55 | 1 | 0.427 |
| Progesterone-mediated oocyte maturation | 80 | 0.564 | 1 | 0.435 |
| Chagas disease (American trypanosomiasis) | 89 | 0.628 | 1 | 0.47 |
| Toll-like receptor signaling pathway | 97 | 0.684 | 1 | 0.5 |
| Fc gamma R-mediated phagocytosis | 97 | 0.684 | 1 | 0.5 |
| T cell receptor signaling pathway | 98 | 0.691 | 1 | 0.504 |
| Jak-STAT signaling pathway | 99 | 0.698 | 1 | 0.507 |
| Hepatitis C | 100 | 0.705 | 1 | 0.511 |
| Endocytosis | 101 | 0.712 | 1 | 0.514 |
| Glutamatergic synapse | 101 | 0.712 | 1 | 0.514 |
| Cytokine-cytokine receptor interaction | 253 | 1.78 | 2 | 0.54 |
| Vascular smooth muscle contraction | 109 | 0.769 | 1 | 0.542 |
| Cell adhesion molecules (CAMs) | 121 | 0.853 | 1 | 0.58 |
| Neurotrophin signaling pathway | 123 | 0.867 | 1 | 0.586 |
| Protein processing in endoplasmic reticulum | 129 | 0.91 | 1 | 0.604 |
| Insulin signaling pathway | 137 | 0.966 | 1 | 0.626 |
| Tuberculosis | 174 | 1.23 | 1 | 0.715 |
| Calcium signaling pathway | 177 | 1.25 | 1 | 0.721 |
| Chemokine signaling pathway | 189 | 1.33 | 1 | 0.745 |
| MAPK signaling pathway | 265 | 1.87 | 1 | 0.855 |

**Table S2c: Reactome Pathway Enrichment Analysis of Co-DEmiRNA-Gene Network.**

| **Reactome** | **Total** | **Expected** | **Hits** | **Pval** |
| --- | --- | --- | --- | --- |
| Signaling by SCF-KIT | 133 | 1.48 | 9 | 1.43E-05 |
| Oncogene Induced Senescence | 30 | 0.333 | 5 | 1.70E-05 |
| Pre-NOTCH Transcription and Translation | 19 | 0.211 | 4 | 4.81E-05 |
| PI3K events in ERBB4 signaling | 94 | 1.04 | 7 | 7.45E-05 |
| PIP3 activates AKT signaling | 94 | 1.04 | 7 | 7.45E-05 |
| PI3K events in ERBB2 signaling | 94 | 1.04 | 7 | 7.45E-05 |
| PI-3K cascade:FGFR1 | 94 | 1.04 | 7 | 7.45E-05 |
| PI-3K cascade:FGFR2 | 94 | 1.04 | 7 | 7.45E-05 |
| PI-3K cascade:FGFR3 | 94 | 1.04 | 7 | 7.45E-05 |
| PI-3K cascade:FGFR4 | 94 | 1.04 | 7 | 7.45E-05 |
| Signaling by EGFR | 168 | 1.87 | 9 | 9.08E-05 |
| PI3K/AKT activation | 97 | 1.08 | 7 | 9.11E-05 |
| GAB1 signalosome | 98 | 1.09 | 7 | 9.72E-05 |
| Pre-NOTCH Expression and Processing | 24 | 0.267 | 4 | 0.000126 |
| Role of LAT2/NTAL/LAB on calcium mobilization | 103 | 1.14 | 7 | 0.000133 |
| Signaling by PDGF | 177 | 1.97 | 9 | 0.000136 |
| Downstream signaling of activated FGFR1 | 139 | 1.54 | 8 | 0.00014 |
| Downstream signaling of activated FGFR2 | 139 | 1.54 | 8 | 0.00014 |
| Downstream signaling of activated FGFR3 | 139 | 1.54 | 8 | 0.00014 |
| Downstream signaling of activated FGFR4 | 139 | 1.54 | 8 | 0.00014 |
| Signaling by ERBB4 | 143 | 1.59 | 8 | 0.00017 |
| Downstream signal transduction | 151 | 1.68 | 8 | 0.000247 |
| Signaling by FGFR | 151 | 1.68 | 8 | 0.000247 |
| Signaling by FGFR1 | 151 | 1.68 | 8 | 0.000247 |
| Signaling by FGFR2 | 151 | 1.68 | 8 | 0.000247 |
| Signaling by FGFR3 | 151 | 1.68 | 8 | 0.000247 |
| Signaling by FGFR4 | 151 | 1.68 | 8 | 0.000247 |
| Signaling by ERBB2 | 152 | 1.69 | 8 | 0.000259 |
| Developmental Biology | 438 | 4.87 | 14 | 0.000275 |
| DAP12 signaling | 154 | 1.71 | 8 | 0.000283 |
| Downstream signaling events of B Cell Receptor (BCR) | 164 | 1.82 | 8 | 0.000433 |
| Fc epsilon receptor (FCERI) signaling | 169 | 1.88 | 8 | 0.00053 |
| DAP12 interactions | 171 | 1.9 | 8 | 0.000573 |
| NGF signalling via TRKA from the plasma membrane | 189 | 2.1 | 8 | 0.00111 |
| Signaling by the B Cell Receptor (BCR) | 190 | 2.11 | 8 | 0.00114 |
| Hemostasis | 450 | 5 | 13 | 0.00121 |
| Transcriptional activity of SMAD2/SMAD3:SMAD4 heterotrimer | 43 | 0.478 | 4 | 0.00125 |
| Downregulation of SMAD2/3:SMAD4 transcriptional activity | 22 | 0.244 | 3 | 0.00174 |
| Constitutive Signaling by AKT1 E17K in Cancer | 22 | 0.244 | 3 | 0.00174 |
| Gene Expression | 851 | 9.46 | 19 | 0.00191 |
| RHO GTPases activate PAKs | 23 | 0.256 | 3 | 0.00199 |
| CDO in myogenesis | 24 | 0.267 | 3 | 0.00226 |
| Myogenesis | 24 | 0.267 | 3 | 0.00226 |
| EPH-Ephrin signaling | 84 | 0.933 | 5 | 0.00231 |
| AKT phosphorylates targets in the nucleus | 7 | 0.0778 | 2 | 0.00247 |
| Post-transcriptional silencing by small RNAs | 7 | 0.0778 | 2 | 0.00247 |
| Adaptive Immune System | 430 | 4.78 | 12 | 0.00255 |
| Signaling by NOTCH | 86 | 0.956 | 5 | 0.00256 |
| Signalling by NGF | 273 | 3.03 | 9 | 0.00307 |
| RHO GTPases Activate WASPs and WAVEs | 30 | 0.333 | 3 | 0.00431 |
| Axon guidance | 292 | 3.24 | 9 | 0.00479 |
| Generic Transcription Pathway | 189 | 2.1 | 7 | 0.00481 |
| Cellular Senescence | 143 | 1.59 | 6 | 0.00494 |
| Methylation | 10 | 0.111 | 2 | 0.00518 |
| Reduction of cytosolic Ca++ levels | 10 | 0.111 | 2 | 0.00518 |
| Signaling by TGF-beta Receptor Complex | 72 | 0.8 | 4 | 0.00819 |
| Ion transport by P-type ATPases | 39 | 0.433 | 3 | 0.00904 |
| Assembly of collagen fibrils and other multimeric structures | 40 | 0.445 | 3 | 0.0097 |
| Binding and Uptake of Ligands by Scavenger Receptors | 40 | 0.445 | 3 | 0.0097 |
| EPHB-mediated forward signaling | 40 | 0.445 | 3 | 0.0097 |
| SOS-mediated signalling | 14 | 0.156 | 2 | 0.0102 |
| PRC2 methylates histones and DNA | 42 | 0.467 | 3 | 0.0111 |
| Factors involved in megakaryocyte development and platelet production | 122 | 1.36 | 5 | 0.0112 |
| RHO GTPases activate CIT | 15 | 0.167 | 2 | 0.0117 |
| RHO GTPases activate IQGAPs | 15 | 0.167 | 2 | 0.0117 |
| PI3K/AKT Signaling in Cancer | 81 | 0.9 | 4 | 0.0123 |
| RHO GTPases Activate ROCKs | 16 | 0.178 | 2 | 0.0132 |
| EPH-ephrin mediated repulsion of cells | 46 | 0.511 | 3 | 0.0142 |
| Signaling by Rho GTPases | 349 | 3.88 | 9 | 0.0147 |
| RHO GTPase Effectors | 234 | 2.6 | 7 | 0.0148 |
| Diseases of signal transduction | 235 | 2.61 | 7 | 0.0151 |
| Vesicle-mediated transport | 184 | 2.04 | 6 | 0.0161 |
| Oxidative Stress Induced Senescence | 88 | 0.978 | 4 | 0.0162 |
| SHC-related events triggered by IGF1R | 18 | 0.2 | 2 | 0.0166 |
| Signaling by NODAL | 19 | 0.211 | 2 | 0.0184 |
| Platelet calcium homeostasis | 19 | 0.211 | 2 | 0.0184 |
| Cell-extracellular matrix interactions | 19 | 0.211 | 2 | 0.0184 |
| Regulation of actin dynamics for phagocytic cup formation | 52 | 0.578 | 3 | 0.0197 |
| Platelet homeostasis | 54 | 0.6 | 3 | 0.0218 |
| Signaling by Leptin | 21 | 0.233 | 2 | 0.0223 |
| Cellular responses to stress | 256 | 2.84 | 7 | 0.023 |
| Sema4D induced cell migration and growth-cone collapse | 22 | 0.244 | 2 | 0.0244 |
| RHO GTPases Activate Formins | 102 | 1.13 | 4 | 0.0264 |
| MicroRNA (miRNA) biogenesis | 23 | 0.256 | 2 | 0.0265 |
| Signaling by Robo receptor | 23 | 0.256 | 2 | 0.0265 |
| Sema4D in semaphorin signaling | 25 | 0.278 | 2 | 0.031 |
| Lysosome Vesicle Biogenesis | 25 | 0.278 | 2 | 0.031 |
| Disease | 669 | 7.43 | 13 | 0.0315 |
| Synthesis of IP3 and IP4 in the cytosol | 27 | 0.3 | 2 | 0.0358 |
| Golgi Associated Vesicle Biogenesis | 27 | 0.3 | 2 | 0.0358 |
| SMAD2/SMAD3:SMAD4 heterotrimer regulates transcription | 28 | 0.311 | 2 | 0.0382 |
| Collagen formation | 70 | 0.778 | 3 | 0.0425 |
| Transcriptional regulation of pluripotent stem cells | 30 | 0.333 | 2 | 0.0434 |
| Cyclin D associated events in G1 | 30 | 0.333 | 2 | 0.0434 |
| G1 Phase | 30 | 0.333 | 2 | 0.0434 |
| Fcgamma receptor (FCGR) dependent phagocytosis | 72 | 0.8 | 3 | 0.0455 |
| Elongation arrest and recovery | 31 | 0.344 | 2 | 0.046 |
| HIV elongation arrest and recovery | 31 | 0.344 | 2 | 0.046 |
| Pausing and recovery of HIV elongation | 31 | 0.344 | 2 | 0.046 |
| Transcriptional regulation by small RNAs | 73 | 0.811 | 3 | 0.0471 |

**Table S2d. Gene Ontology-Biological Process Enrichment Analysis of Co-DEmiRNA-Gene Network.**

| GO-BP | Total | Expected | Hits | Pval |
| --- | --- | --- | --- | --- |
| negative regulation of cellular process | 4110 | 17.2 | 33 | 1.76e-05 |
| Ras protein signal transduction | 274 | 1.15 | 8 | 1.78e-05 |
| neurogenesis | 1390 | 5.84 | 17 | 3.82e-05 |
| generation of neurons | 1300 | 5.48 | 16 | 6.59e-05 |
| response to external stimulus | 1510 | 6.34 | 17 | 0.000108 |
| skeletal system development | 459 | 1.93 | 9 | 0.000116 |
| neuron projection development | 816 | 3.43 | 12 | 0.000123 |
| nervous system development | 2190 | 9.21 | 21 | 0.000136 |
| regulation of cell proliferation | 1430 | 6 | 16 | 0.000193 |
| negative regulation of biological process | 4590 | 19.3 | 33 | 0.000207 |
| axon guidance | 394 | 1.65 | 8 | 0.000227 |
| G1/S transition of mitotic cell cycle | 209 | 0.878 | 6 | 0.000236 |
| regulation of biological quality | 3400 | 14.3 | 27 | 0.000246 |
| neuron differentiation | 1190 | 4.98 | 14 | 0.000314 |
| vasculature development | 652 | 2.74 | 10 | 0.000349 |
| response to endogenous stimulus | 1360 | 5.73 | 15 | 0.000394 |
| neuron development | 945 | 3.97 | 12 | 0.000476 |
| regulation of programmed cell death | 1550 | 6.52 | 16 | 0.000502 |
| regulation of small GTPase mediated signal transduction | 444 | 1.86 | 8 | 0.000506 |
| cell proliferation | 1900 | 7.99 | 18 | 0.000575 |
| axonogenesis | 578 | 2.43 | 9 | 0.000638 |
| transcription from RNA polymerase II promoter | 1930 | 8.1 | 18 | 0.000678 |
| regulation of transcription from RNA polymerase II promoter | 1610 | 6.75 | 16 | 0.000738 |
| small GTPase mediated signal transduction | 596 | 2.5 | 9 | 0.000794 |
| positive regulation of cell migration | 263 | 1.1 | 6 | 8e-04 |
| positive regulation of metabolic process | 2690 | 11.3 | 22 | 0.000886 |
| positive regulation of cellular metabolic process | 2530 | 10.6 | 21 | 0.000986 |
| regulation of cell cycle | 886 | 3.72 | 11 | 0.00102 |
| cell development | 1840 | 7.74 | 17 | 0.00115 |
| regulation of cellular component size | 192 | 0.806 | 5 | 0.00125 |
| regulation of apoptotic process | 1540 | 6.45 | 15 | 0.00136 |
| regulation of protein polymerization | 115 | 0.483 | 4 | 0.00137 |
| Rho protein signal transduction | 121 | 0.508 | 4 | 0.00166 |
| positive regulation of translation | 56 | 0.235 | 3 | 0.00167 |
| positive regulation of transcription from RNA polymerase II promoter | 800 | 3.36 | 10 | 0.00169 |
| negative regulation of apoptotic process | 679 | 2.85 | 9 | 0.00197 |
| negative regulation of apoptotic process | 679 | 2.85 | 9 | 0.00197 |
| organ morphogenesis | 966 | 4.06 | 11 | 0.00205 |
| negative regulation of response to stimulus | 967 | 4.06 | 11 | 0.00206 |
| apoptotic process | 2130 | 8.94 | 18 | 0.00215 |
| apoptotic process | 2130 | 8.94 | 18 | 0.00215 |
| negative regulation of programmed cell death | 691 | 2.9 | 9 | 0.00222 |
| blood coagulation | 564 | 2.37 | 8 | 0.00235 |
| wound healing | 700 | 2.94 | 9 | 0.00243 |
| regulation of nucleobase-containing compound metabolic process | 4540 | 19.1 | 30 | 0.00243 |
| coagulation | 568 | 2.39 | 8 | 0.00246 |
| programmed cell death | 2160 | 9.05 | 18 | 0.00248 |
| hemostasis | 570 | 2.39 | 8 | 0.00251 |
| positive regulation of nucleobase-containing compound metabolic process | 1490 | 6.26 | 14 | 0.00293 |
| enzyme linked receptor protein signaling pathway | 1180 | 4.93 | 12 | 0.00312 |
| cell-substrate adhesion | 241 | 1.01 | 5 | 0.00336 |
| tissue development | 1680 | 7.08 | 15 | 0.00342 |
| response to hormone stimulus | 751 | 3.15 | 9 | 0.00388 |
| regulation of signal transduction | 2440 | 10.2 | 19 | 0.00397 |
| heart development | 487 | 2.05 | 7 | 0.00415 |
| regulation of gene expression | 4480 | 18.8 | 29 | 0.0044 |
| anatomical structure formation involved in morphogenesis | 2090 | 8.78 | 17 | 0.00447 |
| pattern specification process | 503 | 2.11 | 7 | 0.00495 |
| cell division | 507 | 2.13 | 7 | 0.00516 |
| positive regulation of cell proliferation | 786 | 3.3 | 9 | 0.00522 |
| protein polymerization | 167 | 0.701 | 4 | 0.00528 |
| response to organic substance | 2500 | 10.5 | 19 | 0.00532 |
| regulation of RNA metabolic process | 3900 | 16.4 | 26 | 0.00544 |
| regulation of cellular metabolic process | 6120 | 25.7 | 36 | 0.00546 |
| macromolecular complex assembly | 1120 | 4.71 | 11 | 0.00643 |
| regulation of transcription DNA-dependent | 3770 | 15.8 | 25 | 0.00719 |
| regulation of transcription DNA-dependent | 3770 | 15.8 | 25 | 0.00719 |
| regulation of transcription DNA-dependent | 3770 | 15.8 | 25 | 0.00719 |
| cell morphogenesis involved in differentiation | 827 | 3.47 | 9 | 0.00723 |
| regulation of body fluid levels | 680 | 2.86 | 8 | 0.00725 |
| negative regulation of transcription DNA-dependent | 987 | 4.14 | 10 | 0.00756 |
| negative regulation of transcription DNA-dependent | 987 | 4.14 | 10 | 0.00756 |
| positive regulation of RNA metabolic process | 1330 | 5.6 | 12 | 0.00854 |
| cell cycle | 1860 | 7.81 | 15 | 0.0086 |
| regulation of Ras protein signal transduction | 302 | 1.27 | 5 | 0.00863 |
| anatomical structure morphogenesis | 2820 | 11.8 | 20 | 0.00891 |
| protein complex assembly | 861 | 3.62 | 9 | 0.00932 |
| interphase of mitotic cell cycle | 435 | 1.83 | 6 | 0.00962 |
| negative regulation of RNA metabolic process | 1020 | 4.3 | 10 | 0.00969 |
| actin filament polymerization | 107 | 0.449 | 3 | 0.0103 |
| regulation of actin polymerization or depolymerization | 107 | 0.449 | 3 | 0.0103 |
| interphase | 443 | 1.86 | 6 | 0.0105 |
| regulation of actin filament length | 108 | 0.454 | 3 | 0.0105 |
| positive regulation of cytoskeleton organization | 110 | 0.462 | 3 | 0.0111 |
| regulation of cellular protein metabolic process | 1560 | 6.54 | 13 | 0.0113 |
| cell migration | 1050 | 4.43 | 10 | 0.0117 |
| regulation of cell migration | 456 | 1.91 | 6 | 0.012 |
| transforming growth factor beta receptor signaling pathway | 221 | 0.928 | 4 | 0.0138 |
| positive regulation of epithelial cell proliferation | 120 | 0.504 | 3 | 0.014 |
| positive regulation of transcription DNA-dependent | 1260 | 5.28 | 11 | 0.0146 |
| positive regulation of transcription DNA-dependent | 1260 | 5.28 | 11 | 0.0146 |
| establishment or maintenance of cell polarity | 123 | 0.517 | 3 | 0.0149 |
| protein import into nucleus | 228 | 0.957 | 4 | 0.0153 |
| peptidyl-tyrosine phosphorylation | 228 | 0.957 | 4 | 0.0153 |
| peptidyl-tyrosine modification | 230 | 0.966 | 4 | 0.0158 |
| transmembrane receptor protein tyrosine kinase signaling pathway | 782 | 3.28 | 8 | 0.016 |
| central nervous system development | 784 | 3.29 | 8 | 0.0162 |
| nuclear import | 232 | 0.974 | 4 | 0.0163 |
| G1 phase of mitotic cell cycle | 47 | 0.197 | 2 | 0.0166 |
| Cdc42 protein signal transduction | 4 | 0.0168 | 1 | 0.0167 |

**Table S2e. Gene Ontology-Molecular Functions Enrichment Analysis of Co-DEmiRNA-Gene Network**

| GO-MF | Total | Expected | Hits | Pval |
| --- | --- | --- | --- | --- |
| growth factor binding | 125 | 0.501 | 6 | 1.03e-05 |
| extracellular matrix structural constituent | 80 | 0.32 | 5 | 1.65e-05 |
| transcription from RNA polymerase II promoter | 1930 | 7.73 | 18 | 0.000364 |
| nucleotide binding | 2470 | 9.9 | 19 | 0.00263 |
| protein phosphatase type 2A regulator activity | 22 | 0.0881 | 2 | 0.00346 |
| negative regulation of transcription DNA-dependent | 987 | 3.95 | 10 | 0.0054 |
| purine ribonucleotide binding | 1890 | 7.58 | 15 | 0.00642 |
| purine nucleotide binding | 1900 | 7.61 | 15 | 0.00661 |
| protein serine/threonine kinase activity | 572 | 2.29 | 7 | 0.00759 |
| protein serine/threonine/tyrosine kinase activity | 38 | 0.152 | 2 | 0.0101 |
| positive regulation of transcription DNA-dependent | 1260 | 5.04 | 11 | 0.0103 |
| chromatin binding | 338 | 1.35 | 5 | 0.0112 |
| protein complex binding | 339 | 1.36 | 5 | 0.0114 |
| protein tyrosine kinase activity | 222 | 0.889 | 4 | 0.012 |
| protein kinase activity | 960 | 3.85 | 9 | 0.0135 |
| transmembrane receptor protein kinase activity | 125 | 0.501 | 3 | 0.0137 |
| non-membrane spanning protein tyrosine kinase activity | 46 | 0.184 | 2 | 0.0146 |
| structural molecule activity | 666 | 2.67 | 7 | 0.0166 |
| oxidoreductase activity acting on a sulfur group of donors | 65 | 0.26 | 2 | 0.0279 |
| phosphotransferase activity,alcohol group as acceptor | 1110 | 4.43 | 9 | 0.0308 |
| ATP binding | 1490 | 5.96 | 11 | 0.0322 |
| adenyl ribonucleotide binding | 1530 | 6.11 | 11 | 0.0378 |
| adenyl nucleotide binding | 1530 | 6.13 | 11 | 0.0384 |
| phosphatase regulator activity | 80 | 0.32 | 2 | 0.0408 |
| transferase activity, transferring phosphorus-containing groups | 1370 | 5.48 | 10 | 0.0434 |
| kinase activity | 1190 | 4.76 | 9 | 0.0455 |
| enzyme binding | 1200 | 4.8 | 9 | 0.0478 |
| transmembrane receptor protein tyrosine kinase activity | 88 | 0.352 | 2 | 0.0485 |
| transcription factor binding | 509 | 2.04 | 5 | 0.0526 |
| GTP binding | 371 | 1.49 | 4 | 0.0611 |
| nucleoside-triphosphatase activity | 888 | 3.56 | 7 | 0.0632 |
| sequence-specific DNA binding | 732 | 2.93 | 6 | 0.0716 |
| RNA polymerase II distal enhancer sequence-specific DNA binding transcription factor activity | 110 | 0.441 | 2 | 0.0718 |
| GTPase activity | 241 | 0.965 | 3 | 0.0721 |
| guanyl nucleotide binding | 402 | 1.61 | 4 | 0.0771 |
| pyrophosphatase activity | 938 | 3.76 | 7 | 0.0796 |
| hydrolase activity,acting on acid anhydrides | 944 | 3.78 | 7 | 0.0818 |
| hydrolase activity,hydrolyzing N-glycosyl compounds | 22 | 0.0881 | 1 | 0.0846 |
| ionotropic glutamate receptor activity | 23 | 0.0921 | 1 | 0.0882 |
| double-stranded DNA binding | 149 | 0.597 | 2 | 0.12 |
| endodeoxyribonuclease activity | 32 | 0.128 | 1 | 0.121 |
| phospholipid transporter activity | 33 | 0.132 | 1 | 0.124 |
| SH2 domain binding | 33 | 0.132 | 1 | 0.124 |
| electron carrier activity | 158 | 0.633 | 2 | 0.132 |
| glutamate receptor activity | 38 | 0.152 | 1 | 0.142 |
| Rho GTPase activator activity | 39 | 0.156 | 1 | 0.145 |
| sterol binding | 40 | 0.16 | 1 | 0.148 |
| single-stranded RNA binding | 42 | 0.168 | 1 | 0.155 |
| chaperone binding | 46 | 0.184 | 1 | 0.169 |
| deoxyribonuclease activity | 50 | 0.2 | 1 | 0.182 |
| histone acetyltransferase activity | 52 | 0.208 | 1 | 0.189 |
| receptor signaling protein serine/threonine kinase activity | 52 | 0.208 | 1 | 0.189 |
| peptidase inhibitor activity | 207 | 0.829 | 2 | 0.201 |
| steroid hormone receptor activity | 56 | 0.224 | 1 | 0.202 |
| transcription corepressor activity | 208 | 0.833 | 2 | 0.203 |
| ligand-activated sequence-specific DNA binding RNA polymerase II transcription factor activity | 57 | 0.228 | 1 | 0.205 |
| protein dimerization activity | 996 | 3.99 | 6 | 0.207 |
| small conjugating protein-specific protease activity | 59 | 0.236 | 1 | 0.211 |
| histone methyltransferase activity | 60 | 0.24 | 1 | 0.214 |
| protein serine/threonine phosphatase activity | 60 | 0.24 | 1 | 0.214 |
| excitatory extracellular ligand-gated ion channel activity | 61 | 0.244 | 1 | 0.218 |
| ion binding | 6140 | 24.6 | 28 | 0.22 |
| hormone binding | 64 | 0.256 | 1 | 0.227 |
| histone deacetylase binding | 65 | 0.26 | 1 | 0.23 |
| ATPase activity | 417 | 1.67 | 3 | 0.233 |
| SMAD binding | 68 | 0.272 | 1 | 0.239 |
| structure-specific DNA binding | 242 | 0.969 | 2 | 0.253 |
| protein phosphatase binding | 74 | 0.296 | 1 | 0.258 |
| cysteine-type endopeptidase activity | 74 | 0.296 | 1 | 0.258 |
| steroid binding | 82 | 0.328 | 1 | 0.281 |
| N-acetyltransferase activity | 82 | 0.328 | 1 | 0.281 |
| lipid transporter activity | 84 | 0.336 | 1 | 0.287 |
| N-methyltransferase activity | 84 | 0.336 | 1 | 0.287 |
| integrin binding | 86 | 0.344 | 1 | 0.293 |
| protein methyltransferase activity | 86 | 0.344 | 1 | 0.293 |
| identical protein binding | 910 | 3.64 | 5 | 0.3 |
| DNA binding | 2760 | 11 | 13 | 0.304 |
| enzyme regulator activity | 1140 | 4.57 | 6 | 0.306 |
| N-acyltransferase activity | 97 | 0.389 | 1 | 0.323 |
| mRNA binding | 99 | 0.397 | 1 | 0.329 |
| acetyltransferase activity | 99 | 0.397 | 1 | 0.329 |
| extracellular ligand-gated ion channel activity | 104 | 0.417 | 1 | 0.342 |
| cytoskeletal protein binding | 738 | 2.96 | 4 | 0.343 |
| serine-type endopeptidase inhibitor activity | 107 | 0.429 | 1 | 0.35 |
| RNA binding | 976 | 3.91 | 5 | 0.353 |
| transcription coactivator activity | 312 | 1.25 | 2 | 0.356 |
| receptor signaling protein activity | 110 | 0.441 | 1 | 0.358 |
| microtubule binding | 115 | 0.461 | 1 | 0.371 |
| phosphatase binding | 116 | 0.465 | 1 | 0.373 |
| endonuclease activity | 117 | 0.469 | 1 | 0.376 |
| Ras GTPase activator activity | 117 | 0.469 | 1 | 0.376 |
| transcription cofactor activity | 552 | 2.21 | 3 | 0.381 |
| S-adenosylmethionine-dependent methyltransferase activity | 123 | 0.493 | 1 | 0.391 |
| thiolester hydrolase activity | 129 | 0.517 | 1 | 0.405 |
| heparin binding | 130 | 0.521 | 1 | 0.408 |
| unfolded protein binding | 130 | 0.521 | 1 | 0.408 |
| nucleotidyltransferase activity | 135 | 0.541 | 1 | 0.42 |
| protein binding,bridging | 135 | 0.541 | 1 | 0.42 |
| hydrolase activity, acting on glycosyl bonds | 137 | 0.549 | 1 | 0.424 |
| small GTPase binding | 138 | 0.553 | 1 | 0.427 |

**Table S2f. Gene Ontology**-**Cellular Component Enrichment Analysis of Co-DEmiRNA-Gene Network.**

| **GO-CC** | **Total** | **Expected** | **Hits** | **P.val** |
| --- | --- | --- | --- | --- |
| ruffle | 135 | 0.478 | 6 | 7.99E-06 |
| nucleoplasm | 1820 | 6.46 | 16 | 0.000442 |
| cell leading edge | 283 | 1 | 6 | 0.000481 |
| organelle lumen | 3380 | 12 | 23 | 0.000751 |
| extracellular matrix part | 204 | 0.722 | 5 | 0.000765 |
| membrane-enclosed lumen | 3440 | 12.2 | 23 | 0.000965 |
| endoplasmic reticulum lumen | 175 | 0.619 | 4 | 0.00341 |
| nuclear lumen | 2690 | 9.52 | 18 | 0.0043 |
| collagen | 93 | 0.329 | 3 | 0.00436 |
| basement membrane | 100 | 0.354 | 3 | 0.00534 |
| protein serine/threonine phosphatase complex | 48 | 0.17 | 2 | 0.0125 |
| proteinaceous extracellular matrix | 398 | 1.41 | 5 | 0.0132 |
| histone deacetylase complex | 53 | 0.188 | 2 | 0.0151 |
| extracellular matrix | 570 | 2.02 | 6 | 0.0151 |
| nuclear part | 3330 | 11.8 | 19 | 0.0182 |
| chromosome | 784 | 2.77 | 7 | 0.0202 |
| transcription factor complex | 303 | 1.07 | 4 | 0.0223 |
| chromatin | 326 | 1.15 | 4 | 0.0282 |
| chromosomal part | 670 | 2.37 | 6 | 0.0306 |
| cell surface | 518 | 1.83 | 5 | 0.036 |
| nuclear matrix | 87 | 0.308 | 2 | 0.038 |
| nucleoplasm part | 910 | 3.22 | 7 | 0.0408 |
| nucleus | 7600 | 26.9 | 34 | 0.0426 |
| macromolecular complex | 4800 | 17 | 23 | 0.0586 |
| protein complex | 4050 | 14.3 | 20 | 0.062 |
| apical junction complex | 123 | 0.435 | 2 | 0.0704 |
| lamellipodium | 127 | 0.449 | 2 | 0.0744 |
| integral to plasma membrane | 1270 | 4.49 | 8 | 0.0776 |
| cytosol | 2660 | 9.43 | 14 | 0.0785 |
| focal adhesion | 132 | 0.467 | 2 | 0.0795 |
| cell-substrate adherens junction | 138 | 0.488 | 2 | 0.0858 |
| membrane-bounded vesicle | 1100 | 3.89 | 7 | 0.092 |
| intrinsic to plasma membrane | 1320 | 4.68 | 8 | 0.0932 |
| cell-substrate junction | 147 | 0.52 | 2 | 0.0955 |
| basal lamina | 29 | 0.103 | 1 | 0.0978 |
| extracellular space | 901 | 3.19 | 6 | 0.098 |
| nuclear speck | 152 | 0.538 | 2 | 0.101 |
| integrin complex | 32 | 0.113 | 1 | 0.107 |
| cell projection | 1410 | 4.98 | 8 | 0.122 |
| cell-cell junction | 346 | 1.22 | 3 | 0.124 |
| vesicle | 1210 | 4.28 | 7 | 0.134 |
| endocytic vesicle | 187 | 0.662 | 2 | 0.142 |
| receptor complex | 189 | 0.669 | 2 | 0.144 |
| membrane raft | 189 | 0.669 | 2 | 0.144 |
| cell cortex | 195 | 0.69 | 2 | 0.152 |
| chromosome, centromeric region | 198 | 0.701 | 2 | 0.155 |
| intermediate filament | 204 | 0.722 | 2 | 0.163 |
| external side of plasma membrane | 204 | 0.722 | 2 | 0.163 |
| nuclear membrane | 207 | 0.733 | 2 | 0.166 |
| endoplasmic reticulum part | 1060 | 3.73 | 6 | 0.168 |
| adherens junction | 212 | 0.75 | 2 | 0.173 |
| cell junction | 847 | 3 | 5 | 0.18 |
| extracellular region part | 1320 | 4.67 | 7 | 0.184 |
| plasma membrane part | 2320 | 8.19 | 11 | 0.189 |
| extracellular region | 2860 | 10.1 | 13 | 0.201 |
| microvillus | 70 | 0.248 | 1 | 0.22 |
| intermediate filament cytoskeleton | 250 | 0.885 | 2 | 0.222 |
| cytoplasmic vesicle part | 462 | 1.64 | 3 | 0.224 |
| axon | 269 | 0.952 | 2 | 0.246 |
| membrane coat | 89 | 0.315 | 1 | 0.271 |
| coated membrane | 89 | 0.315 | 1 | 0.271 |
| nuclear body | 295 | 1.04 | 2 | 0.281 |
| cytoplasmic membrane-bounded vesicle | 1020 | 3.62 | 5 | 0.295 |
| spindle pole | 101 | 0.357 | 1 | 0.302 |
| tight junction | 105 | 0.372 | 1 | 0.312 |
| synapse | 558 | 1.97 | 3 | 0.316 |
| proteasome complex | 117 | 0.414 | 1 | 0.341 |
| cytoplasmic vesicle | 1110 | 3.93 | 5 | 0.356 |
| extrinsic to membrane | 135 | 0.478 | 1 | 0.382 |
| nuclear envelope | 387 | 1.37 | 2 | 0.399 |
| anchored to membrane | 147 | 0.52 | 1 | 0.408 |
| kinetochore | 149 | 0.527 | 1 | 0.412 |
| cytoplasmic vesicle membrane | 403 | 1.43 | 2 | 0.419 |
| lysosomal membrane | 153 | 0.542 | 1 | 0.42 |
| vesicle membrane | 417 | 1.48 | 2 | 0.436 |
| coated vesicle membrane | 162 | 0.573 | 1 | 0.439 |
| neuron projection | 685 | 2.42 | 3 | 0.439 |
| cell projection part | 686 | 2.43 | 3 | 0.44 |
| organelle part | 8790 | 31.1 | 32 | 0.461 |
| intracellular organelle part | 8620 | 30.5 | 31 | 0.5 |
| vacuolar membrane | 206 | 0.729 | 1 | 0.52 |
| early endosome | 217 | 0.768 | 1 | 0.539 |
| endoplasmic reticulum | 1660 | 5.87 | 6 | 0.541 |
| clathrin-coated vesicle | 224 | 0.793 | 1 | 0.55 |
| envelope | 1130 | 3.99 | 4 | 0.572 |
| microtubule organizing center | 543 | 1.92 | 2 | 0.577 |
| spindle | 261 | 0.924 | 1 | 0.607 |
| secretory granule | 276 | 0.977 | 1 | 0.627 |
| vacuolar part | 279 | 0.987 | 1 | 0.631 |
| coated vesicle | 283 | 1 | 1 | 0.637 |
| apical part of cell | 289 | 1.02 | 1 | 0.644 |
| cell body | 290 | 1.03 | 1 | 0.646 |
| nucleolus | 652 | 2.31 | 2 | 0.677 |
| ribonucleoprotein complex | 681 | 2.41 | 2 | 0.701 |
| endosome | 683 | 2.42 | 2 | 0.702 |
| dendrite | 342 | 1.21 | 1 | 0.706 |
| microtubule | 352 | 1.25 | 1 | 0.717 |
| synapse part | 396 | 1.4 | 1 | 0.759 |
| lytic vacuole | 401 | 1.42 | 1 | 0.763 |
| lysosome | 401 | 1.42 | 1 | 0.763 |

**Table S3. The Transcription Factors of 11 Co-DEmiRNA.**

| Category | Term | Count | Percent | Fold | P-value | FDR |
| --- | --- | --- | --- | --- | --- | --- |
| Transcription factor | BMP2 | 1 | 0.5 | 96.6 | 0.01537646 | 0.4807 |
| Transcription factor | FGF21 | 1 | 0.5 | 96.6 | 0.01537646 | 0.4807 |
| Transcription factor | TTF1 | 1 | 0.5 | 96.6 | 0.01537646 | 0.4807 |
| Transcription factor | ID3 | 1 | 0.33333333 | 64.4 | 0.02045446 | 0.4807 |
| Transcription factor | SOX4 | 1 | 0.33333333 | 64.4 | 0.02045446 | 0.4807 |
| Transcription factor | ZZZ3 | 1 | 0.2 | 38.64 | 0.0305398 | 0.6205 |
| Transcription factor | TNF | 1 | 0.1 | 19.32 | 0.05534557 | 0.8088 |
| Transcription factor | EPAS1 | 2 | 0.05405405 | 10.44324324 | 0.01597795 | 0.4807 |
| Transcription factor | HDAC4 | 1 | 0.05263158 | 10.16842105 | 0.09856065 | 0.8879 |
| Transcription factor | BMI1 | 1 | 0.04761905 | 9.2 | 0.10791889 | 0.8879 |
| Transcription factor | C17orf49 | 1 | 0.04166667 | 8.05 | 0.12179238 | 0.8879 |
| Transcription factor | ARNT | 2 | 0.03703704 | 7.15555556 | 0.03168629 | 0.6205 |
| Transcription factor | SUZ12 | 1 | 0.03703704 | 7.15555556 | 0.13547138 | 0.8879 |
| Transcription factor | TGFB1 | 1 | 0.03225806 | 6.23225806 | 0.15341171 | 0.8879 |
| Transcription factor | SREBF2 | 1 | 0.02941176 | 5.68235294 | 0.1666463 | 0.8879 |
| Transcription factor | NR4A1 | 2 | 0.02857143 | 5.52 | 0.05032134 | 0.7884 |
| Transcription factor | RUNX2 | 3 | 0.02727273 | 5.26909091 | 0.01704524 | 0.4807 |
| Transcription factor | KDM3A | 1 | 0.02702703 | 5.22162162 | 0.1796944 | 0.8879 |
| Transcription factor | PDX1 | 1 | 0.02702703 | 5.22162162 | 0.1796944 | 0.8879 |
| Transcription factor | GATAD1 | 1 | 0.02173913 | 4.2 | 0.21774299 | 0.8879 |
| Transcription factor | TP73 | 2 | 0.02040816 | 3.94285714 | 0.09021255 | 0.8879 |
| Transcription factor | FOXK1 | 1 | 0.02 | 3.864 | 0.23413765 | 0.8879 |
| Transcription factor | NCOR2 | 1 | 0.01886792 | 3.64528302 | 0.24622999 | 0.8879 |
| Transcription factor | ETV5 | 1 | 0.01754386 | 3.38947368 | 0.26208576 | 0.8879 |
| Transcription factor | SP4 | 1 | 0.01754386 | 3.38947368 | 0.26208576 | 0.8879 |
| Transcription factor | ING5 | 2 | 0.0173913 | 3.36 | 0.11801125 | 0.8879 |
| Transcription factor | SOX9 | 2 | 0.0173913 | 3.36 | 0.11801125 | 0.8879 |
| Transcription factor | NFE2L2 | 3 | 0.01685393 | 3.25617978 | 0.05850533 | 0.8088 |
| Transcription factor | BCL3 | 2 | 0.01652893 | 3.19338843 | 0.12834033 | 0.8879 |
| Transcription factor | ELK1 | 2 | 0.01612903 | 3.11612903 | 0.13359448 | 0.8879 |
| Transcription factor | MAFF | 3 | 0.01578947 | 3.05052632 | 0.06864271 | 0.8879 |
| Transcription factor | HDAC3 | 1 | 0.015625 | 3.01875 | 0.2891126 | 0.8879 |
| Transcription factor | PIAS1 | 1 | 0.01515152 | 2.92727273 | 0.29666915 | 0.8879 |
| Transcription factor | BRD1 | 2 | 0.01503759 | 2.90526316 | 0.14968485 | 0.8879 |
| Transcription factor | NFKB1 | 5 | 0.01492537 | 2.88358209 | 0.01872138 | 0.4807 |
| Transcription factor | NR1H2 | 1 | 0.01492537 | 2.88358209 | 0.30042025 | 0.8879 |
| Transcription factor | GTF2I | 6 | 0.01385681 | 2.67713626 | 0.01172511 | 0.4807 |
| Transcription factor | ESRRA | 2 | 0.0137931 | 2.66482759 | 0.17181327 | 0.8879 |
| Transcription factor | THAP1 | 1 | 0.01369863 | 2.64657534 | 0.32255121 | 0.8879 |
| Transcription factor | NFYC | 1 | 0.01333333 | 2.576 | 0.32978692 | 0.8879 |
| Transcription factor | HMGN3 | 2 | 0.01315789 | 2.54210526 | 0.18502466 | 0.8879 |
| Transcription factor | MITF | 2 | 0.01315789 | 2.54210526 | 0.18502466 | 0.8879 |
| Transcription factor | POU5F1 | 1 | 0.01282051 | 2.47692308 | 0.34051013 | 0.8879 |
| Transcription factor | SREBF1 | 1 | 0.0125 | 2.415 | 0.34757289 | 0.8879 |
| Transcription factor | ATF4 | 1 | 0.01219512 | 2.35609756 | 0.3545675 | 0.8879 |
| Transcription factor | INTS11 | 1 | 0.01204819 | 2.32771084 | 0.35803944 | 0.8879 |
| Transcription factor | ZBTB17 | 1 | 0.01190476 | 2.3 | 0.36149455 | 0.8879 |
| Transcription factor | POU2F2 | 4 | 0.01179941 | 2.27964602 | 0.08286471 | 0.8879 |
| Transcription factor | CCNT2 | 3 | 0.01171875 | 2.2640625 | 0.13810199 | 0.8879 |
| Transcription factor | PAX5 | 3 | 0.01115242 | 2.15464684 | 0.1542119 | 0.8879 |
| Transcription factor | IRF4 | 2 | 0.01104972 | 2.13480663 | 0.24149134 | 0.8879 |
| Transcription factor | SMAD3 | 2 | 0.01104972 | 2.13480663 | 0.24149134 | 0.8879 |
| Transcription factor | SNAI2 | 6 | 0.01094891 | 2.11532847 | 0.03698386 | 0.6686 |
| Transcription factor | ATF1 | 2 | 0.01075269 | 2.07741935 | 0.25142752 | 0.8879 |
| Transcription factor | CDK8 | 4 | 0.01066667 | 2.0608 | 0.11195758 | 0.8879 |
| Transcription factor | E2F6 | 8 | 0.01041667 | 2.0125 | 0.01202277 | 0.4807 |
| Transcription factor | NR2F1 | 4 | 0.01033592 | 1.99689922 | 0.12269815 | 0.8879 |
| Transcription factor | CDK7 | 2 | 0.01015228 | 1.96142132 | 0.27341026 | 0.8879 |
| Transcription factor | ZNF750 | 1 | 0.00980392 | 1.89411765 | 0.42089209 | 0.8879 |
| Transcription factor | SP2 | 3 | 0.0096463 | 1.86366559 | 0.21062386 | 0.8879 |
| Transcription factor | NKX2-1 | 2 | 0.00956938 | 1.84880383 | 0.29751051 | 0.8879 |
| Transcription factor | LMO2 | 1 | 0.00952381 | 1.84 | 0.43029301 | 0.8879 |
| Transcription factor | MAFK | 3 | 0.00946372 | 1.82839117 | 0.2191513 | 0.8879 |
| Transcription factor | NELFE | 4 | 0.00911162 | 1.76036446 | 0.17489167 | 0.8879 |
| Transcription factor | KLF4 | 1 | 0.00909091 | 1.75636364 | 0.44565638 | 0.8879 |
| Transcription factor | NFIC | 4 | 0.00907029 | 1.75238095 | 0.17707179 | 0.8879 |
| Transcription factor | BACH1 | 1 | 0.00892857 | 1.725 | 0.45169658 | 0.8879 |
| Transcription factor | TP53 | 5 | 0.00891266 | 1.72192513 | 0.13541929 | 0.8879 |
| Transcription factor | RCOR1 | 3 | 0.00887574 | 1.7147929 | 0.249748 | 0.8879 |
| Transcription factor | JUN | 6 | 0.00863309 | 1.66791367 | 0.10789044 | 0.8879 |
| Transcription factor | E2F1 | 8 | 0.00855615 | 1.65304813 | 0.04484746 | 0.7528 |
| Transcription factor | CHD1 | 1 | 0.00854701 | 1.65128205 | 0.46653879 | 0.8879 |
| Transcription factor | ETV1 | 1 | 0.00854701 | 1.65128205 | 0.46653879 | 0.8879 |
| Transcription factor | SMC3 | 4 | 0.00845666 | 1.63382664 | 0.21352328 | 0.8879 |
| Transcription factor | TCF4 | 4 | 0.00835073 | 1.61336117 | 0.220668 | 0.8879 |
| Transcription factor | CNOT3 | 5 | 0.00819672 | 1.58360656 | 0.17977908 | 0.8879 |
| Transcription factor | PBX1 | 4 | 0.00809717 | 1.56437247 | 0.23892148 | 0.8879 |
| Transcription factor | MYCN | 6 | 0.00808625 | 1.56226415 | 0.14191505 | 0.8879 |
| Transcription factor | SUPT5H | 5 | 0.00778816 | 1.5046729 | 0.21224693 | 0.8879 |
| Transcription factor | LARP7 | 7 | 0.00778643 | 1.50433815 | 0.12183349 | 0.8879 |
| Transcription factor | HDAC1 | 2 | 0.0077821 | 1.50350195 | 0.39346412 | 0.8879 |
| Transcription factor | FOS | 5 | 0.00777605 | 1.50233281 | 0.21330338 | 0.8879 |
| Transcription factor | AFF4 | 1 | 0.00769231 | 1.48615385 | 0.50345132 | 0.8879 |
| Transcription factor | SIX2 | 1 | 0.00763359 | 1.47480916 | 0.50619317 | 0.8879 |
| Transcription factor | NFYB | 3 | 0.00759494 | 1.46734177 | 0.33704904 | 0.8879 |
| Transcription factor | NFYA | 3 | 0.0074813 | 1.44538653 | 0.34646477 | 0.8879 |
| Transcription factor | NFE2 | 5 | 0.00747384 | 1.44394619 | 0.24160949 | 0.8879 |
| Transcription factor | ZBTB7A | 4 | 0.00744879 | 1.43910615 | 0.29396955 | 0.8879 |
| Transcription factor | ZMYND8 | 1 | 0.00735294 | 1.42058824 | 0.51969911 | 0.8879 |
| Transcription factor | TFAP2A | 6 | 0.00728155 | 1.40679612 | 0.21506635 | 0.8879 |
| Transcription factor | TP63 | 2 | 0.00727273 | 1.40509091 | 0.42862526 | 0.8879 |
| Transcription factor | OTX2 | 1 | 0.00724638 | 1.4 | 0.52500773 | 0.8879 |
| Transcription factor | BCLAF1 | 1 | 0.00719424 | 1.38992806 | 0.52764218 | 0.8879 |
| Transcription factor | JMJD1C | 2 | 0.00716846 | 1.38494624 | 0.4363428 | 0.8879 |
| Transcription factor | PML | 3 | 0.0071599 | 1.38329356 | 0.37482785 | 0.8879 |
| Transcription factor | KLF5 | 2 | 0.00701754 | 1.35578947 | 0.44784568 | 0.8879 |
| Transcription factor | RUNX1T1 | 2 | 0.00696864 | 1.34634146 | 0.45165955 | 0.8879 |
| Transcription factor | CTCFL | 6 | 0.00696056 | 1.34477958 | 0.25460599 | 0.8879 |
| Transcription factor | RUNX1 | 8 | 0.00694444 | 1.34166667 | 0.16234701 | 0.8879 |
| Transcription factor | DDX5 | 1 | 0.00689655 | 1.33241379 | 0.54317464 | 0.8879 |
| Transcription factor | NR3C1 | 5 | 0.00684932 | 1.32328767 | 0.31365729 | 0.8879 |
| Transcription factor | RELA | 6 | 0.00677201 | 1.30835214 | 0.28124749 | 0.8879 |
| Transcription factor | LMNA | 1 | 0.00675676 | 1.30540541 | 0.55076715 | 0.8879 |
| Transcription factor | TAF1 | 6 | 0.00657895 | 1.27105263 | 0.31143582 | 0.8879 |
| Transcription factor | TRIM24 | 3 | 0.00657895 | 1.27105263 | 0.43320674 | 0.8879 |
| Transcription factor | FOXO1 | 3 | 0.00645161 | 1.24645161 | 0.44732818 | 0.8879 |
| Transcription factor | KDM2B | 6 | 0.00645161 | 1.24645161 | 0.33307072 | 0.8879 |
| Transcription factor | NIPBL | 1 | 0.00636943 | 1.23057325 | 0.57286818 | 0.8879 |
| Transcription factor | GTF2B | 2 | 0.00634921 | 1.22666667 | 0.50386196 | 0.8879 |
| Transcription factor | WDR5 | 5 | 0.00632111 | 1.22123894 | 0.39167983 | 0.8879 |
| Transcription factor | NR2F2 | 3 | 0.00631579 | 1.22021053 | 0.46294707 | 0.8879 |
| Transcription factor | BRPF3 | 2 | 0.00626959 | 1.21128527 | 0.51112249 | 0.8879 |
| Transcription factor | MYOD1 | 2 | 0.00626959 | 1.21128527 | 0.51112249 | 0.8879 |
| Transcription factor | SRF | 4 | 0.00624025 | 1.20561622 | 0.4374462 | 0.8879 |
| Transcription factor | HOXA4 | 1 | 0.00617284 | 1.19259259 | 0.58471905 | 0.8879 |
| Transcription factor | MYB | 4 | 0.00617284 | 1.19259259 | 0.44731384 | 0.8879 |
| Transcription factor | AR | 7 | 0.00612423 | 1.1832021 | 0.36242106 | 0.8879 |
| Transcription factor | RBPJ | 1 | 0.00609756 | 1.17804878 | 0.58937594 | 0.8879 |
| Transcription factor | ZEB1 | 1 | 0.00609756 | 1.17804878 | 0.58937594 | 0.8879 |
| Transcription factor | NRF1 | 4 | 0.0060698 | 1.17268589 | 0.46281115 | 0.8879 |
| Transcription factor | EGR1 | 5 | 0.00606061 | 1.17090909 | 0.43675826 | 0.8879 |
| Transcription factor | ZNF143 | 5 | 0.00604595 | 1.16807739 | 0.43943053 | 0.8879 |
| Transcription factor | TCF3 | 6 | 0.00603622 | 1.16619718 | 0.413968 | 0.8879 |
| Transcription factor | MED1 | 5 | 0.00603136 | 1.16525935 | 0.44210444 | 0.8879 |
| Transcription factor | ASXL1 | 3 | 0.00598802 | 1.15688623 | 0.503075 | 0.8879 |
| Transcription factor | ERG | 7 | 0.00598802 | 1.15688623 | 0.39596328 | 0.8879 |
| Transcription factor | HSF1 | 1 | 0.00598802 | 1.15688623 | 0.59627317 | 0.8879 |
| Transcription factor | CTCF | 7 | 0.00598291 | 1.15589744 | 0.39727561 | 0.8879 |
| Transcription factor | HEY1 | 5 | 0.00597372 | 1.15412186 | 0.45281423 | 0.8879 |
| Transcription factor | SETD1A | 3 | 0.00596421 | 1.15228628 | 0.50612683 | 0.8879 |
| Transcription factor | ETS1 | 4 | 0.00592593 | 1.14488889 | 0.48529668 | 0.8879 |
| Transcription factor | ING2 | 2 | 0.00591716 | 1.14319527 | 0.54487038 | 0.8879 |
| Transcription factor | PHF8 | 5 | 0.00577367 | 1.11547344 | 0.49173626 | 0.8879 |
| Transcription factor | TCF7L2 | 3 | 0.00572519 | 1.10610687 | 0.53780658 | 0.8879 |
| Transcription factor | MXI1 | 4 | 0.0056899 | 1.09928876 | 0.52432375 | 0.8879 |
| Transcription factor | TFAP4 | 4 | 0.00567376 | 1.09617021 | 0.52708928 | 0.8879 |
| Transcription factor | ATF3 | 2 | 0.00566572 | 1.09461756 | 0.57060329 | 0.8879 |
| Transcription factor | HOXA6 | 1 | 0.00564972 | 1.09152542 | 0.61851753 | 0.8879 |
| Transcription factor | RUNX3 | 4 | 0.00564972 | 1.09152542 | 0.53123093 | 0.8879 |
| Transcription factor | BHLHE40 | 3 | 0.0056391 | 1.08947368 | 0.54968221 | 0.8879 |
| Transcription factor | UBTF | 1 | 0.00561798 | 1.08539326 | 0.62068013 | 0.8879 |
| Transcription factor | FOXA2 | 3 | 0.00557621 | 1.07732342 | 0.55851225 | 0.8879 |
| Transcription factor | RAD21 | 5 | 0.00557414 | 1.07692308 | 0.53323964 | 0.8879 |
| Transcription factor | JARID2 | 1 | 0.00555556 | 1.07333333 | 0.62497226 | 0.8879 |
| Transcription factor | STAT3 | 4 | 0.00555556 | 1.07333333 | 0.54771116 | 0.8879 |
| Transcription factor | PPARG | 2 | 0.00552486 | 1.06740331 | 0.58563858 | 0.8879 |
| Transcription factor | CDK9 | 3 | 0.00548446 | 1.05959781 | 0.57162732 | 0.8879 |
| Transcription factor | FGFR1 | 3 | 0.00544465 | 1.05190563 | 0.57740411 | 0.8879 |
| Transcription factor | KDM5B | 6 | 0.00540541 | 1.04432432 | 0.56858098 | 0.8879 |
| Transcription factor | SUMO2 | 4 | 0.00540541 | 1.04432432 | 0.57482394 | 0.8879 |
| Transcription factor | TCF12 | 5 | 0.00539957 | 1.04319654 | 0.57163905 | 0.8879 |
| Transcription factor | FOSL2 | 2 | 0.00539084 | 1.04150943 | 0.60036171 | 0.8879 |
| Transcription factor | ATF2 | 2 | 0.00536193 | 1.03592493 | 0.60359051 | 0.8879 |
| Transcription factor | HIF1A | 6 | 0.00535714 | 1.035 | 0.58189328 | 0.8879 |
| Transcription factor | SMC1A | 4 | 0.00535475 | 1.03453815 | 0.58419384 | 0.8879 |
| Transcription factor | TEAD4 | 3 | 0.00534759 | 1.03315508 | 0.5916994 | 0.8879 |
| Transcription factor | MAX | 7 | 0.00531915 | 1.02765957 | 0.5991479 | 0.8879 |
| Transcription factor | TBP | 4 | 0.00531915 | 1.02765957 | 0.59084493 | 0.8879 |
| Transcription factor | EBF1 | 3 | 0.00530973 | 1.02584071 | 0.59735694 | 0.8879 |
| Transcription factor | BRD3 | 2 | 0.00529101 | 1.02222222 | 0.61159339 | 0.8879 |
| Transcription factor | TRIM28 | 5 | 0.00528541 | 1.02114165 | 0.59771995 | 0.8879 |
| Transcription factor | GABPA | 4 | 0.0052356 | 1.01151832 | 0.60665668 | 0.8879 |
| Transcription factor | KDM4C | 2 | 0.00520833 | 1.00625 | 0.62106556 | 0.8879 |
| Transcription factor | KLF1 | 4 | 0.00516129 | 0.99716129 | 0.62095103 | 0.8879 |
| Transcription factor | HNF4G | 1 | 0.00515464 | 0.99587629 | 0.6538139 | 0.8879 |
| Transcription factor | BATF | 1 | 0.00512821 | 0.99076923 | 0.6557955 | 0.8879 |
| Transcription factor | CEBPD | 1 | 0.00512821 | 0.99076923 | 0.6557955 | 0.8879 |
| Transcription factor | DNMT3A | 1 | 0.00512821 | 0.99076923 | 0.6557955 | 0.8879 |
| Transcription factor | ESR1 | 6 | 0.00512383 | 0.98992314 | 0.64855486 | 0.8879 |
| Transcription factor | CBFB | 2 | 0.00506329 | 0.97822785 | 0.63805538 | 0.8879 |
| Transcription factor | MYC | 6 | 0.00505902 | 0.97740304 | 0.66761091 | 0.8879 |
| Transcription factor | TRIM25 | 3 | 0.00497512 | 0.96119403 | 0.64923777 | 0.8879 |
| Transcription factor | ARNTL | 4 | 0.00496894 | 0.96 | 0.65885613 | 0.8879 |
| Transcription factor | GATA4 | 2 | 0.00492611 | 0.95172414 | 0.65455367 | 0.8879 |
| Transcription factor | TFAP2C | 4 | 0.004884 | 0.94358974 | 0.67595314 | 0.8879 |
| Transcription factor | STAG1 | 3 | 0.00487805 | 0.94243902 | 0.66487238 | 0.8879 |
| Transcription factor | JUND | 4 | 0.00487211 | 0.94129111 | 0.67836276 | 0.8879 |
| Transcription factor | FOXA1 | 6 | 0.00486224 | 0.93938412 | 0.72622093 | 0.8888 |
| Transcription factor | NOTCH1 | 4 | 0.00485437 | 0.93786408 | 0.68196146 | 0.8879 |
| Transcription factor | REST | 3 | 0.00484653 | 0.93634895 | 0.66999967 | 0.8879 |
| Transcription factor | CHD8 | 4 | 0.00480769 | 0.92884615 | 0.69146425 | 0.8879 |
| Transcription factor | EP300 | 6 | 0.00479233 | 0.92587859 | 0.74709232 | 0.8888 |
| Transcription factor | KMT2D | 3 | 0.00477707 | 0.92292994 | 0.68137892 | 0.8879 |
| Transcription factor | KMT2A | 3 | 0.00472441 | 0.91275591 | 0.69007687 | 0.8879 |
| Transcription factor | KDM4A | 1 | 0.00471698 | 0.91132075 | 0.68795759 | 0.8879 |
| Transcription factor | ELF1 | 4 | 0.00463499 | 0.89548088 | 0.72694278 | 0.8888 |
| Transcription factor | FLI1 | 4 | 0.00454545 | 0.87818182 | 0.74544605 | 0.8888 |
| Transcription factor | IRF1 | 3 | 0.0045045 | 0.87027027 | 0.72694696 | 0.8888 |
| Transcription factor | KAT7 | 1 | 0.0045045 | 0.87027027 | 0.70558735 | 0.8888 |
| Transcription factor | MAZ | 4 | 0.00449944 | 0.86929134 | 0.75495751 | 0.8888 |
| Transcription factor | BRD4 | 5 | 0.00446828 | 0.86327078 | 0.7967048 | 0.8925 |
| Transcription factor | CEBPB | 4 | 0.00443459 | 0.85676275 | 0.76834108 | 0.8888 |
| Transcription factor | TAF3 | 2 | 0.00436681 | 0.84366812 | 0.72580793 | 0.8888 |
| Transcription factor | BCL6 | 2 | 0.00434783 | 0.84 | 0.72832636 | 0.8888 |
| Transcription factor | NFATC1 | 1 | 0.00434783 | 0.84 | 0.71904004 | 0.8888 |
| Transcription factor | RFX5 | 1 | 0.00434783 | 0.84 | 0.71904004 | 0.8888 |
| Transcription factor | STAT1 | 4 | 0.004329 | 0.83636364 | 0.79001528 | 0.8925 |
| Transcription factor | CREB1 | 4 | 0.0042508 | 0.82125399 | 0.80590967 | 0.8956 |
| Transcription factor | SOX2 | 1 | 0.00420168 | 0.81176471 | 0.73193695 | 0.8888 |
| Transcription factor | BCOR | 2 | 0.00414079 | 0.8 | 0.75612085 | 0.8888 |
| Transcription factor | GATA2 | 2 | 0.00411523 | 0.79506173 | 0.75958918 | 0.8888 |
| Transcription factor | SP1 | 4 | 0.00409417 | 0.79099284 | 0.83707724 | 0.9024 |
| Transcription factor | BRCA1 | 1 | 0.004 | 0.7728 | 0.75028489 | 0.8888 |
| Transcription factor | KLF11 | 2 | 0.004 | 0.7728 | 0.77530245 | 0.8888 |
| Transcription factor | KDM1A | 1 | 0.00398406 | 0.76972112 | 0.75176162 | 0.8888 |
| Transcription factor | LEO1 | 1 | 0.00393701 | 0.76062992 | 0.75614478 | 0.8888 |
| Transcription factor | SIN3A | 3 | 0.00388098 | 0.74980595 | 0.8327111 | 0.9024 |
| Transcription factor | HDAC2 | 1 | 0.00384615 | 0.74307692 | 0.76470274 | 0.8888 |
| Transcription factor | CHD2 | 2 | 0.00383877 | 0.74165067 | 0.79743837 | 0.8925 |
| Transcription factor | GATA1 | 3 | 0.00383632 | 0.74117647 | 0.84008547 | 0.9024 |
| Transcription factor | MYH11 | 1 | 0.00383142 | 0.74022989 | 0.76610249 | 0.8888 |
| Transcription factor | YY1 | 3 | 0.00380711 | 0.73553299 | 0.84487393 | 0.9024 |
| Transcription factor | USF2 | 1 | 0.00377358 | 0.7290566 | 0.77162684 | 0.8888 |
| Transcription factor | MBD3 | 3 | 0.00369458 | 0.7137931 | 0.863021 | 0.9095 |
| Transcription factor | TAL1 | 3 | 0.00359712 | 0.69496403 | 0.87827457 | 0.9202 |
| Transcription factor | RARA | 2 | 0.00353982 | 0.68389381 | 0.83847355 | 0.9024 |
| Transcription factor | ELF3 | 1 | 0.00353357 | 0.68268551 | 0.79506171 | 0.8925 |
| Transcription factor | HNF4A | 1 | 0.00350877 | 0.67789474 | 0.79752711 | 0.8925 |
| Transcription factor | CBX3 | 2 | 0.00344828 | 0.6662069 | 0.85089318 | 0.9024 |
| Transcription factor | USF1 | 2 | 0.00343643 | 0.66391753 | 0.85249151 | 0.9024 |
| Transcription factor | YAP1 | 1 | 0.00338983 | 0.65491525 | 0.80945837 | 0.8956 |
| Transcription factor | FOSL1 | 1 | 0.003367 | 0.65050505 | 0.81176724 | 0.8956 |
| Transcription factor | CXXC1 | 2 | 0.00322061 | 0.62222222 | 0.88107224 | 0.9202 |
| Transcription factor | PGR | 1 | 0.00315457 | 0.60946372 | 0.83350916 | 0.9024 |
| Transcription factor | CEBPA | 1 | 0.00307692 | 0.59446154 | 0.84155306 | 0.9024 |
| Transcription factor | RBBP5 | 1 | 0.00263158 | 0.50842105 | 0.88803778 | 0.9234 |
| Transcription factor | ZNF263 | 1 | 0.00253165 | 0.48911392 | 0.89837152 | 0.93 |
| Transcription factor | SMAD1 | 1 | 0.00240385 | 0.46442308 | 0.91139787 | 0.9371 |
| Transcription factor | E2F4 | 1 | 0.00238663 | 0.46109785 | 0.91313059 | 0.9371 |
| Transcription factor | HCFC1 | 1 | 0.00233645 | 0.45140187 | 0.91814731 | 0.9379 |
| Transcription factor | CREBBP | 1 | 0.00227273 | 0.43909091 | 0.92443044 | 0.9379 |
| Transcription factor | PBX3 | 1 | 0.00225734 | 0.43611738 | 0.92593178 | 0.9379 |
| Transcription factor | GATA3 | 2 | 0.00215517 | 0.41637931 | 0.98515147 | 0.9894 |
| Transcription factor | EZH2 | 1 | 0.00175131 | 0.33835377 | 0.96976604 | 0.9781 |
| Transcription factor | SPI1 | 1 | 0.0009434 | 0.18226415 | 0.99964101 | 0.9996 |

**Table S4a. Co-DEmiRNA-TF Minimum Network-Interactions.**

| Id | Label | Degree | Betweenness |
| --- | --- | --- | --- |
| hsa-mir-224 | hsa-mir-224 | 4 | 47.28333333 |
| hsa-mir-210 | hsa-mir-210 | 4 | 25.1 |
| HIF1A | HIF1A | 4 | 40.01666667 |
| hsa-mir-29c | hsa-mir-29c | 3 | 14.05 |
| hsa-mir-31 | hsa-mir-31 | 3 | 9.116666667 |
| hsa-mir-363 | hsa-mir-363 | 3 | 31.41666667 |
| TP53 | TP53 | 3 | 20.21666667 |
| hsa-mir-497 | hsa-mir-497 | 2 | 16.03333333 |
| E2F1 | E2F1 | 2 | 27.96666667 |
| ELK1 | ELK1 | 2 | 4.683333333 |
| EPAS1 | EPAS1 | 2 | 2.233333333 |
| ESR1 | ESR1 | 2 | 10.45 |
| JUN | JUN | 2 | 10.98333333 |
| MYCN | MYCN | 2 | 15 |
| YY1 | YY1 | 2 | 3.45 |
| hsa-mir-33a | hsa-mir-33a | 1 | 0 |
| hsa-mir-1246 | hsa-mir-1246 | 1 | 0 |

**Table S4b. KEGG Pathway Enrichment Analysis of Co-DEmiRNA-TF Network.**

| KEGG Pathway | Total | Expected | Hits | P value |
| --- | --- | --- | --- | --- |
| Acute myeloid leukemia | 57 | 0.11 | 5 | 2.72E-08 |
| Pathways in cancer | 310 | 0.596 | 6 | 5.71E-06 |
| Chronic myeloid leukemia | 73 | 0.14 | 3 | 0.000286 |
| Epstein-Barr virus infection | 91 | 0.175 | 3 | 0.000549 |
| Transcriptional misregulation in cancer | 19 | 0.0365 | 2 | 0.000553 |
| Adipocytokine signaling pathway | 63 | 0.121 | 2 | 0.00604 |
| Pancreatic cancer | 69 | 0.133 | 2 | 0.00721 |
| Small cell lung cancer | 80 | 0.154 | 2 | 0.00961 |
| Toxoplasmosis | 93 | 0.179 | 2 | 0.0128 |
| Jak-STAT signaling pathway | 99 | 0.19 | 2 | 0.0145 |
| Measles | 102 | 0.196 | 2 | 0.0153 |
| Cytosolic DNA-sensing pathway | 20 | 0.0385 | 1 | 0.0378 |
| Tuberculosis | 174 | 0.335 | 2 | 0.0417 |
| Chemokine signaling pathway | 189 | 0.363 | 2 | 0.0484 |
| Thyroid cancer | 28 | 0.0538 | 1 | 0.0526 |
| Epithelial cell signaling in Helicobacter pylori infection | 37 | 0.0712 | 1 | 0.069 |
| Legionellosis | 40 | 0.0769 | 1 | 0.0744 |
| Cocaine addiction | 43 | 0.0827 | 1 | 0.0798 |
| Endometrial cancer | 44 | 0.0846 | 1 | 0.0816 |
| mTOR signaling pathway | 45 | 0.0865 | 1 | 0.0834 |
| Amoebiasis | 46 | 0.0885 | 1 | 0.0851 |
| Shigellosis | 47 | 0.0904 | 1 | 0.0869 |
| MAPK signaling pathway | 265 | 0.51 | 2 | 0.0884 |
| NOD-like receptor signaling pathway | 49 | 0.0942 | 1 | 0.0905 |
| RIG-I-like receptor signaling pathway | 49 | 0.0942 | 1 | 0.0905 |
| Colorectal cancer | 49 | 0.0942 | 1 | 0.0905 |
| Leishmaniasis | 51 | 0.0981 | 1 | 0.094 |
| Pertussis | 52 | 0.1 | 1 | 0.0957 |
| Renal cell carcinoma | 60 | 0.115 | 1 | 0.11 |
| Salmonella infection | 72 | 0.138 | 1 | 0.13 |
| B cell receptor signaling pathway | 75 | 0.144 | 1 | 0.135 |
| Apoptosis | 83 | 0.16 | 1 | 0.149 |
| TGF-beta signaling pathway | 84 | 0.162 | 1 | 0.151 |
| ErbB signaling pathway | 87 | 0.167 | 1 | 0.156 |
| Prostate cancer | 87 | 0.167 | 1 | 0.156 |
| Chagas disease (American trypanosomiasis) | 89 | 0.171 | 1 | 0.159 |
| Toll-like receptor signaling pathway | 97 | 0.187 | 1 | 0.172 |
| T cell receptor signaling pathway | 98 | 0.188 | 1 | 0.174 |
| Hepatitis C | 100 | 0.192 | 1 | 0.177 |
| Melanogenesis | 101 | 0.194 | 1 | 0.178 |
| Herpes simplex infection | 103 | 0.198 | 1 | 0.182 |
| Influenza A | 107 | 0.206 | 1 | 0.188 |
| Osteoclast differentiation | 119 | 0.229 | 1 | 0.207 |
| Neurotrophin signaling pathway | 123 | 0.237 | 1 | 0.213 |
| Cell cycle | 124 | 0.238 | 1 | 0.215 |
| Wnt signaling pathway | 144 | 0.277 | 1 | 0.245 |
| Alcoholism | 166 | 0.319 | 1 | 0.278 |
| HTLV-I infection | 199 | 0.383 | 1 | 0.324 |

**Table S4c. Reactome Pathway Enrichment Analysis of Co-DEmiRNA-TF Network.**

| Reactome | Total | Expected | Hits | Pval |
| --- | --- | --- | --- | --- |
| Pathways in cancer | 310 | 0.596 | 5 | 0.000125 |
| Legionellosis | 40 | 0.0769 | 2 | 0.00247 |
| Renal cell carcinoma | 60 | 0.115 | 2 | 0.00549 |
| Adipocytokine signaling pathway | 63 | 0.121 | 2 | 0.00604 |
| Apoptosis | 83 | 0.16 | 2 | 0.0103 |
| TGF-beta signaling pathway | 84 | 0.162 | 2 | 0.0106 |
| MAPK signaling pathway | 265 | 0.51 | 3 | 0.0117 |
| Chagas disease (American trypanosomiasis) | 89 | 0.171 | 2 | 0.0118 |
| Toxoplasmosis | 93 | 0.179 | 2 | 0.0128 |
| Toll-like receptor signaling pathway | 97 | 0.187 | 2 | 0.0139 |
| Hepatitis C | 100 | 0.192 | 2 | 0.0148 |
| Herpes simplex infection | 103 | 0.198 | 2 | 0.0156 |
| Osteoclast differentiation | 119 | 0.229 | 2 | 0.0205 |
| Neurotrophin signaling pathway | 123 | 0.237 | 2 | 0.0219 |
| Transcriptional misregulation in cancer | 19 | 0.0365 | 1 | 0.036 |
| Cytosolic DNA-sensing pathway | 20 | 0.0385 | 1 | 0.0378 |
| Tuberculosis | 174 | 0.335 | 2 | 0.0417 |
| African trypanosomiasis | 25 | 0.0481 | 1 | 0.0471 |
| HTLV-I infection | 199 | 0.383 | 2 | 0.0532 |
| Epithelial cell signaling in Helicobacter pylori infection | 37 | 0.0712 | 1 | 0.069 |
| Amyotrophic lateral sclerosis (ALS) | 39 | 0.075 | 1 | 0.0726 |
| Cocaine addiction | 43 | 0.0827 | 1 | 0.0798 |
| Cytokine-cytokine receptor interaction | 253 | 0.487 | 2 | 0.0815 |
| Amoebiasis | 46 | 0.0885 | 1 | 0.0851 |
| Shigellosis | 47 | 0.0904 | 1 | 0.0869 |
| Basal cell carcinoma | 47 | 0.0904 | 1 | 0.0869 |
| Type II diabetes mellitus | 48 | 0.0923 | 1 | 0.0887 |
| NOD-like receptor signaling pathway | 49 | 0.0942 | 1 | 0.0905 |
| RIG-I-like receptor signaling pathway | 49 | 0.0942 | 1 | 0.0905 |
| Leishmaniasis | 51 | 0.0981 | 1 | 0.094 |
| Pertussis | 52 | 0.1 | 1 | 0.0957 |
| Hedgehog signaling pathway | 56 | 0.108 | 1 | 0.103 |
| Acute myeloid leukemia | 57 | 0.11 | 1 | 0.105 |
| Antigen processing and presentation | 61 | 0.117 | 1 | 0.111 |
| p53 signaling pathway | 68 | 0.131 | 1 | 0.124 |
| Melanoma | 68 | 0.131 | 1 | 0.124 |
| Pancreatic cancer | 69 | 0.133 | 1 | 0.125 |
| Salmonella infection | 72 | 0.138 | 1 | 0.13 |
| Chronic myeloid leukemia | 73 | 0.14 | 1 | 0.132 |
| B cell receptor signaling pathway | 75 | 0.144 | 1 | 0.135 |
| Fc epsilon RI signaling pathway | 75 | 0.144 | 1 | 0.135 |
| Small cell lung cancer | 80 | 0.154 | 1 | 0.144 |
| Prostate cancer | 87 | 0.167 | 1 | 0.156 |
| Epstein-Barr virus infection | 91 | 0.175 | 1 | 0.162 |
| T cell receptor signaling pathway | 98 | 0.188 | 1 | 0.174 |
| Measles | 102 | 0.196 | 1 | 0.18 |
| Influenza A | 107 | 0.206 | 1 | 0.188 |
| Protein processing in endoplasmic reticulum | 129 | 0.248 | 1 | 0.223 |
| Natural killer cell mediated cytotoxicity | 138 | 0.265 | 1 | 0.236 |
| Alcoholism | 166 | 0.319 | 1 | 0.278 |
| Chemokine signaling pathway | 189 | 0.363 | 1 | 0.31 |

**Table S4d.Gene Ontology** - **Biological Process Enrichment Analysis of Co-DEmiRNA-TF Network.**

| GO-BP | Total | Expected | Hits | Pval |
| --- | --- | --- | --- | --- |
| positive regulation of transcription from RNA polymerase II promoter | 800 | 0.672 | 10 | 1.71e-11 |
| positive regulation of transcription,DNA-dependent | 1260 | 1.06 | 10 | 1.52e-09 |
| positive regulation of transcription,DNA-dependent | 1260 | 1.06 | 10 | 1.52e-09 |
| positive regulation of RNA metabolic process | 1330 | 1.12 | 10 | 2.68e-09 |
| positive regulation of nucleobase-containing compound metabolic process | 1490 | 1.25 | 10 | 8.02e-09 |
| regulation of transcription from RNA polymerase II promoter | 1610 | 1.35 | 10 | 1.69e-08 |
| positive regulation of cellular metabolic process | 2530 | 2.12 | 11 | 5.23e-08 |
| transcription from RNA polymerase II promoter | 1930 | 1.62 | 10 | 1e-07 |
| positive regulation of metabolic process | 2690 | 2.26 | 11 | 1.04e-07 |
| regulation of multicellular organismal process | 2480 | 2.08 | 10 | 1.14e-06 |
| regulation of cell differentiation | 1290 | 1.08 | 8 | 1.53e-06 |
| regulation of developmental process | 1880 | 1.58 | 9 | 1.74e-06 |
| positive regulation of cell differentiation | 571 | 0.48 | 6 | 2.98e-06 |
| regulation of transcription,DNA-dependent | 3770 | 3.17 | 11 | 3.91e-06 |
| regulation of transcription,DNA-dependent | 3770 | 3.17 | 11 | 3.91e-06 |
| regulation of transcription,DNA-dependent | 3770 | 3.17 | 11 | 3.91e-06 |
| positive regulation of signal transduction | 998 | 0.838 | 7 | 4.61e-06 |
| regulation of RNA metabolic process | 3900 | 3.28 | 11 | 5.65e-06 |
| hemopoiesis | 640 | 0.538 | 6 | 5.78e-06 |
| embryo development | 1080 | 0.91 | 7 | 8.01e-06 |
| hematopoietic or lymphoid organ development | 679 | 0.57 | 6 | 8.14e-06 |
| negative regulation of nucleobase-containing compound metabolic process | 1130 | 0.95 | 7 | 1.06e-05 |
| negative regulation of cellular metabolic process | 1660 | 1.4 | 8 | 1.07e-05 |
| immune system development | 722 | 0.606 | 6 | 1.16e-05 |
| negative regulation of cellular biosynthetic process | 1220 | 1.03 | 7 | 1.77e-05 |
| positive regulation of cell proliferation | 786 | 0.66 | 6 | 1.89e-05 |
| negative regulation of biosynthetic process | 1240 | 1.04 | 7 | 1.98e-05 |
| response to organic substance | 2500 | 2.1 | 9 | 2e-05 |
| negative regulation of metabolic process | 1820 | 1.53 | 8 | 2.12e-05 |
| positive regulation of developmental process | 817 | 0.686 | 6 | 2.35e-05 |
| regulation of gene expression | 4480 | 3.77 | 11 | 2.46e-05 |
| regulation of nucleobase-containing compound metabolic process | 4540 | 3.81 | 11 | 2.79e-05 |
| positive regulation of protein modification process | 867 | 0.728 | 6 | 3.3e-05 |
| response to abiotic stimulus | 876 | 0.736 | 6 | 3.5e-05 |
| response to hypoxia | 245 | 0.206 | 4 | 3.75e-05 |
| regulation of cellular metabolic process | 6120 | 5.14 | 12 | 3.81e-05 |
| negative regulation of cell differentiation | 540 | 0.454 | 5 | 4.81e-05 |
| positive regulation of cellular process | 4780 | 4.01 | 11 | 4.84e-05 |
| negative regulation of transcription from RNA polymerase II promoter | 552 | 0.464 | 5 | 5.34e-05 |
| transcription,DNA-dependent | 4830 | 4.06 | 11 | 5.47e-05 |
| transcription,DNA-dependent | 4830 | 4.06 | 11 | 5.47e-05 |
| positive regulation of cellular protein metabolic process | 968 | 0.813 | 6 | 6.17e-05 |
| inflammatory response | 569 | 0.478 | 5 | 6.18e-05 |
| response to oxidative stress | 279 | 0.234 | 4 | 6.22e-05 |
| RNA biosynthetic process | 4930 | 4.14 | 11 | 6.74e-05 |
| negative regulation of transcription, DNA-dependent | 987 | 0.829 | 6 | 6.89e-05 |
| negative regulation of transcription, DNA-dependent | 987 | 0.829 | 6 | 6.89e-05 |
| response to chemical stimulus | 3830 | 3.22 | 10 | 7.2e-05 |
| negative regulation of RNA metabolic process | 1020 | 0.86 | 6 | 8.47e-05 |
| macromolecule biosynthetic process | 6540 | 5.5 | 12 | 8.47e-05 |
| macromolecule biosynthetic process | 6540 | 5.5 | 12 | 8.47e-05 |
| positive regulation of response to stimulus | 1550 | 1.3 | 7 | 8.54e-05 |
| regulation of cellular protein metabolic process | 1560 | 1.31 | 7 | 8.72e-05 |
| positive regulation of multicellular organismal process | 649 | 0.545 | 5 | 0.000116 |
| positive regulation of protein metabolic process | 1080 | 0.91 | 6 | 0.000116 |
| negative regulation of developmental process | 674 | 0.566 | 5 | 0.000138 |
| negative regulation of apoptotic process | 679 | 0.57 | 5 | 0.000143 |
| negative regulation of apoptotic process | 679 | 0.57 | 5 | 0.000143 |
| negative regulation of programmed cell death | 691 | 0.58 | 5 | 0.000155 |
| regulation of metabolic process | 6920 | 5.81 | 12 | 0.000166 |
| regulation of signal transduction | 2440 | 2.05 | 8 | 0.000182 |
| positive regulation of MAPK cascade | 368 | 0.309 | 4 | 0.000182 |
| positive regulation of biological process | 5500 | 4.62 | 11 | 0.000211 |
| regulation of response to stimulus | 3360 | 2.82 | 9 | 0.000235 |
| regulation of protein metabolic process | 1820 | 1.53 | 7 | 0.000242 |
| regulation of protein modification process | 1250 | 1.05 | 6 | 0.000255 |
| cellular nitrogen compound biosynthetic process | 5610 | 4.71 | 11 | 0.00026 |
| leukocyte differentiation | 404 | 0.339 | 4 | 0.00026 |
| response to wounding | 1310 | 1.1 | 6 | 0.000338 |
| gliogenesis | 176 | 0.148 | 3 | 0.000372 |
| developmental maturation | 177 | 0.149 | 3 | 0.000379 |
| steroid biosynthetic process | 183 | 0.154 | 3 | 0.000418 |
| skeletal system development | 459 | 0.385 | 4 | 0.000424 |
| positive regulation of sequence-specific DNA binding transcription factor activity | 198 | 0.166 | 3 | 0.000526 |
| regulation of cell proliferation | 1430 | 1.2 | 6 | 0.000536 |
| RNA metabolic process | 6010 | 5.05 | 11 | 0.000538 |
| tube development | 506 | 0.425 | 4 | 0.000613 |
| regulation of apoptotic process | 1540 | 1.29 | 6 | 0.000793 |
| regulation of programmed cell death | 1550 | 1.3 | 6 | 0.000845 |
| signal transduction | 6310 | 5.3 | 11 | 0.000886 |
| regulation of MAPK cascade | 559 | 0.469 | 4 | 0.000893 |
| positive regulation of translation | 56 | 0.047 | 2 | 0.000971 |
| regulation of lipid metabolic process | 246 | 0.207 | 3 | 0.000989 |
| negative regulation of cell proliferation | 585 | 0.491 | 4 | 0.00106 |
| biosynthetic process | 8090 | 6.79 | 12 | 0.00108 |
| cellular response to stress | 1620 | 1.36 | 6 | 0.00108 |
| cell fate commitment | 254 | 0.213 | 3 | 0.00108 |
| lymphocyte differentiation | 260 | 0.218 | 3 | 0.00116 |
| cellular carbohydrate catabolic process | 62 | 0.0521 | 2 | 0.00119 |
| negative regulation of cellular process | 4110 | 3.45 | 9 | 0.0012 |
| positive regulation of cytokine production | 268 | 0.225 | 3 | 0.00127 |
| response to stress | 4150 | 3.49 | 9 | 0.00131 |
| organ development | 3290 | 2.76 | 8 | 0.00157 |
| glucose metabolic process | 290 | 0.244 | 3 | 0.00159 |
| myeloid cell differentiation | 296 | 0.249 | 3 | 0.00169 |
| MAPK cascade | 665 | 0.559 | 4 | 0.00171 |
| regulation of endothelial cell proliferation | 79 | 0.0663 | 2 | 0.00192 |
| negative regulation of transport | 318 | 0.267 | 3 | 0.00207 |
| cell development | 1840 | 1.55 | 6 | 0.00211 |
| protein modification by small protein conjugation | 713 | 0.599 | 4 | 0.00221 |

**Table S4e. Gene Ontology-Molecular Functions Enrichment Analysis of Co-DEmiRNA-TF Network.**

| Pathway | Total | Expected | Hits | Pval |
| --- | --- | --- | --- | --- |
| positive regulation of transcription, DNA-dependent | 1260 | 1.04 | 10 | 1.33e-09 |
| transcription from RNA polymerase II promoter | 1930 | 1.6 | 10 | 8.8e-08 |
| sequence-specific DNA binding | 732 | 0.607 | 7 | 5.2e-07 |
| transcription factor binding | 509 | 0.422 | 6 | 1.41e-06 |
| DNA binding | 2760 | 2.29 | 10 | 2.8e-06 |
| negative regulation of transcription, DNA-dependent | 987 | 0.818 | 6 | 6.39e-05 |
| chromatin binding | 338 | 0.28 | 3 | 0.00237 |
| protein heterodimerization activity | 374 | 0.31 | 3 | 0.00316 |
| double-stranded DNA binding | 149 | 0.123 | 2 | 0.00649 |
| protein dimerization activity | 996 | 0.825 | 4 | 0.00704 |
| growth factor activity | 170 | 0.141 | 2 | 0.00837 |
| transcription cofactor activity | 552 | 0.457 | 3 | 0.00937 |
| protein binding transcription factor activity | 600 | 0.497 | 3 | 0.0118 |
| enzyme binding | 1200 | 0.994 | 4 | 0.0135 |
| cytokine activity | 220 | 0.182 | 2 | 0.0137 |
| structure-specific DNA binding | 242 | 0.201 | 2 | 0.0164 |
| transcription coactivator activity | 312 | 0.259 | 2 | 0.0265 |
| protein deacetylase activity | 39 | 0.0323 | 1 | 0.0319 |
| receptor binding | 1590 | 1.32 | 4 | 0.0351 |
| identical protein binding | 910 | 0.754 | 3 | 0.0355 |
| protein kinase binding | 376 | 0.312 | 2 | 0.0374 |
| damaged DNA binding | 46 | 0.0381 | 1 | 0.0375 |
| deacetylase activity | 52 | 0.0431 | 1 | 0.0423 |
| kinase binding | 418 | 0.346 | 2 | 0.0453 |
| histone deacetylase binding | 65 | 0.0539 | 1 | 0.0526 |
| SMAD binding | 68 | 0.0563 | 1 | 0.0549 |
| RNA polymerase II transcription cofactor activity | 73 | 0.0605 | 1 | 0.0589 |
| hydrolase acting on carbon-nitrogen (but not peptide) bondsactivity, in linear amides | 76 | 0.063 | 1 | 0.0612 |
| phosphatase regulator activity | 80 | 0.0663 | 1 | 0.0643 |
| protein domain specific binding | 560 | 0.464 | 2 | 0.0762 |
| RNA polymerase II distal enhancer sequence-specific DNA binding transcription factor activity | 110 | 0.0912 | 1 | 0.0875 |
| hydrolase acting on carbon-nitrogen (but not peptide) bondsactivity, | 147 | 0.122 | 1 | 0.115 |
| oxidoreductase activity, acting on the CH-OH group of donors, NAD or NADP as acceptor | 164 | 0.136 | 1 | 0.128 |
| oxidoreductase activity, acting on CH-OH group of donors | 182 | 0.151 | 1 | 0.141 |
| transcription corepressor activity | 208 | 0.172 | 1 | 0.159 |
| cytokine receptor binding | 266 | 0.22 | 1 | 0.2 |
| enzyme activator activity | 436 | 0.361 | 1 | 0.307 |
| transport | 4830 | 4.01 | 5 | 0.37 |
| protein homodimerization activity | 573 | 0.475 | 1 | 0.384 |
| oxidoreductase activity | 1010 | 0.837 | 1 | 0.58 |
| enzyme regulator activity | 1140 | 0.946 | 1 | 0.627 |
| ATP binding | 1490 | 1.23 | 1 | 0.728 |
| adenyl ribonucleotide binding | 1530 | 1.26 | 1 | 0.737 |
| adenyl nucleotide binding | 1530 | 1.27 | 1 | 0.738 |
| purine ribonucleotide binding | 1890 | 1.57 | 1 | 0.814 |
| purine nucleotide binding | 1900 | 1.57 | 1 | 0.815 |
| receptor activity | 1920 | 1.59 | 1 | 0.819 |
| zinc ion binding | 2010 | 1.67 | 1 | 0.834 |
| transition metal ion binding | 2270 | 1.88 | 1 | 0.871 |
| nucleotide binding | 2470 | 2.05 | 1 | 0.894 |
| cation binding | 4160 | 3.45 | 2 | 0.9 |
| ion binding | 6140 | 5.09 | 3 | 0.939 |

**Table S4f. Gene Ontology-Cellular Component Enrichment Analysis of Co-DEmiRNA-TF Network.**

| GO-CC | Total | Expected | Hits | Pval |
| --- | --- | --- | --- | --- |
| transcription factor complex | 303 | 0.218 | 4 | 4.72e-05 |
| nucleoplasm | 1820 | 1.31 | 7 | 8.93e-05 |
| nucleoplasm part | 910 | 0.655 | 5 | 0.000275 |
| nuclear lumen | 2690 | 1.94 | 7 | 0.00106 |
| chromatin | 326 | 0.235 | 3 | 0.00143 |
| nuclear part | 3330 | 2.4 | 7 | 0.00388 |
| organelle lumen | 3380 | 2.44 | 7 | 0.00426 |
| membrane-enclosed lumen | 3440 | 2.48 | 7 | 0.00472 |
| chromosomal part | 670 | 0.482 | 3 | 0.0108 |
| chromosome | 784 | 0.564 | 3 | 0.0166 |
| cytosol | 2660 | 1.92 | 5 | 0.0308 |
| histone deacetylase complex | 53 | 0.0382 | 1 | 0.0375 |
| nucleus | 7600 | 5.47 | 9 | 0.0391 |
| protein complex | 4050 | 2.92 | 6 | 0.0479 |
| cell surface | 518 | 0.373 | 2 | 0.0518 |
| macromolecular complex | 4800 | 3.45 | 6 | 0.0992 |
| nuclear chromatin | 159 | 0.114 | 1 | 0.109 |
| sarcomere | 163 | 0.117 | 1 | 0.111 |
| contractile fiber part | 187 | 0.135 | 1 | 0.127 |
| membrane raft | 189 | 0.136 | 1 | 0.128 |
| non-membrane-bounded organelle | 3940 | 2.84 | 5 | 0.131 |
| intracellular non-membrane-bounded organelle | 3940 | 2.84 | 5 | 0.131 |
| myofibril | 197 | 0.142 | 1 | 0.133 |
| extracellular space | 901 | 0.649 | 2 | 0.135 |
| external side of plasma membrane | 204 | 0.147 | 1 | 0.137 |
| contractile fiber | 214 | 0.154 | 1 | 0.144 |
| nuclear chromosome part | 273 | 0.197 | 1 | 0.18 |
| nuclear chromosome | 320 | 0.23 | 1 | 0.208 |
| intracellular organelle part | 8620 | 6.2 | 8 | 0.228 |
| extracellular region part | 1320 | 0.95 | 2 | 0.245 |
| organelle part | 8790 | 6.33 | 8 | 0.251 |
| centrosome | 412 | 0.297 | 1 | 0.259 |
| actin cytoskeleton | 430 | 0.31 | 1 | 0.269 |
| cytoskeletal part | 1570 | 1.13 | 2 | 0.315 |
| microtubule organizing center | 543 | 0.391 | 1 | 0.328 |
| synapse | 558 | 0.402 | 1 | 0.335 |
| extracellular region | 2860 | 2.06 | 3 | 0.34 |
| nucleolus | 652 | 0.469 | 1 | 0.381 |
| endosome | 683 | 0.492 | 1 | 0.395 |
| mitochondrion | 2060 | 1.48 | 2 | 0.446 |
| cytoskeleton | 2200 | 1.58 | 2 | 0.483 |
| microtubule cytoskeleton | 1120 | 0.805 | 1 | 0.565 |
| integral to plasma membrane | 1270 | 0.914 | 1 | 0.614 |
| cytoplasmic part | 9740 | 7.01 | 7 | 0.623 |
| intrinsic to plasma membrane | 1320 | 0.952 | 1 | 0.629 |
| cytoplasm | 13100 | 9.46 | 9 | 0.762 |
| plasma membrane part | 2320 | 1.67 | 1 | 0.834 |
| plasma membrane | 5500 | 3.96 | 2 | 0.943 |
| integral to membrane | 5590 | 4.02 | 1 | 0.993 |
| intrinsic to membrane | 5760 | 4.15 | 1 | 0.994 |
| membrane part | 7520 | 5.42 | 1 | 0.999 |
| membrane | 11700 | 8.44 | 2 | 1 |

**Table S5. Co-DEmiRNA-Compound Minimum Network-Interactions.**

| Id | Label | Degree | Betweenness |
| --- | --- | --- | --- |
| 5-fluorouracil | 5-fluorouracil | 5 | 77.16666667 |
| hsa-mir-31 | hsa-mir-31 | 4 | 62.66666667 |
| hsa-mir-224 | hsa-mir-224 | 3 | 50.75 |
| hsa-mir-497 | hsa-mir-497 | 3 | 19.83333333 |
| hsa-mir-210 | hsa-mir-210 | 3 | 30.66666667 |
| Ginsenoside Rh2 | Ginsenoside Rh2 | 3 | 27.16666667 |
| Formaldehyde | Formaldehyde | 3 | 29.16666667 |
| hsa-mir-486-5p | hsa-mir-486-5p | 2 | 8.166666667 |
| hsa-mir-33a | hsa-mir-33a | 2 | 10.41666667 |
| hsa-mir-363 | hsa-mir-363 | 2 | 5.5 |
| Cisplatin | Cisplatin | 2 | 17.5 |
| Vincristine | Vincristine | 2 | 8.916666667 |
| Trichostatin A (TSA) | Trichostatin A (TSA) | 2 | 13 |
| Arsenic trioxide | Arsenic trioxide | 2 | 7.5 |
| Enoxacin | Enoxacin | 2 | 18 |
| Diethylstilbestrol | Diethylstilbestrol | 2 | 8.583333333 |
| hsa-mir-140-3p | hsa-mir-140-3p | 1 | 0 |
| hsa-mir-1246 | hsa-mir-1246 | 1 | 0 |
| hsa-mir-29c | hsa-mir-29c | 1 | 0 |
| hsa-mir-31* | hsa-mir-31* | 1 | 0 |
